# Best practices for designing interventions to improve health worker performance in low- and middle-income countries

**DOI:** 10.7189/jogh.16.03027

**Published:** 2026-09-25

**Authors:** Alexander K Rowe, Elisa Roma, Naina J Ahuja, Alice E Pope, Karen Zamboni, Pooja Yerramilli, Samantha Y Rowe, Zunera Gilani, Stephanie Kandasami, Tove K Ryman, Katja Schemionek

**Affiliations:** 1 Independent Consultant, Atlanta, USA; 2 Country Delivery Department, Gavi, the Vaccine Alliance, Geneva, Switzerland; 3 Global Programme Division, UNICEF, New York City, USA; 4 Global Immunization Division, Centers for Disease Control and Prevention, Atlanta, USA; 5 Technical Advice & Partnerships, The Global Fund to Fight AIDS, Tuberculosis and Malaria, Geneva, Switzerland; 6 Department of Global Public Health, Karolinska Institutet, Stockholm, Sweden; 7 Health, Nutrition and Population, World Bank, Washington, D.C., USA; 8 Public Health Institute, Via United States Agency for International Development’s Global Health Training, Advisory and Support Contract Project, Washington, D.C., USA; 9 Gates Foundation, Seattle, USA

**Keywords:** developing countries, health workers, provider performance, quality improvement

## Abstract

Effective health worker (HW) performance is essential for high-quality healthcare. Yet, many low- and middle-income countries rely on HW performance improvement (HWPI) interventions that often yield modest results, such as stand-alone in-service HW training. This viewpoint identifies more effective HWPI interventions, such as group problem-solving. To encourage use of these approaches, we propose definitions of more effective HWPI interventions and what constitutes a high-quality HWPI investment (*i.e.* support for a particular intervention in a given context). We examine intervention effectiveness from a large systematic review as part of these definitions. We also present 13 best practices for intervention design and show how HWPI investments could be developed to achieve larger, sustained improvements in HW performance in real-world settings. Some best practices include a minimum expected effectiveness of 15 percentage points above baseline performance and basing the design on a situational analysis. We have formatted the best practices into a simple checklist tool for countries to inform their HWPI actions, including those proposed in grant applications, and for donors when reviewing grants. Our overarching goal is to optimise intervention effectiveness and improve HW performance and healthcare quality.

Health workers (HWs), including facility- and community-based providers, are essential for delivering healthcare. However, in low- and middle-income countries (LMICs), HW performance often falls short of what is needed to ensure high-quality care, largely due to systemic barriers, resource constraints, and working conditions that hinder optimal service delivery [1–5]. For example, mothers and children at a typical clinic visit receive less than half of recommended preventive or curative actions [1]. Globally, poor-quality care causes an estimated 4.9–8.4 million deaths each year [1,6]. To improve HW performance, countries typically implement training and supervision interventions [7–12] (personal communications from Karen Zamboni, 18 October 2024, and Elisa Roma, 20 November 2024).

These large-scale training and supervision efforts reflect genuine and sustained country and donor commitment to strengthening HW capacity. Nonetheless, when delivered as stand-alone interventions, they have shown limited effectiveness in improving performance outcomes. A systematic review of 60 LMIC study comparisons found that in-service training as a stand-alone intervention improved HW performance, in terms of clinical guideline adherence, by a median of 7.3 percentage points (interquartile range (IQR) = 3.6, 17.4); and these modest improvements tended to wane over time [9–13]. Therefore, if baseline HW performance is 50%, then typical post-training performance would be just 57.3% (based on the IQR, performance might vary from 53.6% to 67.4%). Similarly, the median effectiveness of supportive supervision was 10.7 percentage points (IQR = 6.9, 27.9) [10]. Nationally representative surveys in eight LMICs found no meaningful effect of training and supervision, as implemented by real-world programmes [7,14].

Fortunately, more effective HW performance improvement (HWPI) interventions exist, such as group problem-solving and an integrated combination of training, supervision, and providing medicines (**Table 1**; Boxes S1 and S2 in the **Online Supplementary Document**) [2,16]. However, these interventions are underutilised [11,17] (personal communication from Karen Zamboni, 18 October 2024). Possible reasons include unfamiliarity with these interventions, a lack of local capacity and funding to deliver them, and preference for the status quo. One obstacle is that no clear definition exists for ‘more effective’ interventions. Additionally, simply selecting one of these interventions based on research results is insufficient for success. Other design and implementation issues must be considered (*e.g.* local adaptation, country buy-in and capacity, and monitoring). Current guidance on this topic, while helpful, could be expanded and made more practical [18–21].

**Table 1:** Effectiveness of interventions to improve health worker performance in low- and middle-income countries, based on acceptable evidence from a large systematic review (Box S1 in the **Online Supplementary Document**)

	Analysis of all studies (all risk of bias levels)			Analysis of higher quality studies (*i.e.* only low or moderate risk of bias)*		
	**Number of study comparisons**	**Median effectiveness, percentage points (interquartile range)**	**Quality of evidence†**	**Number of study comparisons**	**Median effectiveness, percentage points (interquartile range)**	**Quality of evidence**
**Interventions for professional health workers (*e.g.* physicians, nurses, and midwives)**						
Interventions with median effectiveness greater than the threshold of 15 percentage points						
*Strengthening infrastructure plus supervision*‡ *plus other management techniques plus training*	5	69.4 (47.5, 297.1)	Moderate			
*Group problem-solving plus training*	4	62.4 (47.0, 77.8)	Moderate			
*Strengthening infrastructure plus supervision plus training*	7	61.2 (8.0, 77.0)	Moderate	4	64.3 (32.1, 88.7)	High
*Group problem-solving only*	15	27.2 (8.1, 51.7)	Low			
*Community support plus supervision plus training*	4	19.6 (8.9, 24.1)	Low			
*Patient/client support plus training*	6	18.0 (2.8, 23.1)	Low			
Interventions with median effectiveness less than or equal to the threshold of 15 percentage points						
*Supervision plus other management techniques plus training*	6	12.6 (0.6, 31.0)	Low			
*Other management techniques plus training*	6	11.9 (9.9, 21.3)	Low			
*Health system financing (e.g. health insurance or removal of user fees)*	4	11.9 (−8.3, 46.4)	Low			
*Supervision only*	20	11.2 (4.8, 21.4)	Moderate	11	11.7 (2.4, 27.9)	High
*Supervision plus training*	27	10.5 (5.0, 35.3)	Very low	11	9.2 (4.5, 30.0)	Low
*Other management techniques only*	4	10.4 (0.7, 22.0)	Moderate			
*Training only*	78	9.7 (3.0, 20.3)	Low	33	9.0 (2.7, 19.8)	Moderate
*Strengthening infrastructure only*	4	7.1 (−3.5, 155.5)	Moderate	4	7.1 (−3.5, 155.5)	Moderate
*Information and communication technology (i.e. digital interventions) for health workers only*§	11	6.5 (−2.4, 15.8)	High	11	6.5 (−2.4, 15.8)	High
*Community support plus training*	5	6.1 (4.5, 10.2)	Low			
*Supervision plus other management techniques*	5	2.7 (1.6, 5.8)	Very low			
*Supervision plus printed information or job aids for health workers*	5	1.9 (−0.6, 16.7)	Low			
*Performance-based financing*§	16	1.8 (−0.7, 5.2)	Moderate	11	0.6 (−1.0, 3.9)	Moderate
*Printed information or job aids for health workers only*	8	1.6 (−0.4, 6.1)	Moderate	5	2.5 (0.8, 5.2)	High
**Interventions for lay health workers (*e.g.* community health workers)**						
Interventions with median effectiveness less than or equal to the threshold of 15 percentage points (no intervention had a median effectiveness greater than 15 percentage points based on acceptable evidence)						
*Information and communication technology (i.e. digital interventions) for health workers only*§	13	6.7 (2.9, 12.4)	High	13	6.7 (2.9, 12.4)	High
*Training only*	4	2.4 (−1.1, 7.4)	Very low			

*A blank row means the intervention was tested by fewer than four study comparisons with a low or moderate risk of bias.

†Based on the Grading of Recommendations Assessment, Development, and Evaluation (GRADE) system [15] (Text S4 in the **Online Supplementary Document**).

‡Supervision refers to supportive supervision and other similar interventions (*e.g.* audit with feedback); and training refers to any learning strategy, such as group in-service training, educational outreach, or peer-to-peer training.

§Results for this intervention come from a rapid review (Text S6 for performance-based financing, and Text S8 for information and communication technology in the **Online Supplementary Document**).

Our first objective is to address these gaps by proposing a definition of more effective interventions, while our second is to use this definition to specify what constitutes a high-quality HWPI investment, *i.e.* support for a particular intervention in a given context. The concept of a ‘more effective intervention’ relates to intervention effectiveness somewhat generically, with an emphasis on identifying interventions that are better than what countries typically use. A ‘high-quality HWPI investment’ goes one step further by guiding decision-making in a specific context, where local effectiveness evidence might be available or where there is a desire to use a novel intervention, whose effectiveness is unknown.

As part of these definitions, we present best practices for intervention design and implementation, and thus show how HWPI investments can be developed and assessed, in terms of how likely they will lead to larger, sustained improvements in HW performance in LMIC settings, compared to the *status quo*. We have formatted the best practices into a simple checklist tool for countries, donors, and other partners (Checklists S1 and S2 in the **Online Supplementary Document**). Here, ‘partners’ includes faith-based and non-governmental organisations, United Nations agencies (*e.g.* World Health Organization (WHO) and UNICEF), local professional associations, academic institutions, private sector actors, and others. Methodologically, the definitions and best practices were informed by published and unpublished literature; a reanalysis of a large systematic review (the Health Care Provider Performance Review (HCPPR)) (Box S1 in the **Online Supplementary Document**) [2,22]; three rapid reviews to capture recent evidence for topics of special interest (performance-based financing, low-dose, high-frequency training, and digital interventions); feedback from country-based staff; and our own experiences working with countries, donors, and other partners.

Countries and their partners could use the definitions, best practices, and checklist to inform their HWPI actions, including those proposed in grant applications, and donors when reviewing grants (or contracts and other financing arrangements). Over time, the best practices will evolve with growing evidence and innovation. The overarching goal is to strengthen investments and deliver sustained improvements in HW performance and the quality of care. This approach supports the goal of having country and donor investments that are evidence-based and that optimise value for money (including efficiency, economy, effectiveness, equity, and sustainability [23]), and supports principles articulated in recent declarations on human resources for health, universal health coverage, and reducing premature deaths [24–26].

## OBJECTIVE 1: DEFINITION OF MORE EFFECTIVE HWPI INTERVENTIONS

Most health technologies (*e.g.* medicines, vaccines, and insecticide-treated mosquito bed nets) have evidence-based standards for what constitutes acceptable widespread use, and donors utilise these standards to guide investments [27,28]. HWPI interventions in LMICs are a notable exception, although current donor funding guidelines and WHO guidance on community HWs and digital interventions contain valid recommendations [29–37]. Standards are based on evidence from systematic reviews [38,39], which are used to classify interventions (*e.g.* as recommended, recommended against, or insufficient evidence [39,40]).

The definition proposed here follows a similar approach to evidence gathering and intervention classification. First, though, the following terms require description.

### HW performance

HW performance refers to HW practices and the results of those behaviours [41], in terms of adherence to guidelines for clinical tasks (*e.g.* prevention, treatment, and counselling), people-centred health services (*e.g.* providing respectful care), and management actions (*e.g.* developing micro-plans for community outreach). The justification for this definition is that improved HW practices are moderately correlated with improved patient health outcomes (the ultimate goal of health programmes) (Pearson’s correlation = 0.7) [42–44] (Text S9 in the **Online Supplementary Document**), and many HWPI studies use HW practice outcomes. Improved HW knowledge, by itself, is not the same as improved HW performance, as the correlation between improved HW knowledge and improved practices is weak (data not published) [45]. Also, to focus the scope of this viewpoint, our definition of performance does not include measures of HW productivity (*e.g.* caseload) or worksite presence/absence.

### Acceptable evidence for assessing intervention effectiveness

Acceptable evidence means at least four study comparisons tested an HWPI intervention in an LMIC, with one of the following designs: controlled before-and-after study (with or without randomisation), post-intervention-only study with randomised controls, or interrupted time series. A study comparison is the difference between an intervention HW group and a no-intervention control group (or HWs exposed to a second intervention). These study designs are less susceptible to bias (*e.g.* compared to uncontrolled studies, which are often done in LMICs) and are used by expert systematic review groups [2,46,47]. The minimum of four study comparisons is somewhat arbitrary but allows for at least some generalisability, although evidence from more studies across a greater diversity of contexts is preferable (Text S1 in the **Online Supplementary Document**).

When interpreting and acting upon evidence on intervention effectiveness, one should also consider attributes such as study quality, consistency of findings, and contextual relevance (**Table 1**).

### Benchmark interventions

Benchmark interventions are HWPI interventions that countries often use and that typically have comparatively low effectiveness, which we aim to exceed. In other words, defining ‘more effective’ interventions requires a comparison group (*i.e.* more effective interventions are better than what?). We use effectiveness estimates from the HCPPR reanalysis and supplementary rapid reviews (Box S1 and Texts S6–8 in the **Online Supplementary Document**). The benchmark interventions are: stand-alone training (median effectiveness is 9.7 percentage points) and stand-alone supportive supervision (median effectiveness is 11.2 percentage points (**Table 1**; Text S3 in the **Online Supplementary Document**). ‘Stand-alone’ means not combined with other interventions.

### Best practices for intervention design and implementation

#### Best practice 1: the intervention has an expected median or mean effectiveness that is greater than 15 percentage points, based on acceptable evidence

This threshold reflects the overarching goal of encouraging use of interventions that are more effective than the benchmark interventions that countries typically rely on, with a median effectiveness of 10–11 percentage points. **Table 1** presents interventions that exceed the 15-percentage-point threshold from the HCPPR reanalysis (Box S1 in the **Online Supplementary Document**) and supplementary rapid reviews (Texts S6 and S8, and Spreadsheet S1 in the **Online Supplementary Document**). For example, the integrated combination of strengthening infrastructure, supervision, other management techniques, and training has a median improvement of 69.4 percentage points (Box S2 in the **Online Supplementary Document**). Alternatively, group problem-solving plus training has a median improvement of 62.4 percentage points, with one type (collaborative improvement plus training) being especially effective [16,48]. Importantly, the 15-percentage-point threshold is somewhat arbitrary, and programmes should try to maximise effectiveness. Although much of the effectiveness evidence comes from studies with short follow-up periods (Box S1 in the **Online Supplementary Document**), interventions should strive to improve HW performance and maintain the improvement. As context influences intervention effectiveness, when selecting interventions, it is ideal to examine results from studies in contexts similar to the one in which the intervention will be used (*e.g.* public *vs.* private sector facilities, or low *vs.* moderate resource settings [2]). **Table 1** also shows interventions with median effectiveness below the 15-percentage-point threshold. For countries considering a different minimum threshold of intervention effectiveness, the interventions are ordered from most to least effective. For example, if a threshold of 20 percentage points is desired, the first four interventions would meet the requirement.

#### Best practice 2: the design is based on a situational analysis

A situational analysis identifies performance problems (*i.e.* gaps between current and desired quality of care [49]), causes of those problems, and other information to select and tailor HWPI interventions, and thus increase the likelihood of success (Box S3 in the **Online Supplementary Document**). This undertaking, which could be comprehensive or focused, is recommended by WHO guidance on improving quality of care [5] and is the first step in change management and intervention scale-up models [19,50–52]. A situational analysis should be ‘right-sized’. For example, a large survey measuring high-quality information might be so time-consuming that it delays programmatic action. However, countries are heterogeneous, and it is important to understand subnational-level needs, especially for large countries. Situational analyses could be enhanced by focusing on HW needs, motivations, and experiences, as well as the structural and organisational drivers of HW behaviours [53]. When using assessment results, decision-makers should select interventions based on how they work (*i.e.* which problems they solve) and the problems (and their causes) in the setting where interventions will be used. For detailed examples of intervention selection, see Forbes *et al.*, Oliwa *et al.*, and Van Tiem *et al.* [54–56] (Box S4 in the **Online Supplementary Document**). A theory of change could show how interventions will address performance gaps [20,57–59], and sequencing interventions might be critical. For example, national vaccine stockouts should be addressed before implementing interventions to improve vaccine delivery. Interventions will also require contextual adaptation to local guidelines, systems (*e.g.* a digital tool’s interoperability with a country’s health information system), strategies (*e.g.* a national quality strategy), and other on-the-ground realities (*e.g.* where HWs are irregularly paid, user fee removal could cause informal payments). Although considerable literature exists on how situational analyses can inform intervention design, there is currently no consensus on the best way to tailor interventions and the effectiveness of the tailoring process [19,54,56,60–64].

#### Best practice 3: the intervention design and objectives are clearly described

Interventions should be described in detail, including who will deliver and receive the intervention. Hoffman *et al.* provide a template for such a description [65]. Intervention objectives should be closely aligned with programmatic goals and the performance gaps from the situational analysis. Objectives should be specific, measurable, achievable, relevant, and time-bound [66].

#### Best practice 4: there is evidence of country ownership

Country ownership is key [67,68], and without the support of leaders and other stakeholders, interventions might be perceived as donor-driven projects, which could impede sustainability (explained under best practice 9). Additionally, an unsupportive minister or programme manager could block implementation. When country and donor priorities diverge, the approach proposed here could serve as a framework for assembling evidence to compare the justification for competing priorities and help resolve differences objectively.

#### Best practice 5: the design involved HW input

Recognising HWs as agents of change, not just recipients of training, is essential to designing effective interventions. Their deep understanding of frontline realities is a critical asset, as are their views on the acceptability of HWPI interventions. Intervention designers could use human-centred design methods to elicit and use these perspectives [19,69–71]. Consulting with clients might be important, too [19]. Certain interventions explicitly involve HW input, such as group problem-solving, which could have large effect sizes [16]. One systematic review found no association between intervention effectiveness and whether HWs helped develop the intervention; however, this analysis was limited because HW input was often not reported by studies [2].

#### Best practice 6: the design builds on existing HPWI-relevant infrastructure and policies

Building on information systems, community networks, supervision structures, and partnerships with non-governmental organisations and donors, and policies (*e.g.* related to quality of care and continuing professional development) fosters intervention acceptability, efficiency, and sustainability, while coordination among partners, through a multi-stakeholder approach [72], reduces fragmentation [73–75].

#### Best practice 7: the country has the capacity to implement the intervention or a plan to develop the capacity

More effective interventions can be more complex than benchmark interventions. For example, collaborative improvement requires coaching, root cause analysis, self-monitoring, and plan-do-study-act cycles [16,48]. Country staff might lack these skills, and one cannot assume that short-term training and external technical assistance (a common approach) will be sufficient to deliver the intervention with high fidelity. Hence, it is crucial to demonstrate local capacity or to have robust plans to develop it. Countries might be particularly open to this kind of development, which could align with continuing professional development priorities or form part of a national quality strategy.

#### Best practice 8: the design has a realistic budget, in terms of funding and time

Often, HWPI interventions are insufficiently resourced. Common causes include costs of implementing interventions (especially in difficult-to-reach areas, or settings affected by conflict or humanitarian emergencies), strengthening the capacity to deliver them, developing information systems, conducting surveys, and obtaining approval. Insufficient budgets can weaken implementation and reduce effectiveness, even for interventions that research has found to be highly effective. When designing a realistic budget, it is essential to: actively seek domestic funding; distinguish costs for intervention introduction, scale-up, and maintenance; include funding for HW performance monitoring (explained under best practice 12); and recognise that there might be a culture of using *per diem* payments (*e.g.* from training and supervision) to supplement low HW salaries. On this latter point, when shifting to more effective interventions, countries and partners should critically evaluate the costs and benefits of *per diems*. While these payments may be considered necessary in low-salary settings, they should be considered carefully to avoid unintended effects. At the same time, such mechanisms can reflect the broader need to address financial and motivational challenges faced by HWs [76].

#### Best practice 9: the design includes a plan for scale-up and sustainability

Scale-up and long-term sustainability (or maintenance) are crucial elements for achieving impact that makes a difference [23,25]. Published models could help to develop a roadmap, which should realistically consider what is required, in terms of people, time, funding, infrastructure, and stakeholder ownership [50,52,77,78].

#### Best practice 10: for interventions that include HW training, the design uses evidence-based best practices

First, at least part of the training is done where HWs routinely work. A systematic review found that the mean effect of in-service training when some or all training occurred where HWs routinely work was 6.0–10.4 percentage points greater than when all training was done off-site [9]. On-site training allows HWs to adapt how they apply new skills to their specific workplace environment, and it may be less disruptive and less costly in terms of *per diem* payments. Second, the intervention includes practice of the skills taught in the training. The same training review found that in-service training that included clinical practice tended to be more effective than training without this method by 6.9–7.4 percentage points [9]. Practising skills in a safe learning environment before their application in a work setting is a basic adult learning principle. Third, as an extension of the previous two points, on-site training with practice that divides the training content into smaller packages that are delivered over two or more sessions (sometimes called low-dose, high-frequency training) had a median improvement of 17.5 percentage points for HW practices (Text S7 in the **Online Supplementary Document**). Fourth, the intervention uses competency-based education and other adult learning principles. Competency-based education means developing specific skills needed for one’s role, as opposed to theoretical training [79]. Adult learning principles (*e.g.* use of dialogue, case studies, sequencing, and informational feedback) are based on decades of education research [9,67,80,81] (Boxes S5 and S6 in the **Online Supplementary Document**).

#### Best practice 11: for interventions that include supervision, the design uses evidence-based best practices

First, supervision visits include problem-solving activities with HWs or use quality improvement methods. A systematic review found that the mean effect of supervision was 14.2–20.8 percentage points larger when supervisors participated in problem-solving with HWs (compared to no problem-solving), although this result had borderline statistical significance (*P*-values ranged from 0.032–0.098) [10]. Another review recommended that supervision use quality improvement tools and processes [82]. Second, the intervention includes supervision of supervisors. The mean effect of supervision was 8.8–11.5 percentage points larger when supervisors received supervision (compared to no supervision), although this result had borderline statistical significance (*P*-values ranged from 0.051–0.097) [10].

#### Best practice 12: the intervention’s implementation plan includes a system to monitor HW performance against stated objectives, assess intervention effect, and act upon this information

Monitoring is a cornerstone of any performance improvement effort, as it is difficult to predict how effective an HWPI intervention will be in a new context, and effectiveness measured under research conditions might not reflect results in a programmatic setting [5,19,20,83]. This conclusion is supported by systematic reviews that demonstrate wide heterogeneity in the effectiveness of specific interventions [2,46]. On a more practical level, monitoring data on HW performance can identify when interventions are underperforming and guide efforts to improve them. Data could help target interventions to areas with the largest performance gaps. Monitoring supports accountability mechanisms, and results could be used to show success and advocate for more resources. Results could be shared with HWs, with feedback becoming an intervention component. Monitoring could uncover unplanned intervention effects (positive or negative), and the process of monitoring reinforces the notion that improvement is an ongoing endeavour [84]. Ideally, monitoring is part of a country’s health management information system. For example, it could be conducted using routine records or supervisory checklists; however, rigorous surveys are helpful for assessing the validity of simpler data sources. Digital technologies can support real-time performance monitoring [85–87]. Monitoring should also assess intervention implementation strength (*i.e.* fidelity), which is important because interventions underperform when not fully implemented [85,88,89]. Implementation gaps should be identified and addressed early. Although a review of monitoring methods is outside the scope of this viewpoint, valid resources exist [90–94].

#### Best practice 13: There are specific actions to address broader health workforce issues

Many LMICs are challenged with HW shortages (14.7 million HWs in 2023, globally) [95], maldistribution across and within countries, management inefficiencies, and competency gaps from pre-service education. Key strategies to address these deficiencies include scaling up and strengthening pre-service education to meet health labour market needs; investing in jobs and decent working conditions, including adequate pay; optimising the skill mix; using planning tools; measurement; and strengthening institutional capacity to manage the health workforce [35,36,95–100]. Expanding availability, improving distribution, and optimising the performance of the health workforce can work synergistically to increase effective service coverage and improve health outcomes [101].

### Definition of HWPI interventions that are more effective than what is often done

We propose three effectiveness categories (**Box 1**): ‘more effective’ interventions (*i.e.* acceptable evidence shows they are more effective); ‘less effective’ interventions (*i.e.* evidence shows they are less effective); and interventions for which ‘more evidence is needed’ (*i.e.* effectiveness is currently not well characterised). More effective interventions have an expected improvement greater than 15 percentage points (*i.e.* exceeding benchmark interventions) and have a design and implementation plan that includes all other applicable 12 best practices described above. Less effective interventions either have an expected improvement of 15 percentage points or less, or lack any of the other applicable 12 best practices. Interventions in the third category lack acceptable evidence on effectiveness.

Box 1 **Definitions of categories of health worker performance improvement (HWPI) intervention effectiveness and investments**

*Categories of HWPI intervention effectiveness (Objective 1)*
  - **‘More effective’** HWPI interventions have **both** of the following criteria:
    - Typical effectiveness (median or mean) for improving health worker (HW) performance, based on acceptable evidence (as defined in this viewpoint article), is greater than 15 percentage points (*i.e.* better than benchmark interventions of stand-alone training and stand-alone supervision).
    - A design and implementation plan that includes **all** other applicable 12 best practices (items 2–13 in this viewpoint), such as a situational analysis, clear objectives, best practices for training and supervision (if applicable), and a plan for monitoring HW performance.
  - **‘Less effective’** HWPI interventions have **either** of the following criteria:
    - Typical effectiveness (median or mean) for improving HW performance, based on acceptable evidence, is 15 percentage points or less
    - A design or implementation plan that lacks any of the other applicable 12 best practices
  - HWPI interventions that **‘need more evidence’**
    - Typical effectiveness has not been characterised by acceptable evidence
*Categories of HWPI investments (Objective 2)*
  - HWPI investments that are **‘aligned with best practices’** have **any one** of the following three criteria:
    - Use of a ‘more effective’ intervention (defined above)
    - Use of a ‘needs more evidence’ intervention **and** the intervention design is based on **all** applicable best practices (items 2–13 in this viewpoint) **and** it is implemented in the context of a study to evaluate its effectiveness **and** the evaluation study will generate acceptable evidence (*e.g.* use of an acceptable study design)
    - Use of a ‘less effective’ intervention **and** there is a compelling reason for its utilisation that includes documented effectiveness greater than 15 percentage points in the context where it will be used **and** the intervention is designed and implemented with best practices (items 2–13 in this viewpoint)
  - HWPI investments that are **‘not aligned with best practices’** have none of the above three criteria.

## OBJECTIVE 2: DEFINITION OF HWPI INVESTMENTS THAT ARE ALIGNED WITH BEST PRACTICES

Thus far, we have considered intervention effectiveness in terms of typical (*e.g.* median) values; however, for a given intervention type, study results can vary substantially. For example, although the median effectiveness of stand-alone technology-based interventions is about 7 percentage points (**Table 1**), context-specific exceptions exist. For example, a trial in Kenya found text-message reminders sent to HWs’ phones improved HW treatment of malaria by 24 percentage points [102]. Thus, in Kenya, investing in this intervention might be a reasonable choice. Additionally, most existing HWPI interventions are understudied (*i.e.* the ‘need more evidence’ category), and new interventions are being developed. To foster new approaches, countries should be enabled to use such interventions in the context of an evaluation.

With these considerations, we propose two categories to describe whether investing in a particular intervention in a given context is justified (**Box 1**): ‘aligned with best practices’ means there is sufficient evidence or conditions (*i.e.* it is a high-quality investment), and ‘not aligned with best practices’ means insufficient evidence or conditions.

## LIMITATIONS

First, we based the evidence on intervention effectiveness on research studies, which have important limitations (Box S1 in the **Online Supplementary Document**). Thus, these findings might not predict improvements in non-research, programmatic settings. Similarly, since many studies had short follow-up times, it is unclear how effective some interventions will be over time and what the challenges related to long-term maintenance and institutionalisation might be. Effectiveness over time has been studied in some interventions, with certain interventions having waning effects (*e.g.* training), increasing effects (*e.g.* stand-alone group problem solving), and little change over time (*e.g.* group problem solving plus training) [13]. Second, the difference in effectiveness between any pair of interventions could be partly caused by differences in the outcomes and contexts of the studies that evaluated the interventions. Third, our evidence (**Table 1**) includes few macro-level interventions (*e.g.* performance-based financing) and meso-level interventions (*e.g.* collaborative improvement). While macro- and meso-level interventions (*e.g.* to address governance quality and political economy constraints) are likely important and interact with micro-level interventions (*e.g.* training) to impact HW performance, based on the HCPPR and the rapid reviews, there was relatively little acceptable evidence to allow for their inclusion. This does not mean they should not be considered for use; rather, they should be studied moving forward. Similarly, due to insufficient evidence, we do not cover broader actions for HWs that deserve attention, such as ensuring a living wage, adequate housing [103], mental health support, gender-sensitive HW policies [104], and safe working conditions (especially in conflict-affected settings [105,106]), as well as foundational elements such as developing a national quality-of-care policy and strategy [5], climate-resilient health systems [107,108], and digital interventions based on artificial intelligence. Fourth, our definition of HW performance excludes HW productivity or worksite presence/absence, which are important performance attributes. Fifth, we did not consider intervention cost and cost-effectiveness, as few studies reported on these important attributes. However, economic evaluations have been done for some more effective interventions [109,110]. Cost, which influences feasibility and scalability, is especially important as some of the more effective interventions are also more complex and potentially more expensive. Sixth, estimates of intervention effectiveness assumed that studies had approximately equal implementation strength (fidelity) and success in ‘matching’ interventions to the HW performance problems that needed to be solved and to the overall context. Bias could be introduced in between-intervention differences in effectiveness if certain interventions systematically had stronger or weaker implementation or were better or worse matched to the problem and context. Seventh, interventions might have unintended negative consequences, but little evidence exists on which interventions or settings are at higher risk for them [111]. Eighth, 13 best practices might seem daunting. However, each represents an opportunity to increase real-world impact.

## TAKING ACTION

Gaps in HW performance, often rooted in broader system challenges, can affect the quality of care and can be improved through a shift from stand-alone training and supervision to more effective, evidence-based HWPI interventions. To encourage this shift, we have presented best practices for designing HWPI interventions. These recommendations aim to strengthen, not replace, prior and ongoing investments in HW training and supervision. Lessons from past and current efforts offer a valuable foundation for adapting and strengthening interventions using more evidence-informed, integrated, and context-sensitive approaches.

The best practices checklist (Checklists S1 and S2 in the **Online Supplementary Document**) could be used by countries and partners (when planning HWPI activities and developing funding proposals) and by donors (when reviewing country funding proposals). This checklist could provide a basis for accountability and transparency across all stakeholder decision-making in this domain. To foster use of the best practices, decision-makers could mainstream their use (*e.g.* in donor funding guidelines), which would be analogous to the well-recognised approach of clinical guidelines that recommend only certain evidence-based treatments for a given health condition. Targets for using the best practices could be developed and monitored (*e.g.* average number of best practices per HWPI activity or grant). For interventions with multiple components, different stakeholders may choose to support specific intervention components while ensuring that all needed components are still implemented in an integrated fashion.

Importantly, the above-mentioned shift is not ‘anti-training’. Knowledge deficits can be a major cause of poor performance, and educational interventions are often justified. Rather, a key lesson is that country, partner, and donor thinking should change from ‘stand-alone workshop-based training’ to ‘training with better methods plus what else?’ [17], as the training effectiveness could be bolstered through best practices and complementary interventions (**Table 1**). Moreover, the shift can occur gradually (*e.g.* starting in one province to gain experience and fine-tune local adaptation).

Regarding the entire improvement endeavour, from situational analysis to intervention design to evaluation, further improvement, scale-up, and maintenance, countries and partners would benefit from considering improvement models from LMICs and the commercial sector from high-income countries [50–52,54,62].

The best practices should be revised based on the latest evidence every 3–5 years, which should include updated systematic reviews on intervention effectiveness, and other performance attributes described above. We have developed research priorities to inform future versions of the best practices (Text S5 in the **Online Supplementary Document**).

## CONCLUSIONS

We aim to spur action by country governments, donors, and other partners to use better interventions to improve HW performance and quality of care. We propose realistic best practices to complement and evolve traditional approaches, such as stand-alone training, towards more effective and context-responsive strategies that are more likely to achieve sustained improvements in performance and quality of care.

## Additional material


Online Supplementary Document

## Data Availability

**Data availability:** Data are available in the **Online Supplementary Document**.
