# Supplementary material for: Best practices for designing interventions to improve health worker performance in low- and middle-income countries
Source: J Glob Health. 2026 Sep 25;16:03027. doi: 10.7189/jogh.16.03027 (PMC13613295; doi:10.7189/jogh.16.03027)
Supplement: Supplementary file 1 [file jogh-16-03027-s001.zip › jogh-16-03027-s001.pdf]

Supplement to: Rowe AK, Roma E, Ahuja NJ, Pope AE, Zamboni K, Yerramilli P, Rowe SY, Gilani, Z, Kandasami S, Ryman TK, Schemionek K. Best practices for designing interventions to improve health worker performance in low- and middle-income countries. J Glob Health. 2026;16:03027.

Table of contents

Section 1. Additional methodological details and results

- **Box S1.** Brief description of the Health Care Provider Performance Review
- **Box S2.** Example of a more effective health worker performance improvement (HWPI) intervention
- **Box S3.** Essential elements of a situational analysis to inform an HWPI intervention
- **Box S4.** Case study of how a situational analysis was performed and used to design an intervention to improve HW performance for tuberculosis in Kenya
- **Boxes S5 and S6.** Best training practices based on adult learning principles
- **Text S1.** Additional detail on the definition of acceptable evidence
- **Text S2.** Additional detail on adjusted effect sizes
- **Text S3.** Supportive supervision: definition and its effect in multifaceted interventions
- **Text S4.** Interpretation of quality-of-evidence categories
- **Box S7.** Detailed description of intervention categories in **Table 1**
- **Table S1.** Effectiveness of interventions to improve performance of HWs in low- and middle-income countries (LMICs): sensitivity analysis adjusted for baseline performance
- **Table S2**. Effectiveness of interventions to improve performance of HWs in LMICs: interventions tested by at least three study comparisons.
- **Box S8.** Inconsistent results for interventions tested with three study comparisons

Section 2. Checklists for assessing HWPI investments based on definitions and best practices

- **Checklist S1.** Long version of the tool
- **Checklist S2.** Short version of the tool

Section 3. Research priorities related to HWPI interventions

- **Text S5.** List of research priorities

Section 4. Supplementary rapid reviews

- **Text S6.** Performance-based financing
- **Text S7.** Low-dose, high-frequency training
- **Text S8.** Information and communication technology

Section 5. The correlation between HW practice outcomes and patient health outcomes

- **Text S9.** Background, objective, methods, and results

Section 1. Additional methodological details and results

Box S1. Brief description of the Health Care Provider Performance Review (HCPPR), its reanalysis, and its limitations

- **Description.** The HCPPR is systematic review that aimed to characterize the effectiveness of all interventions to improve health worker (HW) performance in low- and middle-income countries (LMICs). Detailed methods and results are published elsewhere, and datasets and publications are available at the review's website (https://www.HCPperformancereview.org).[a,b] HWs were broadly defined as hospital-, clinic-, or community-based providers; pharmacists; and shopkeepers and informal vendors who sell medicines. Studies were included on any health condition, in any language, and from public and private sector settings. The literature search included 52 electronic databases for published studies and 58 document inventories for unpublished studies from the 1960s to 2016. Eligible study designs were controlled trials and interrupted time series. Effect sizes were calculated as absolute percentage-point changes (e.g., baseline-to-follow-up improvements in an intervention group minus baseline-to-follow-up improvements among controls). Intervention components were grouped into 12 mutually exclusive categories, such as training, supervision, and group problem-solving (see **Box S7** in the **Online Supplementary Document** for details); and an intervention was defined as any unique integrated combination of the 12 component categories. Here, "integrated" means that the interventions are implemented together, and all are focused on addressing a specific HW performance gap.
- **Reanalysis.** The HCPPR reanalysis included results of HW practice outcomes from the 332 LMIC studies in the HCPPR with at least one HW practice outcome. All effect sizes compared results of an intervention group to those of a no-intervention control group. To simplify the results and maximize the number of study comparisons per intervention, for each study comparison, a single summary measure was calculated as the median effect size for all HW practice outcomes, including outcomes expressed as percentages (e.g., percentage of patients treated correctly) and outcomes expressed as continuous measures (e.g., number of medicines prescribed per patient). To keep the reanalysis as simple as possible, the reanalysis presented in **Table 1** used unadjusted and unweighted effect sizes. **Table S1** in the **Online Supplementary Document** presents effectiveness adjusted for baseline performance (effectiveness tends to decrease as baseline increases).[a] **Text S2** in the **Online Supplementary Document** presents additional detail on the reanalysis.
- **Limitations.** The literature was last searched in 2016. Studies have different designs (many with a high risk of bias), often have short follow-up periods (i.e., 1–6 months), use different outcomes and data collection methods, and usually did not report intervention costs. Intervention categories include a variety of specific interventions that might have different effectiveness levels (e.g., the category "other management techniques" includes "HWs given job descriptions", "pre-packaging drugs", and many others). Interventions tested by fewer study comparisons probably have less external validity than interventions tested by more study comparisons.

References

[a] Rowe AK, Rowe SY, Peters DH, Holloway KA, Chalker J, Ross-Degnan D. Effectiveness of strategies to improve health-care provider practices in low-income and middle-income countries: a systematic review. Lancet Glob Health. 2018;6(11):e1163–75.

[b] Rowe SY, Peters DH, Holloway KA, Chalker J, Ross-Degnan D, Rowe AK. A systematic review of the effectiveness of strategies to improve health care provider performance in low- and middle-income countries: Methods and descriptive results. PLoS One. 2019;14(5):e0217617.

Box S2. Example of a more effective health worker (HW) performance improvement intervention (from **Table 1**, row 1)

Setting. Rural Bangladesh

Intervention objective. Improve quality of care for ill children

Intervention

- *Strengthening infrastructure:* Provision of medicines needed to implement Integrated Management of Childhood Illness (IMCI) guidelines, and establishment of a medicine tracking and reporting system.
- *Supervision:* Monthly visits that included reviewing medical records, checking availability of medicines and equipment, observing HWs during consultations, and providing feedback to HWs.
- *Other management techniques:* Establishment of a referral tracking system that involved the provision of paper referral slips to caretakers of ill children needing referral to a hospital and tracking referral completion rates.
- *Training:* 11-day in-service HW training on IMCI guidelines plus a 3-day training on how to counsel mothers on breastfeeding.

Outcomes and effect sizes. Twenty-two outcomes were assessed on patient assessment, diagnosis, treatment, and counseling. A typical outcome was "percentage of all illnesses correctly managed." For this outcome, from baseline to follow-up (23 months after intervention implementation), HW performance in the study's intervention group increased from 15.2% to 68.6% (53.4 %-point improvement), and performance in the control group increased from 7.4% to 19.2% (11.8 %-point improvement); and thus, the effect size for this outcome was 41.6 %-points (i.e., 53.4% minus 11.8%). The median effect size for all 22 outcomes was 41.8 %-points.

Source. Hoque DM, Arifeen SE, Rahman M, Chowdhury EK, Haque TM, Begum K, et al. Improving and sustaining quality of child health care through IMCI training and supervision: experience from rural Bangladesh. Health Policy Plan. 2014;29(6):753–62.

Box S3. Essential elements of a situational analysis that can inform the design of a health worker (HW) performance improvement intervention

1) Which aspects of HW performance need improvement? (What is the problem? That is, what is the gap between current and desired quality of care—e.g., based on relevant programmatic indicators from routine data, surveys, or informal observations of HW performance.) This part of the situational analysis could involve examining individual steps of the case-management process (see example by Candrinho et al.).[a]
2) What are the causes of HW performance problems? This part of the situational analysis could include facilitators and barriers to HWs performing well, use of a fishbone diagram (see example by Kumah et al.),[b] and consideration of the macro-level context (e.g., health system leadership, governance, and financing; and human resources for health issues, such as HW shortages and uneven HW distribution).
3) Are there specific health facilities or geographic areas with different subnational contexts where the performance problems are most concerning and that need to be prioritized for extra support (and thus address health inequities)?
4) What existing resources, partnerships, data, and tools could help address the problems?
5) What has already been tried to solve the problem (including any current investments)? How well did they work?
6) Are there key predictable challenges in the context that would affect intervention effectiveness (e.g., annual flooding, low access to a mobile broadband network, or seasonal migration of nomads)?
7) Which indicators of HW performance could be monitored to assess intervention effectiveness and progress on reducing the performance gap?

For frameworks for conducting a situational analysis that are used in the commercial sector in high-income countries, see Binder and Van Tiem et al.[c,d]

References

[a] Candrinho B, Plucinski MM, Colborn JM, da Silva M, Mathe G, Dimene M, et al. Quality of malaria services offered in public health facilities in three provinces of Mozambique: a cross-sectional study. Malar J. 2019;18(1):162.

[b] Kumah A, Nwogu CN, Issah AR, Obot E, Kanamitie DT, Sifa JS, et al. Cause-and-effect (fishbone) diagram: a tool for generating and organizing quality improvement ideas. Glob J Qual Saf Healthc. 2024;7:85–87.

[c] Binder C. The Six Boxes: A descendent of Gilbert's Behavior Engineering Model. Performance Improvement. 1998;37(6):48–52.

[d] Van Tiem DM, Moseley JL, Dessinger JC. Fundamentals of performance improvement: optimizing results through people, processes, and organizations. Third edition. San Francisco, USA: Pfeiffer; 2012.

Box S4. Case study of how a situational analysis was performed and used to design an intervention to improve health worker (HW) performance for tuberculosis (TB) in Kenya[a]

Essential elements of a situational analysis from ***Box S3***

1) Which aspects of HW performance need improvement (quantified)? Pediatric TB patients in Kenya were not always diagnosed. Among TB patients identified in a recent survey, 75% had visited health facilities with TB symptoms but were not diagnosed. TB program data showed only 1% of children meeting criteria for TB testing were tested (testing was available in all hospitals).

2) What are the causes of HW performance problems? Interviews with HWs and managers, observations of TB trainings and hospital practices, and document reviews revealed causes at the individual HW level (e.g., knowledge, skills, beliefs, and fears), hospital level (e.g., patient flows and resources), and system level (e.g., TB program directives and community practices and beliefs).

3) Are there health facilities or geographic areas that need to be prioritized? County and sub-county hospitals were prioritized because they have the capacity to diagnose TB.

4) What existing resources, partnerships, data, and tools could help address the problems? To illustrate the context and identify bottlenecks, the process for assessing and diagnosing patients (patient flow) at a typical county hospital was mapped. TB stakeholders were key partners.

5) What has already been tried to solve the problem and how well did they work? Distribution of TB clinical guidelines, annual HW training on TB guidelines, and procurement of TB diagnostic equipment had limited effect.

6) Are there key predictable challenges that would affect intervention effectiveness? Lack of staff.

7) Indicator to monitor HW performance? Percentage of pediatric TB cases that are diagnosed.

Methods to use the situational analysis to design an intervention to improve HW performance

- The theory-informed Behavior Change Wheel[b] framework was used to apply results of the situational analysis to specify which behaviors needed to change, link performance gaps to intervention options, and design content and feasible implementation approaches.
- Examples of linking performance gaps to interventions (from Table 1 of Oliwa et al., 2020):
  - Gap: under-detection of TB. Behavior change domains: knowledge and behavioral regulation. Interventions: training, modeling correct behaviors, and audit with feedback.
  - Gap: TB testing is done but not documented. Behavior change domain: memory attention and decision processes. Interventions: provide recording forms for documentation, and communication to stimulate action.
  - Gap: HW reluctance to diagnose TB because of stigma by caregivers. Behavior change domains: knowledge, emotion. Interventions: build HW communication skills to better counsel families, and modeling to demonstrate how best to de-stigmatize.
- The final intervention package: redesigning pediatric TB training, selection of champions, audit and feedback linked to group problem solving, and restructuring workflow with role specification.

[a] Source: Oliwa JN, et al. Improving case detection of tuberculosis in hospitalised Kenyan children—employing the behaviour change wheel to aid intervention design and implementation. Implement Sci. 2020;15:102.

[b] Michie S, van Stralen MM, West R. The behaviour change wheel: a new method for characterising and designing behaviour change interventions. Implement Sci. 2011;6:42.

Boxes S5 and S6. Best training practices based on adult learning principles

Box S5. USAID. Training methodologies and principles of adult learning for trainers of prevention of mother-to-child transmission (PMTCT) of HIV. October 2004. Accessed March 28, 2024. https://pdf.usaid.gov/pdf_docs/Pnadh003.pdf.

Section 2.3.2 Principles of adult learning (pages 16–18)

Source cited by USAID on page 18: Adapted from J. Vella. 1994. Learning to Listen, Learning to Teach.

Needs assessment: Determine what learners need to learn. Learning must address their needs and interests. Adults come to learning with personal goals and objectives, which may not be the same as those that underlie the learning situations. Find out what they expect.

1) Dialogue: Adult learning is best achieved through dialogue. Adults have enough life experience to dialogue with any teacher about any subject and will learn new attitudes or skills best in relation to that life experience. Dialogue needs to be encouraged and used in formal training, informal talks, one-on-one counseling sessions, or any situation where adults learn.
2) Safety in environment and process: Make people feel comfortable making mistakes. Adults are more receptive to learning when they are both physically and psychologically comfortable.
   - Physical surroundings (temperature, ventilation, overcrowding, light) can affect learning.
   - Learning is best when there are no distractions.
3) Respect: Appreciate learners' contributions and life experience. Adults learn best when their experience is acknowledged and new information builds on their past knowledge and experience.
4) Affirmation: Learners need to receive praise for even small attempts.
   - People need to be sure they are correctly recalling or using information they have learned.
5) Sequence and reinforcement: Start with the easiest ideas or skills and build on them. Introduce the most important ones first. Reinforce key ideas and skills repeatedly. People learn faster when information or skills are presented in a structured way.
6) Practice: Practice first in a safe place and then in a real setting.
7) Ideas, feelings, actions: Learning takes place through thinking, feeling, and doing and is most effective when it occurs across all three.
8) 20/40/80 rule: Learners remember more when visuals are used to support the verbal presentation and best when they practice the new skill. We remember 20 percent of what we hear, 40 percent of what we hear and see, and 80 percent of what we hear, see and do.
9) Relevance to previous experience: People learn faster when new information or skills are related to what they already know or can do.
   - Immediate relevance: learners should see how to use and apply what they have learned in their job or life immediately
   - Future relevance: People generally learn faster when they realise that what they're learning will be useful in the future.
10) Teamwork: Help people learn from each other and solve problems together. This makes learning easier to apply to real life.
11) Engagement: Involve learners' emotions and intellect. Adults prefer to be active participants in learning rather than passive recipients of knowledge. People learn faster when they actively process information, solve problems, or practice skills.

12) Accountability: Ensure that learners understand and know how to put into practice what they have learned.

13) Motivation: Wanting to learn
- People learn faster and more thoroughly when they want to learn. The trainer's challenge is to create conditions in which people want to learn.
- Learning is natural, as basic a function of human beings as eating or sleeping.
- Some people are more eager to learn than others, just as some are hungrier than others. In one person there are different levels of motivation.
- All the principles outlined will help the learner become motivated.

14) Clarity
- Messages should be clear.
- Words and sentence structures should be familiar. Technical words should be explained and their understanding checked.
- Messages should be visual.

15) Feedback: Feedback informs the learner in what areas s/he is strong or weak.

Box S6. Rowe AK, Rowe SY, Peters DH, Holloway KA, Ross-Degnan D. The effectiveness of training strategies to improve healthcare provider practices in low-income and middle-income countries. *BMJ Glob Health*. 2021;6:e003229. Supplemental Appendix 1, Box A. Attributes of successful training according to a specialist in the science of how people learn.

Source: Adapted from a presentation, citation: Annie Murphy Paul. "Learning Science." Presentation at the Teach to Reach Summit, Seattle, Washington, November 2, 2015.

1) The training uses analogies as bridges to link new knowledge to prior knowledge
2) Trainers actively attempt to identify and address misconceptions directly
3) Information is categorized (i.e., presented using relevant categories)
4) Information is sequenced (i.e., presented in a logical sequence)
5) Trainees practice both individual skills (e.g., performing a skin pinch to evaluate dehydration in a child with diarrhea) and the entire set of desired practices (e.g., all aspects of evaluating a child with diarrhea) (this agrees with Malcolm Knowles Principle of Andragogy #2: Experience should be at the root of all learning tasks and activities)
6) Trainers provide informational feedback (i.e., rather than only praise or criticism)
7) Complex information is simplified
8) Training uses images
9) Training uses mnemonics
10) Training uses stories, case studies, problem-based learning, or simulations (this agrees with Malcolm Knowles Principle of Andragogy #4: Adult learning should be problem-centered, rather than content-oriented)
11) Procedures are broken down into steps
12) The training has breaks to avoid overwhelming trainees
13) Trainees are asked to discuss, debate, or persuade each other
14) Some aspect of the training involves trainees collaborating with each other
15) Trainees are asked to teach each other
16) Trainers help trainees tie the training's objective to a self-relevant, self-transcendent purpose (e.g., for training on treating an illness, trainers helped trainees understand that improving treatment practices will both make them a better, more respected health worker and save the lives of people in their community) (this agrees with Malcolm Knowles Principle of Andragogy #3: Adult learning should have immediate relevance to real life)
17) Trainers recognize trainees growing competence
18) Trainers help trainees develop self-efficacy (i.e., confidence that trainees can perform the required tasks)
19) Trainers ask trainees to make a plan on how the new knowledge would be put to use. The plan includes specifying outcomes and how the outcomes will be measured, and setting goals that are short-terms, specific, and moderately challenging.
20) Trainers should get feedback from adult learners (based on Malcolm Knowles Principle of Andragogy #1: Adult learners must be involved in the design and development of their learning experience).

Text S1. Additional detail on the definition of acceptable evidence

Regarding the minimum of four study comparisons per intervention, we found that effectiveness of health worker performance improvement (HWPI) interventions in low- and middle-income countries (LMICs) based on a lower minimum (i.e., three study comparisons per intervention) led to inconsistent results when applied to data from a large systematic review on HWPI in LMICs (i.e., the Health Care Provider Performance Review, or HCPPR).[a] See **Table S2 and Box S8** in the **Online Supplementary Document**, below, for details.

The definition of acceptable evidence in this viewpoint article excludes two elements that would be ideal to have: a minimum quality of evidence,[b] and a minimum intervention implementation strength, in terms of fidelity or enactment (i.e., how completely the originally designed intervention was delivered in the field).[c,d,e] Quality of evidence was omitted because there are relatively few high-quality studies of HWPI interventions in LMICs[a] (i.e., few well done randomized-controlled trials with a low risk of bias [ROB]); and thus, most interventions do not have high quality evidence (see **Table 1** in the main article). That said, some interventions have moderate-quality evidence, and we encourage readers to consider evidence strength when interpreting the results in **Table 1**. Implementation strength was omitted because few studies from LMICs report it.[f] Note that an HCPPR analysis found no statistically significant association between intervention effectiveness and whether the intervention was fully implemented; however, this analysis was limited because many studies in the review did not report implementation strength.[a]

<u>A note of explanation on study quality and evidence quality.</u> High-quality studies and high-quality evidence are different, but related, attributes. The quality of a study means its ROB category (e.g., well done randomized-controlled trials have a low ROB and thus are high-quality studies, most well done interrupted time series have a moderate ROB and thus are moderate-quality studies, and non-randomized before-and-after studies have a high ROB and thus are low-quality studies). While ROB is an attribute of a particular study, evidence quality is an attribute of all studies that have tested the same intervention (i.e., the strength of the evidence of effectiveness for a given intervention). Evidence strength depends heavily on the ROB of all the individual studies that have tested the given intervention, but there are other factors, too (e.g., publication bias and magnitude of the effect).[b] So, one can have some high-quality (low ROB) studies, but still not have high-quality evidence. For example, an intervention might have been tested by two low ROB studies and two high ROB studies; and thus, the overall quality of the evidence for the intervention might be moderate (i.e., a kind of an average of two high-quality studies and two low-quality studies).

References

[a] Rowe AK, Rowe SY, Peters DH, Holloway KA, Chalker J, Ross-Degnan D. Effectiveness of strategies to improve health-care provider practices in low-income and middle-income countries: a systematic review. *Lancet Glob Health*. 2018;6(11):e1163–e1175.

[b] Guyatt G, Oxman AD, Akl EA, et al. GRADE guidelines: 1. Introduction—GRADE evidence profiles and summary of findings tables. *J Clin Epidemiol*. 2011; 64(4):383–94

[c] Breakthrough ACTION and PMI Impact Malaria. (2020). A Blueprint for Applying Behavioral Insights to Malaria Service Delivery: Methods and Frameworks for Improving Provider Behavior. Baltimore: Johns Hopkins Center for Communication Programs. https://breakthroughactionandresearch.org/wp-content/uploads/2020/10/Blueprint-Applying-Behavioral-Insights-Malaria-Service-Delivery.pdf. Accessed June 25, 2024.

[d] Ginsburg L, Hoben M, Berta W, et al. Development and validation of the Overall Fidelity Enactment Scale for Complex Interventions (OFES-CI). *BMJ Qual Saf*. 2024;33:98–108.

[e] Walton H. Towards comprehensive fidelity evaluations: consideration of enactment measures in quality improvement interventions. *BMJ Qual Saf*. 2024;33:78–81.

[f] Fulcher IR, Nelson AR, Tibaijuka JI, Seif SS, Lilienfeld S, Abdalla OA, et al. Improving health facility delivery rates in Zanzibar, Tanzania through a large-scale digital community health volunteer programme: a process evaluation. *Health Policy Plan*. 2020;35:1269–1279.

Text S2. Additional detail on adjusted effect sizes

In the Health Care Provider Performance Review (HCPPR), among study-specific outcomes with baseline performance measures (i.e., nearly all outcomes), effect sizes are calculated in a way that accounts for those baseline measures. For example, for outcomes expressed as a percentage, the effect size = $(\text{follow-up} - \text{baseline})_{\text{intervention}} - (\text{follow-up} - \text{baseline})_{\text{controls}}$. However, although baseline measures are utilized to calculate effect sizes, baseline measures have another potential use because intervention effectiveness and baseline performance are inversely related. For every 10 %-point decrease in baseline performance, intervention effectiveness increases by an average of 2 %-points.[a]

Between-intervention effectiveness comparisons could be biased if baseline performance is ignored. For example, if two interventions have, in truth, equal effectiveness, but the studies testing one of the interventions have lower baseline performance, then that intervention will tend to have larger effect sizes. Some analyses of the HCPPR used adjusted effect sizes to account for these differences in baseline performance.[a] These adjustments were made with a statistical model that only analyzed effect sizes of percentage outcomes. Attempts at modeling effect sizes of continuous outcomes were unsuccessful, as outliers caused model parameters to have questionable validity. In the HCPPR reanalysis presented in this report, effect sizes from percentage and continuous outcomes were included in a single integrated analysis. Thus, the use of adjusted effect sizes would have necessitated a statistical model that included both types of effect sizes. Given the difficulties modeling effect sizes of continuous outcomes, adjusted effect sizes were not used.

Table S1 in the **Online Supplementary Document** (below) presents a sensitivity analysis in which intervention effect sizes are adjusted for baseline performance. Adjusted effect size = unadjusted effect size + [(0.2) x (median baseline performance for the intervention – median baseline for all study comparisons in the table)]. This adjustment is based on the relationship between baseline and effectiveness described above. Baseline performance was estimated with results from outcomes expressed as a percentage. Here is an example of the adjustment. Row 1 of **Table S1** in the **Online Supplementary Document** presents results for the intervention "strengthening infrastructure + supervision + other management techniques + training". The unadjusted median effectiveness is 69.4 %-points, the median baseline for the studies of this intervention is 14.7%, and the median baseline for all study comparisons in the table is 41.7%. The adjusted effect size = 69.4 %-points + [(0.2) x (14.7% – 41.7%)] = 64.0 %-points. The adjusted effect size is somewhat smaller than the unadjusted effect size because the studies of the intervention had lower baselines (compared to baselines of all studies in the table), and when baselines are lower, effect sizes tend to be larger. The adjusted effect size gives an approximate indication of what the effectiveness of the intervention might have been if the studies had been done in settings with baselines that were more typical of all studies in the table (i.e., 41.7%).

Reference

[a] Rowe AK, Rowe SY, Peters DH, Holloway KA, Chalker J, Ross-Degnan D. Effectiveness of strategies to improve health-care provider practices in low-income and middle-income countries: a systematic review. *Lancet Glob Health*. 2018;6(11):e1163–e1175.

Text S3. Supportive supervision: definition and its effect in multifaceted interventions

Definition.

From page 1 of World Health Organization manual (2008, 2020).[a] "Supportive supervision is a process of helping staff to improve their own work performance continuously. It is carried out in a respectful and non-authoritarian way with a focus on using supervisory visits as an opportunity to improve knowledge and skills of health staff. Supportive supervision encourages open, two-way communication, and building team approaches that facilitate problem-solving. It focuses on monitoring performance towards goals, and using data for decision-making, and depends upon regular follow-up with staff to ensure that new tasks are being implemented correctly. Supportive supervision is helping to make things work, rather than checking to see what is wrong."

The effectiveness of supportive supervision in multifaceted interventions.

The effectiveness of supportive supervision to improve health worker (HW) performance might appear to depend on whether it is implemented as a stand-alone intervention or as part of a multifaceted intervention. The evidence suggests no such dependence. As presented in **Table 1**, the median effect of stand-alone supportive supervision is 11.2 %-points; and as shown in the bullets below, the median effect of multifaceted interventions that include supportive supervision can be greater than or less than the stand-alone value.

- Median of 69.4 %-points for the combination of strengthening infrastructure plus supportive supervision plus other management techniques plus training
- Median of 61.2 %-points for the combination of strengthening infrastructure plus supportive supervision plus training
- Median of 10.5 %-points for the combination of supportive supervision plus training
- Median of 2.7 %-points for the combination of supportive supervision plus other management techniques
- Median of 1.9 %-points for the combination of supportive supervision plus printed information or job aids for health workers

It is difficult to know whether supportive supervision functions differently as a stand-alone intervention versus part of a multifaceted intervention. Such understanding would require qualitative research on "how" the intervention components work, which rarely accompanies quantitative studies of intervention effectiveness. That said, the scientific literature does suggest that the effectiveness of supportive supervision does depend on how it is designed. As presented in the section on best practice 11, supportive supervision tends to be more effective when: 1) supervision visits include problem-solving activities with HWs or use quality improvement methods, and 2) the intervention includes supervision of supervisors.

Reference

[a] World Health Organization. Training for mid-level managers (MLM). Module 4: supportive supervision. Geneva: World Health Organization; 2008 (republished 2020). Accessed January 2, 2025. https://iris.who.int/bitstream/handle/10665/337056/9789240015692-eng.pdf

Text S4. Interpretation of quality-of-evidence categories

High quality evidence means a very good indication of the likely effect; the likelihood that the effect will be substantially different is low. Moderate quality evidence means a good indication of the likely effect; the likelihood that the effect will be substantially different is moderate. Low quality evidence means some indication of the likely effect; however, the likelihood that it will be substantially different is high. Very low quality evidence means not a reliable indication of the likely effect; the likelihood that the effect will be markedly different is very high.

Box S7. Detailed description of intervention categories in **Table 1**.

Source: Rowe AK, Rowe SY, Peters DH, Holloway KA, Chalker J, Ross-Degnan D. Effectiveness of strategies to improve health-care provider practices in low-income and middle-income countries: a systematic review. *Lancet Glob Health*. 2018;6(11):e1163–e1175. Appendix 1, pages 39–44.

Abbreviations: HW = health worker, HF = health facility

1. Community support

- HWs distributed drugs via mass community-wide campaign
- HWs distributed immunizations via mass community-wide campaign
- Cash transfers to households conditional on behaviors or use of health services
- Community member received a non-conditional cash transfer
- Community health education via group meetings (includes theater)
- Community health education via home visits
- Community health education via printed materials
- Community health education via radio
- Community health education via television
- Community health education via broadcast media
- Community health education via children exposed to the health messages outside the home
- Community health education via videos or films
- Community health education via distribution methods other than group meetings, home visits, internet, printed materials, radio, television, broadcast media, children, or videos
- Community health education via unspecified distribution methods
- Emergency telephone line was set up for consultations and to clarify when to use hospitals
- Social marketing or promotion of health goods and services
- HF-based HWs provided care in community settings (outreach)
- Community members were allowed to choose their own HWs
- Communities were given health-related supplies (e.g., bednets) that were not intended to directly impact HW performance
- Community health education via internet
- Vouchers or in-kind subsidies to households for health services or behaviors
- Communities exposed to non-health-related educational messages
- Strategy to strengthen relationships and cooperation among community members
- Communities given non-health-related resources (e.g., loan for household) that were neither intended to directly impact HW performance nor support implementation of health interventions
- Community or patient support activities (e.g., health education group meetings, home visits, printed materials) were reduced or removed
- Community members conducted group meetings to discuss health-related problems in their community and to think of possible solutions

2. Patient/client support

- Patient health education via one-on-one meetings with HWs (excludes health education during routine consultations)
- Patient health education via home visits
- Patient health education via group meetings
- Patient health education via printed materials
- Patient selected a support person

- Technology-based reminders or information for patients (patient is a passive recipient). E.g., a system that reminds a patient to take medicine at home or come to a health facility for a follow-up appointment.
- Patient health education via distribution methods other than group meetings, home visits, internet, printed materials, radio, television, broadcast media, children, videos, or technology-based system

3. Strengthening infrastructure

- Printed management information system form or register
- Health services performance reporting
- Improved data collection system (without details on how data collection was improved)
- Medical equipment inventory management or maintenance system
- Facilities repair or rebuilding
- Standard HF specifications were introduced
- Provision of drugs
- Provision of medical equipment
- Provision of facility or new service provision point (e.g., in a school)
- Non-medical equipment or supplies to support implementation of health interventions
- Structured stock ordering
- Essential drug list
- Drug therapeutic committee
- Standardized procurement systems
- At the district level, intermediary drug wholesalers were introduced
- Improved medicine logistics, including the supply chain
- Unnecessary HFs were removed (e.g., by demolishing, renting out, or changing use of HF)
- A restricted drug list was implemented at HF
- Automatic stop order
- Kit systems
- Medical technology assessment systems
- Standardized medical technology list
- Facilities maintenance
- Hiring additional HWs into pre-existing HW cadre
- HF or HW kept records of drug purchases, sales, or stock
- Introduction or revision of non-medical equipment management system

4. HW-directed financial incentives

- Payment/incentive: Performance-based financial incentives for staff
- Payment/incentive: Non-performance-based financial incentives for HW
- Payment/incentive: Salaries
- Payment/incentive: Performance-based financial incentives for HFs
- Payment/incentive: Non-performance-based financial incentives for HFs

5. Health system financing and other incentives

- The government exonerated importation taxes for essential medicines
- Private sources of revenue
- Payment/incentive: Unspecified user fees were reduced or removed
- Government funds were released for purchase of drugs
- Government funds were allocated to health sector

- Social health insurance
- Revolving drug funds
- A loan fund for HWs or HFs was set up
- A fund for patients was set up
- Payment/incentive: Patients receiving medicines were charged the cost of the drug packaging
- Payment/incentive: Consultation fee was introduced or increased
- Payment/incentive: Consultation fee was reduced or removed
- Payment/incentive: Fee per drug/health commodity item was introduced or increased
- Payment/incentive: Fee per drug/health commodity item was reduced or removed
- Payment/incentive: Prescription fee was introduced or increased
- Payment/incentive: Fee per service was introduced or increased
- Payment/incentive: Fee per service was reduced or removed
- Payment/incentive: Performance-based non-financial incentives for staff
- Payment/incentive: Non-performance-based non-financial incentives for HW
- Retail prices of essential medicines and consumables were set by government
- HF received recognition after meeting certain criteria
- HF owners were given incentives
- Contracting-in services
- Contracting-out services
- Other contracting
- Foreign countries/multinational org/bilateral organization (e.g., World Bank) donated funds to support strategies
- Funds were donated to support strategies, source unspecified
- Different insurers were unified into a single insurer
- Contracting with incentives at contractor's level
- Donations as a source of revenue for health
- Insurance premiums
- Taxes as a source of revenue for health
- Community-based health insurance
- Enterprise-based health insurance
- Private/voluntary health insurance
- Personal health savings
- Capitation fee
- Global budgets to HFs or organizations
- Payment/incentive: Unspecified user fees were introduced or increased
- Reimbursement (e.g., by insurance) or subsidy to HF or HW was revised
- Promotion of brand name competitor drug
- Pharmaceutical promotion activities that do not involve reducing drug price
- Reduction in price of a brand name competitor drug
- Reduction in price of a generic competitor drug
- Reduction in price of a drug named in study outcome
- Managers of HWs received performance-based incentives

6. Regulation and governance

- Standard drug quality requirements were introduced
- Franchising or branding private HWs
- Enforcement approach
- Accreditation

- Certification
- Licensing
- Registration
- Redress mechanism
- Sanctions based on HW qualifications or facility structural factor
- Sanctions based on services or negligence of HW
- A code of ethics for HWs or HFs was created
- Public-private partnerships not involving contracting
- Resource control for health services given to civil society organizations
- Resource control for health services given to local governments
- Civil society organization oversight of HWs
- HFs were now in control of fee revenues
- Responsibility or authority for HW decisions to local governments
- Responsibility or authority for HW decisions to local health agencies
- Resource control for health services given to local public health agencies
- Community scorecards and community reporting
- Patient ratings of HWs that are not community scorecards
- Patient bill of rights
- Banning drug or formulation
- Approval of a brand name competitor drug by committee at HF level or higher
- Approval of a generic competitor drug by committee at HF level or higher
- Approval of a drug named in study outcome by committee at HF level or higher
- Community members and HWs made a formal agreement on the responsibilities of HWs who serve the communities

7. Group problem solving

- Continuous quality improvement (an ongoing cycle in which performance is monitored, problems are identified, and interventions to correct the problems are implemented)
- Collaborative improvement (a group of health facilities work together to solve a specific problem using teamwork, plan-do-study-act cycles, coaching, and learning sessions)
- HWs held meetings to discuss problems and solutions (but not formal teams)
- Team-based problem solving

8. Supervision

- Benchmarking
- Supervision
- HWs sought second opinion from peer or higher level HW
- HW received instructions from higher level HW
- Managers of HWs received supervision
- Managers of HWs received training
- HW received support from non-supervisory staff
- Audit with in-person feedback
- Audit with written feedback
- Monitoring of HW practice parameters
- Peer review
- HFs were inspected to monitor for deviations from regulations
- Drug utilization review/evaluation
- Performance appraisal practices

9. Other management techniques (i.e., not group problem solving or supervision)

- Risk management
- HW group process that is neither group training nor team-based problem solving
- Group meeting of HWs and community members or patients
- HWs were given job descriptions
- HW self-assessment
- Each HF set its own monthly performance targets
- HWs participated in unspecified group meeting
- Reorganized management structure
- Hiring and selection process
- One or more new health services was integrated into the regular activities of HFs
- HF and HWs were linked in a referral network
- Structured prescribing
- Change in process of care to improve accessibility of medicines
- Generic substitution
- Pre-packaging drugs
- Change in process of care to improve utilization of health services (including pharmaceuticals and follow-up care)
- Management of HWs was decentralized to HF level (e.g., management committee was created at HF that gave staff more decision-making rights regarding management of HF)
- HWs received training on management skills (business planning, record-keeping, financial reporting, credit management, marketing)
- Prior authorization of pharmaceuticals
- Operations research
- Regular monitoring on parameters not related to HW clinical practice (e.g., HW knowledge, patient outcomes)
- Reorganization of how existing HWs are deployed (e.g., Higher-level-HF-based HWs provided care in lower-level HFs)
- HWs received feedback that was not based on data collected from an audit or supervisory visit
- Restrictions for prescribing a brand name competitor drug
- Restrictions on making referrals
- Disciplinary action within HW organization
- Personnel development practices
- Promotion practices
- Managers of HWs received feedback
- Managers of HWs participated in group meetings

10. HW training

- In-service training
- Academic detailing (i.e., one-on-one training by an opinion leader)
- Pre-service training
- Informal education of HWs by their peers (peer-to-peer learning)

11. Printed information or job aids for HWs that is not an integral part of another component

- Printed pamphlet for HW (excludes standard treatment guidelines)
- Printed patient recording form
- Printed standard treatment guidelines

- Poster for HW
- Other printed job aid for HW (e.g., counseling cards for nurses, map of referral centers)
- Label or stamp that HW uses to depict dosing
- Printed newsletter for HW
- Printed literature for HWs (not considered a job aid and not part of distinct self-study intervention)
- Bulletin for HW
- Formulary manual
- Printed educational materials for HWs to study on their own (i.e., a distinct self-study intervention)

12. Information and communication technology (includes mHealth and eHealth) for HWs

- Electronic copies of literature for HWs (not a job aid, not actively sent to HWs, and not part of a self-study intervention)
- Computerized decision aid (i.e., not simply a generic reminder sent to all HWs)
- Data were collected in new or modified electronic management information system and used to improve services
- Electronic medical record system that is not a decision aid
- Reminders or information sent to HWs (HW is passive recipient), e.g., SMS text message reminders to all HW phones
- Facilitates communication with a HW peer or higher-cadre HW (e.g., a supervisor), e.g., a mobile phone-based system that a HW can use to ask for help with a patient, facilitate a referral for a patient, or ask for more drugs or supplies
- Facilitates communication with between HWs and patients. For example, a mobile phone-based system that a HW can use to actively answer patient question or remind a patient of follow-up appointment.
- Electronic educational materials for HWs to study on their own (e.g., via cell phone or web-based) that is a distinct self-study intervention

Table S1. Effectiveness of interventions to improve performance of health workers in low- and middle-income countries, based on acceptable evidence from a large systematic review: sensitivity analysis in which effectiveness is adjusted for baseline performance

Intervention (see **Box S7** in the **Online Supplementary Document** for definitions of component categories)	Analysis of all studies (all risk of bias levels)					
	No. of study comparisons	Unadjusted median effectiveness, in percentage-points (interquartile range)	Quality of evidence*	Median performance at baseline (%)	Percent of all outcomes with a baseline value that could be used for the adjustment	Adjusted median effectiveness, in percentage points†
Interventions for professional health workers (e.g., physicians, nurses, and midwives)						
Interventions with median effectiveness greater than the threshold of 15 percentage-points						
Strengthening infrastructure plus supervision‡ plus other management techniques plus training	5	69.4 (47.5 to 297.1)	Moderate	14.7	75.0% (21/28)	64.0
Group problem solving plus training	4	62.4 (47.0 to 77.8)	Moderate	25.2	83.3% (10/12)	59.1
Strengthening infrastructure plus supervision plus training	7	61.2 (8.0 to 77.0)	Moderate	31.2	60.0% (6/10)	59.1
Group problem solving only	15	27.2 (8.1 to 51.7)	Low	26.1	81.1% (30/37)	24.1
Community support plus supervision plus training	4	19.6 (8.9 to 24.1)	Low	36.5	100.0% (14/14)	18.5
Patient/client support plus training	6	18.0 (2.8 to 23.1)	Low	28.9	94.3% (50/53)	15.4
Interventions with median effectiveness less than or equal to the threshold of 15 percentage-points						
Supervision plus other management techniques plus training	6	12.6 (0.6 to 31.0)	Low	8.4	76.9% (10/13)	5.9
Other management techniques plus training	6	11.9 (9.9 to 21.3)	Low	27.0	85.7% (12/14)	8.9
Health system financing (e.g., health insurance or removal of user fees)	4	11.9 (–8.3 to 46.4)	Low	26.6	36.4% (4/11)	8.8
Supervision only	20	11.2 (4.8 to 21.4)	Moderate	52.9	89.5% (51/57)	13.4
Supervision plus training	27	10.5 (5.0 to 35.3)	Very low	34.2	90.2% (110/122)	9.0
Other management techniques only	4	10.4 (0.7 to 22.0)	Moderate	61.5	100.0% (9/9)	14.4
Training only	78	9.7 (3.0 to 20.3)	Low	40.3	94.4% (456/483)	9.4

Strengthening infrastructure only	4	7.1 (–3.5 to 155.5)	Moderate	84.0	71.4% (5/7)	15.5
Information and communication technology (i.e., digital interventions) for health workers only§	11	6.5 (–2.4 to 15.8)	High	51.2	96.7% (29/30)	8.4
Community support <u>plus</u> training	5	6.1 (4.5 to 10.2)	Low	57.7	77.8% (7/9)	9.3
Supervision <u>plus</u> other management techniques	5	2.7 (1.6 to 5.8)	Very low	76.6	66.7% (10/15)	9.6
Supervision <u>plus</u> printed information or job aids for health workers	5	1.9 (–0.6 to 16.7)	Low	50.3	90.7% (49/54)	3.6
Performance-based financing§	16	1.8 (–0.7 to 5.2)	Moderate	56.2	88.3% (121/137)	4.6
Printed information or job aids for health workers only	8	1.6 (–0.4 to 6.1)	Moderate	35.3	84.0% (21/25)	0.3
Interventions for lay health workers (e.g., community health workers)						
Interventions with median effectiveness <u>less</u> <u>than</u> <u>or</u> <u>equal</u> <u>to</u> the threshold of 15 percentage-points (note that no intervention had a median effectiveness greater than 15 percentage points based on acceptable evidence, as defined in this report)						
Information and communication technology (i.e., digital interventions) for health workers only§	13	6.7 (2.9 to 12.4)	High	63.3	90.0% (18/20)	11.0
Training only	4	2.4 (–1.1 to 7.4)	Very low	70.0	100.0% (15/15)	8.0

* Based on the Grading of Recommendations Assessment, Development, and Evaluation (GRADE) system. See **Text S4** in the **Online Supplementary Document**.

† Adjusted effect size = unadjusted effect size + [(0.2) x (median baseline performance for the intervention – median baseline for all study comparisons in the table)]. The median baseline for all study comparisons in the table is 41.7%. See **Text S2** in the **Online Supplementary Document**, for details.

‡ Supervision refers to supportive supervision and other similar interventions (e.g., audit with feedback); and training refers to any learning strategy, such as group in-service training, educational outreach, or peer-to-peer training.

§ Results for this intervention come from a rapid review (see **Text S6** in the **Online Supplementary Document** for performance-based financing, and **Text S8** in the **Online Supplementary Document** for information and communication technology).

Table S2. Effectiveness of interventions to improve health worker performance in low- and middle-income countries, based on acceptable evidence from a large systematic review (see **Box S1** in the **Online Supplementary Document**): interventions tested by at least three study comparisons. Interventions tested by exactly three study comparisons are in bold. Inconsistencies are presented in **Box S8** in the **Online Supplementary Document**, below.

Intervention (see **Box S7** in the **Online Supplementary Document** for definitions of component categories)	Analysis of all studies (all risk of bias levels)			Analysis of higher quality studies (i.e., low or moderate risk of bias)*		
	No. of study comparisons	Median effectiveness, in percentage-points (interquartile range)	Quality of evidence†	No. of study comparisons	Median effectiveness, in percentage-points (interquartile range)	Quality of evidence
Interventions for professional health workers (e.g., physicians, nurses, and midwives)						
Interventions with median effectiveness greater than threshold of 15 percentage points						
**Community support plus strengthening infrastructure plus supervision‡ plus other management techniques plus training**	**3**	**76.1 (NA)**	**NA**	-	-	-
Strengthening infrastructure plus supervision plus other management techniques plus training	5	69.4 (47.5 to 297.1)	Moderate	-	-	-
**Strengthening infrastructure plus health system financing and other incentives plus supervision plus other management techniques plus training**	**3**	**63.0 (NA)**	**NA**	-	-	-
Group problem solving plus training	4	62.4 (47.0 to 77.8)	Moderate	-	-	-
Strengthening infrastructure plus supervision plus training	7	61.2 (8.0 to 77.0)	Moderate	4	64.3 (32.1 to 88.7)	High
**Health worker-directed financial incentives only**	**3**	**42.0 (NA)**	**NA**	-	-	-
Group problem solving only	15	27.2 (8.1 to 51.7)	Low	-	-	-
**Group problem solving plus supervision plus training**	**3**	**21.1 (NA)**	**NA**	-	-	-
**Strengthening infrastructure plus supervision**	**3**	**19.7 (NA)**	**NA**	-	-	-
Community support plus supervision plus training	4	19.6 (8.9 to 24.1)	Low	-	-	-
Patient/client support plus training	6	17.9 (2.8 to 23.1)	Low	-	-	-
**Other management techniques plus printed information or job aids for health workers**	**3**	**16.5 (NA)**	**NA**	**3**	**16.5 (NA)**	**NA**

Interventions with median effectiveness less than or equal to threshold of 15 percentage points						
Supervision plus other management techniques plus training	6	12.6 (0.6 to 31.0)	Low	-	-	-
Other management techniques plus training	6	11.8 (9.9 to 21.3)	Low	-	-	-
Health system financing and other incentives only	4	11.8 (–8.3 to 46.4)	Low	-	-	-
Supervision only	20	11.2 (4.8 to 21.4)	Moderate	11	11.7 (2.4 to 27.9)	High
Supervision plus training	27	10.5 (5.0 to 35.3)	Very low	11	9.2 (4.5 to 30.0)	Low
Other management techniques only	4	10.4 (0.7 to 22.0)	Moderate	-	-	-
Training only	79	9.7 (3.0 to 20.5)	Low	33	9.0 (2.7 to 19.8)	Moderate
Strengthening infrastructure only	4	7.1 (–3.5 to 155.5)	Moderate	4	7.1 (–3.5 to 155.5)	Moderate
Information and communication technology (i.e., digital interventions) for health workers only§	11	6.5 (–2.4 to 15.8)	High	11	6.5 (–2.4 to 15.8)	High
Community support plus training	5	6.1 (4.5 to 10.2)	Low	-	-	-
**Strengthening infrastructure plus supervision plus training plus information and communication technology (i.e., digital interventions) for health workers**	**3**	**3.9 (NA)**	**NA**	-	-	-
Supervision plus other management techniques	5	2.7 (1.6 to 5.8)	Very low	-	-	-
Supervision plus printed information or job aids for health workers	5	1.9 (–0.6 to 16.7)	Low	-	-	-
Performance-based financing§	16	1.8 (–0.7 to 5.2)	Moderate	11	0.6 (–1.0 to 3.9)	Moderate
Printed information or job aids for health workers only	8	1.6 (–0.4 to 6.1)	Moderate	5	2.5 (0.8 to 5.2)	High
**Group problem solving plus information and communication technology (i.e., digital interventions) for health workers**	**3**	**–2.3 (NA)**	**NA**	**3**	**–2.3 (NA)**	**NA**

Interventions for lay health workers (e.g., community health workers)						
Interventions with median effectiveness less than or equal to the threshold of 15 percentage-points (note that no intervention had a median effectiveness greater than 15 percentage points based on acceptable evidence, as defined in this report)						
Information and communication technology (i.e., digital interventions) for health workers only§	13	6.7 (2.9 to 12.4)	High	13	6.7 (2.9 to 12.4)	High
Training only	4	2.4 (–1.1 to 7.4)	Very low	-	-	-

NA = Not applicable or not calculated

* Blank row means the intervention was tested by fewer than three study comparisons with a low or moderate risk of bias.

† Based on the Grading of Recommendations Assessment, Development, and Evaluation (GRADE) system (Guyatt et al. 2011) (see **Text S4** in the **Online Supplementary Document**).

‡ Supervision refers to supportive supervision and other similar interventions (e.g., audit with feedback); and training refers to any learning strategy, such as group in-service training, educational outreach, or peer-to-peer training.

§ Results for this intervention come from a rapid review (see **Text S6** in the **Online Supplementary Document** for performance-based financing, and **Text S8** in the **Online Supplementary Document** for information and communication technology).

Box S8. Examples of inconsistent results on intervention effectiveness (from **Table S2** in the **Online Supplementary Document**, above) for interventions tested with three study comparisons

- Group problem solving only has a median of 27.2 %-points (from 15 study comparisons), but group problem solving plus information and communication technology (i.e., digital interventions) for health workers has a median of –2.3 %-points (from 3 study comparisons).
- Strengthening infrastructure plus supervision plus training has a median of 61.2 %-points (from 7 study comparisons), but strengthening infrastructure plus supervision plus training plus information and communication technology (i.e., digital interventions) for health workers has a median of 3.9 %-points (based on 3 study comparisons).
- Group problem solving plus training has a median of 62.4 %-points (from 4 study comparisons), but group problem solving plus training plus supervision has a median of 21.1 %-points (from 3 study comparisons).

Section 2. Checklist tools for assessing health worker performance improvement (HWPI) investments

Checklist S1. Long version of the tool for assessing HWPI investments

Introduction. The purpose of this tool is to help users (e.g., country staff and their partners who develop HWPI investments and donor staff who review grant applications) assess whether an HWPI investment is based on acceptable evidence and best practices and thus is likely to cause improvements in health worker (HW) performance and quality of care that are larger than what would be expected from typical HWPI investments, such as stand-alone HW training. If an investment is not aligned with the best practices, then the tool shows the limitations, which could be opportunities to improve the investment. The overarching goal is to optimize intervention effectiveness, and thus improve HW performance and save lives. The tool is for assessing an investment to address a programmatic issue in a specific context (e.g., improving immunization services in a particular country). Below, the term "intervention" refers to an intervention package that a country wants to use, which could include one intervention component (e.g., a new digital tool that a country wants to evaluate) or multiple components (e.g., training plus group problem-solving).

Step 1. Assess the proposed HWPI intervention and check the box for each of the following best practices.

The intervention:

1. Has an expected median or mean effectiveness that (choose option 1.1, 1.2, or 1.3):
   1.1. [ ] Is greater than 15 percentage-points, based on acceptable evidence (as defined in this viewpoint article)
   1.2. [ ] Is less than or equal to 15 percentage-points, based on acceptable evidence
   1.3. [ ] Has not been evaluated with acceptable evidence (a "needs more evidence" intervention)
2. [ ] Is based on a situational analysis
3. [ ] Is clearly described and has measurable objectives
4. [ ] Documents evidence of country ownership
5. [ ] Involved HW input
6. [ ] Builds on existing HWPI-relevant infrastructure and policies
7. [ ] Demonstrates country capacity to implement the intervention or a plan to develop the capacity
8. [ ] Has a realistic budget
9. [ ] Includes a plan for scale-up and sustainability
10. [ ] *If the intervention includes HW training*: a) at least part of the training is done where HWs routinely work, b) the intervention includes practice of the skills taught in the training, c) the intervention includes "low-dose, high-frequency" training*, and d) the intervention uses competency-based training and other adult learning principles
11. [ ] *If the intervention includes supervision:* a) supervision visits include problem-solving activities with HWs or use quality improvement methods, and b) the intervention includes supervision of supervisors
12. [ ] Includes a system to monitor HW performance, assess intervention effect, and act on the information
13. [ ] Will be implemented in tandem with specific actions to address broader health workforce issues

* An educational activity with at least two short training sessions (i.e., lasting a few minutes up to 4 days) that are spaced at least one week apart, that occur at HWs' worksite, and that include practicing clinical skills (simulations or treating real patients).

Step 2. Based on the results of Step 1, categorize the effectiveness of the proposed HWPI intervention and check the appropriate box.

The intervention:

14.1. [ ] Is more effective (i.e., item 1.1 is checked **and** items 2–13 are all checked**)
14.2. [ ] Is less effective (i.e., item 1.2 is checked **or** any items 2–13 are not checked**)
14.3. [ ] Needs more evidence (i.e., item 1.3 is checked)

Step 3. Based on the results of Steps 1 and 2, assess the proposed HWPI intervention and check the box for each of the following attributes.

If the intervention needs more evidence (i.e., item 1.3 is checked):
15. [ ] The intervention will be implemented in the context of a study to evaluate its effectiveness **and** the study will generate acceptable evidence

If the intervention is less effective (i.e., item 14.2 is checked):
16. [ ] There is a compelling reason for its use that includes documented effectiveness greater than 15 %-points in the context where it will be used

Step 4. Based on the results of Steps 1–3, determine if the proposed HWPI investment is aligned with best practices and check the appropriate box.

The investment:

[ ] Is aligned with best practices—i.e., **any one** of the following criteria:
- Item 14.1 is checked (a more effective intervention)
- Items 1.3, 2–13, and 15 are all checked** (a "needs more evidence" intervention that will be evaluated)
- Items 1.2, 2–13, and 16 are all checked (a less effective intervention with documented effectiveness > 15 %-points *in the context where it will be used*)

[ ] Is not aligned with best practices—i.e., none of the above three criteria is met

*** Items 10 and 11 are only applicable if the intervention includes training or supervision, respectively.*

Step 5. Interpret the results. If the HWPI investment is missing any of the best practices, then consider why. Could the investment design be revised (in ways that are feasible and acceptable to the country) to use all applicable best practices?

Checklist S2. Short version of the tool for assessing HWPI investments

Introduction. The purpose of this tool is to help users (e.g., country staff and their partners who develop HWPI investments and donor staff who review grant applications) assess whether an HWPI investment is based on acceptable evidence and best practices and thus is likely to cause improvements in health worker (HW) performance and quality of care that are larger than what would be expected from typical HWPI investments, such as stand-alone HW training. If an investment is not aligned with the best practices, then the tool shows the limitations, which could be opportunities to improve the investment. The overarching goal is to optimize intervention effectiveness, and thus improve HW performance and save lives. The tool is for assessing an investment to address a programmatic issue in a specific context (e.g., improving immunization services in a particular country). Below, the term "intervention" refers to an intervention package that a country wants to use, which could include one intervention component (e.g., a new digital tool that a country wants to evaluate) or multiple components (e.g., training plus group problem-solving).

Step 1. Assess the proposed HWPI intervention and check the box for each of the following best practices.

The intervention:

1. [ ] Has an expected median or mean effectiveness that is greater than 15 percentage-points*
2. [ ] Is based on a situational analysis
3. [ ] Is clearly described and has measurable objectives
4. [ ] Documents evidence of country ownership
5. [ ] Involved HW input
6. [ ] Builds on existing HWPI-relevant infrastructure and policies
7. [ ] Demonstrates country capacity to implement the intervention or a plan to develop the capacity
8. [ ] Has a realistic budget
9. [ ] Includes a plan for scale-up and sustainability
10. [ ] *If the intervention includes HW training*: a) at least part of the training is done where HWs routinely work, b) the intervention includes practice of the skills taught in the training, c) the intervention includes "low-dose, high-frequency" training**, and d) the intervention uses competency-based training and other adult learning principles
11. [ ] *If the intervention includes supervision:* a) supervision visits include problem-solving activities with HWs or use quality improvement methods, and b) the intervention includes supervision of supervisors
12. [ ] Includes a system to monitor HW performance, assess intervention effect, and act on the information
13. [ ] Will be implemented in tandem with specific actions to address broader health workforce issues

Step 2. Interpret the results.

If the proposed HWPI investment is missing any of the best practices, then consider why. Could the investment design be revised (in ways that are feasible and acceptable to the country) to use all applicable best practices?

* For example, an effectiveness of 25 percentage-points means that HW performance (such as for an outcome like: percentage of clients correctly counseled) is 25 percentage-points higher after the intervention is implemented—e.g., an improvement from 50% performance at baseline to 75% after the intervention.

** An educational activity with at least two short training sessions (i.e., lasting a few minutes up to 4 days) that are spaced at least one week apart, that occur at HWs' worksite, and that include practicing clinical skills (simulations or treating real patients).

Section 3. Research priorities related to health worker (HW) performance improvement interventions

Text S5. List of research priorities

1) **For technology-based interventions:** What are the criteria for assessing which contexts can support technology-based interventions (potentially related to the assessment of intervention maturity[a])? What is the effectiveness of artificial intelligence (AI)-enabled decision-support tools and adaptive learning systems?

2) **For training:** What is the effectiveness of eLearning[b] and blended learning, compared to only in-person training, in terms of its effect on HW performance? What are the benefits of eLearning and blended learning, in terms of cost and feasibility, if any? Are there some contexts in which eLearning is less effective than in-person training and should be avoided? How effective are strategies linking training interventions with continuous professional development opportunities (e.g. training curriculum content that is standardized and approved by national training institutions)? What are examples of effective training that are different from the usual courses focused on improving HW performance on delivering clinical services (e.g., Field Epidemiology Training Programs[c], which is designed to strengthen public health capacity)?

3) **For supervision and mentoring:** Are digitally-supported supervision interventions as effective as in-person supervision, in terms of its effect on HW performance? If yes, what are the cost savings, if any? How do digital-only supervision interventions compare in effectiveness to a blended approach that combines digitally-supported and in-person supervision? Are there some contexts in which digital supervision is less effective than in-person supervision and should be avoided? What are the best ways to embed group problem solving into routine supervision? How effective is mentoring to improve HW performance? How does digital mentoring versus in-person mentoring compare in effectiveness? Which specific mentoring practices are most important? How effective are "communities of practice" to improve HW performance (including the Extension for Community Healthcare Outcome [ECHO][d] model)?

4) **For leadership and management:** What is the effect (on HW performance) of interventions to strengthen leadership and management? What is the effect of interventions that create "enabling environments"[e] for HWs? Which aspects of an enabling environment are most important? An enabling environment might include elements such as HWs feeling: a) motivated, b) appreciated, c) that decisions are made collaboratively, d) high levels of trust and mutual respect among team members, and e) satisfied with their autonomy to make decisions at work.

5) **For community HWs:** More research on intervention effectiveness is needed to replicate findings from interventions that have already been tested (with fewer than four study comparisons) and to characterize the effectiveness of novel interventions.

6) **Cost:** More research is needed on intervention cost, cost-effectiveness, and other relevant economic outcomes.

7) **Understanding heterogeneity:** For any given intervention type (e.g., training, supervision, digitally-supported interventions, etc.), the effectiveness on HW performance from individual studies varies widely. What are the causes of this heterogeneity? I.e., why is an intervention highly effective in one study but ineffective in another? Is it differences in the research methodologies of the studies? Is it the method for "matching" the intervention to the problem that needs to be solved? Is it the fidelity of the intervention implementation? Which contextual factors affect intervention effectiveness?[f]

8) **Updating the evidence base:** Systematic reviews are needed to gather and synthesize information on the effectiveness of interventions to improve HW performance, with a focus on the literature after 2016 (when the literature search was done for the Health Care Provider Performance Review, which is used extensively in this report).[g] These reviews may result in changing or disaggregating intervention categories based on new literature (see **Box S7** in the **Online Supplementary Document** for the categories used in this report). Systematic reviews should cover actions that address broader health workforce challenges—i.e., on how HW performance is impacted by interventions and policies that affect HW stocks (including numbers contracted by partners to staff facilities, programs, etc.), the equitable distribution of HWs, provider payment (including partner-supported financial incentives, top-ups, etc.), skills mix interventions (including task sharing, upskilling, other optimization, which are often supported by partners), and other areas. To help address health inequities, systematic reviews should identify which HWPI interventions are particularly effective for populations that are especially affected by inadequate quality of health care.

[a] World Health Organization. Monitoring and evaluating digital health interventions: a practical guide to conducting research and assessment. Geneva: World Health Organization; 2016. Accessed August 14, 2023. https://www.who.int/publications/i/item/9789241511766

[b] Education delivered through digital technologies.

[c] For example, see: Al Nsour M, Khasawneh G, Khader Y, Bashier H. Evaluation of field epidemiology training programs: a scoping review. *Front Epidemiol*. 2024;4:1376071.

[d] For example, see: Fokom Domgue J, Dille I, Fry L, Mafoma R, Bouchard C, Ngom D, et al. Enhancing cervical and breast cancer training in Africa with e-learning. *Lancet Glob Health*. 2023;11(1):e28–e29.

[e] Source: FHI 360 webinar, "How can health systems meaningfully enable behavior change and performance of health system actors?", April 17, 2024.

[f] For example, see Table N1 and N2 in Appendix 1 of: Rowe AK, Rowe SY, Peters DH, Holloway KA, Chalker J, Ross-Degnan D. Effectiveness of strategies to improve health-care provider practices in low-income and middle-income countries: a systematic review. *Lancet Glob Health*. 2018;6(11):e1163–e1175.

[g] Rowe SY, Peters DH, Holloway KA, Chalker J, Ross-Degnan D, Rowe AK. A systematic review of the effectiveness of strategies to improve health care provider performance in low- and middle-income countries: Methods and descriptive results. *PLoS One.* 2019;14(5):e0217617.

Section 4. Supplementary rapid reviews

Text S6. Performance-based financing

A rapid review of performance-based financing to improve healthcare provider practices in low- and middle-income countries

OBJECTIVE

Characterize the effect of performance-based financing (PBF) on healthcare provider (HCP) practices in low- and middle-income countries (LMICs).

METHODS

- Definition of PBF. PBF involves payment to health facilities for the quantity and/or quality of health services provided. PBF usually includes other intervention components, such as monitoring of health services (which is used to determine payments), autonomy for health facility staff so that they can determine how to spend the payments (which usually includes payments to HCPs), training on financial management, and supervision.

- Definition of HCP. Health workers that provide health care to patients or that perform other health-related activities (e.g., educating patients or community members) in a hospital, outpatient clinic, or community setting.

- Inclusion criteria. Eligible studies: 1) were done in an LMIC; 2) examined the quantitative effect of PBF on HCP practices (or processes of care), such as correct patient assessment, diagnosis, treatment, counseling, referral, preventive services, and documentation; and 3) used an eligible study design. Eligible study designs were: 1) controlled before-and-after study (with or without randomization), 2) post-intervention-only study with randomized controls, or 3) interrupted time series. Eligible effect sizes were based on a comparison of a PBF intervention arm and a no-intervention control arm.

- Exclusion criteria. 1) Outcomes of "inputs" or "structural quality" (e.g., availability of medicines), 2) HCP knowledge outcomes, 3) outcomes of community-level coverage that included all community members regardless of whether they had sought care from HCPs (e.g., proportion of all newborns that received polio vaccination, as measured by a household survey), and 4) effect sizes based on a comparison of two active interventions (e.g., a PBF intervention arm compared to a direct facility financing arm). Outcomes of inputs and HCP knowledge were excluded because they are only weakly correlated with HCP practices [Kruk et al. 2018; Rowe et al. 2017]. Outcomes of community coverage that included all community members regardless of whether they had sought care from HCPs were excluded because achieving these outcomes requires a combination of care-seeking by community members and correct HCP practices.

- Literature search. The primary source of articles was the most recent PBF review by The World Bank [de Walque et al. 2022]. Studies for this World Bank review were identified by "several comprehensive literature searches between September 2016 and March 2021" [de

Walque et al. 2022, page 139]. For the rapid review presented here, there were three other components of the literature search. First, the World Bank review mentioned a PBF program in Haiti, but no citation was provided; thus, bibliographies of other PBF studies were hand-searched to identify the Haiti study [Zeng et al. 2013]. Second, the published report of a previously known, relatively recent PBF study in the Democratic Republic of the Congo that did not appear in the World Bank review was identified [Shapira et al. 2023]. Third, a search on PubMed was performed on December 2, 2024, using the search terms "performance-based financing" AND "developing countries" AND "trial" (limited to articles from January 2022 to December 2024, which covered the period from the 2022 World Bank review to the present). This search yielded 321 citations, but no new PBF studies.

- Data abstraction. Although the World Bank review was the source for identifying PBF studies, the review did not present effect sizes for all studies with the methodology used in this rapid review (see analysis methods, below). Therefore, effect sizes (or outcome values needed to calculate effect sizes) from eligible outcomes were abstracted from the original PBF study reports, which are listed in the tables below. Effect sizes were abstracted by one person (AKR). For one study (of the PBF program in Rwanda that began in 2002), the data had already been abstracted and effect sizes calculated by a large systematic review (the Health Care Provider Performance Review, or HCPPR] [Rowe et al. 2019]); and thus, the effect size from this systematic review was used.

- Risk of bias (ROB) assessment. For most studies, the source of ROB assessments was the World Bank review, which categorized risk of bias as low, medium, or high [de Walque et al. 2022; Table 6A.1]. For the one Rwandan study for which results were obtained from the HCPPR, the ROB from the HCPPR was used. The HCPPR ROB categories are low, moderate, high, and very high. (Moderate and medium ROB were considered equivalent.) For all other studies, the ROB was largely based on the study design—i.e., low risk for randomized-controlled trials (RCTs), moderate risk for interrupted time series studies and RCTs with limitations, and high risk for non-randomized controlled before-and-after studies.

- Analysis. Effect sizes were measured in terms of absolute percentage-point (%-pt) improvements. For outcomes measured as a percentage that could plausibly change over the entire range from 0 to 100% (i.e., most HCP practice outcomes, such as percentage of children weighed), the effect size = (follow-up – baseline)$_{\text{intervention}}$ – (follow-up – baseline)$_{\text{controls}}$. For continuous unbounded outcomes and for percentage outcomes that are only expected to change over a narrow range (e.g., neonatal mortality before 24 hours), the effect size = 100% x {[(follow-up – baseline)/baseline]$_{\text{intervention}}$ – [(follow-up – baseline)/baseline]$_{\text{controls}}$}. Often, PBF studies did not present effect sizes calculated this way, and the reports did not include baseline and follow-up outcome values that could be used to calculate effect sizes. Instead, PBF studies reported similar kinds of effect sizes (usually described as difference-in-differences effects) that were estimated with statistical regression models that adjusted for potential confounders. When available, these model-based effect size estimates were used. For each PBF study comparison, the median of effect sizes (MES) from eligible outcomes was calculated. The distribution of the study-comparison-specific MES values was described with a median and interquartile range. The main analysis included

studies with all ROB categories, and a sensitivity analysis only included studies with low or moderate ROB.

- Supplemental information from study investigators. When there were questions about measurement methodology or the interpretation of results for a particular PBF study, an attempt was made to contact the study investigators for information. These contacts are listed as personal communications in the tables and reference list below.

RESULTS

Sixteen study comparisons (and thus 16 MES values) were included from 15 countries. One country (Rwanda) had a study with two PBF intervention arms (**Table S3** in the **Online Supplementary Document**). Nine studies from seven countries were excluded (details available on request). Among the 16 included study comparisons, two had a low ROB, nine had a medium (i.e., moderate) ROB, three had a high ROB, and two had a very high ROB. Overall, the quality of evidence was moderate. For the main analysis (with all ROB categories), among the 16 study comparisons, the median MES was 1.8 %-pts (interquartile range: –0.7 to 5.2 %-pts). For the sensitivity analysis (with only low or moderate ROB levels), among the 11 study comparisons, the median MES was 0.6 %-pts (interquartile range: –1.0 to 3.9 %-pts).

Table S3. The effect of performance-based financing (PBF) on healthcare provider (HCP) practice outcomes in low- and middle-income countries

Country	References	Methods and risk of bias (ROB)	Results of PBF vs. true controls<br>(C = control arm, ES = effect size, I = intervention arm)	Median effect size (PBF vs. controls)	Comments
Afghanistan	Engineer et al. 2016	RCT vs. true controls. Intervention was PBF. No other components.<br>ROB: Medium	**Table 4 had 4 HCP practice outcomes.**<br>Client background and physical assessment index (I: 76.4%, C: 72.3%, ES: 4.1 %-pts); client counselling index (I: 35.3%, C: 29.3%, ES: 6.0%-pts); universal precautions (I: 56.7%, C: 54.1%, ES: 2.6%-pts); time spent with client (I: 14.5%, C: 8.6%, ES: 5.9 %-pts)	5.0 %-pts	From de Walque 2022 (page 110): study "periods covered overlapped with a significant increase in armed conflict across the country, which may have contributed to the lack of impact of the PBF pilot on service utilization."
Argentina	Gertler et al. 2014; Celhay et al. 2019	Non-randomized difference-in-differences analysis of PBF vs. true controls (i.e., clinics that had not yet started PBF).<br>Data sources were birth certificates and administrative data.<br>ROB: High	**Table 6 had 1 HCP antenatal care (ANC) practice outcome.**<br>Proportion of pregnant women needing tetanus vaccination who received it (ES: 5.6 %-pts). Note that this result comes from section B of the table ("Treatment-on-treated" analysis), which examines the effect of PBF on patients that were enrolled in the "Plan Nacer" insurance program that used PBF.	5.6 %-pts	
Burkina Faso	De Allegri et al. 2018	Non-randomized controlled before-and-after study of PBF vs. true controls. Several PBF types were tested, but the results here are for the "standard PBF" ("T1") group.<br>ROB: Medium	**Box 9.** % of ANC cases receiving 3 key ANC services (weight measurement, blood pressure (BP) measurement, and prescription of iron supplements) (T1 vs. control ES: 8.1 %-pts).<br>**Box 10.** % of ANC cases receiving education on 3 key elements: 1) at least 2 of 5 pregnancy danger signs (vaginal bleeding, fever, fatigue or excessive shortness of breath, hand and face swelling, intense headache or impaired eyesight), 2) nutrition in pregnancy and breastfeeding, and 3) importance of skilled birth attendance and/or having a birth plan (T1 vs. control ES: 4.5 %-pts).<br>**Box 11.** % of children in curative consultations assessed for all 4 IMCI danger signs (able to drink or breastfeed, vomits everything, lethargy or unconsciousness, and convulsion) (T1 vs. control ES: -7.3 %-pts).<br>**Box 12.** % of children in curative consultations assessed for all common childhood illness symptoms according to IMCI (ask for: fever, cough, diarrhea, and ear problem; and checks: weight, temperature, anemia, and vaccination status) (T1 vs. control ES: –22.8 %-pts).	–1.4 %-pts	During the study period, "a nation-wide user fee exemption policy ("*gratuité*") was introduced in June 2016, covering many of services incentivized by PBF" (page 3).
Burundi	Gage et al. 2021	PBF vs. true controls<br>ROB: High	**Personal communication from Anna Gage had 5 HCP ANC practice outcomes (measured by household survey).** Tetanus vaccination (ES: -2.0 %-pts; i.e., [I: 46.4% - 42.8%] - [C: 42.0% - 36.4%]); iron supplementation (ES: -12.0 %-pts; i.e., [I: 67.9% - 72.0%] - [C: 80.2% - 72.3%]); blood test (ES: -0.6 %-pts; i.e., [I: 17.6% - 12.0%] - [C: 23.1% - 16.9%]); breastfeeding within 1 hour (ES: 8.6 %-pts; i.e., [I: 11.6% - 0.5%] - [C: 24.5% - 22.0%]); postnatal check before discharge (ES: -14.4 %-pts; i.e., [I: 42.3% - 58.6%] - [C: 37.2% - 39.1%])	–2.0 %-pts	Values in the square brackets are formatted as: "[follow-up – baseline]". For example, for the tetanus vaccination indicator, 46.4% and 42.8% are the F/U and BL values (respectively) for the intervention group, and 42.0% and 36.4% are the F/U and BL values (respectively) for the controls.
Cameroon	de Walque et al. 2021	RCT of PBF vs. DFF vs. enhanced supervision vs. true control. Intervention was PBF + enhanced supervision + monitoring + limited managerial autonomy of HFs + removal of user fees.<br>ROB: Low	**Table 6 had 2 HCP practice outcomes (see "PBF/post-interact" row).**<br>Overall quality score of child health consultations (ES: –2.1 %-pts); overall quality score of ANC consultations (ES: –5.6 %-pts).	–3.9 %-pts	

Country	References	Methods and risk of bias (ROB)	Results of PBF vs. true controls<br>(C = control arm, ES = effect size, I = intervention arm)	Median effect size (PBF vs. controls)	Comments
The Gambia	Ferguson et al. 2020	RCT of facility intervention vs. community intervention vs. both facility and community intervention vs. controls.<br>ROB: Medium	**Table 5 had 2 HCP practice outcomes (see results for "Facility-based intervention only", which is for the facility only group vs. controls).**<br>• "Referred to delivery" — i.e., pregnant woman was referred by a community birth companion to a health facility for delivery (ES: 4.01 %-pts).<br>• "Accompanied to delivery" — i.e., pregnant woman was accompanied by a community birth companion to a health facility for delivery (ES: 1.50 %-pts).	2.8 %-pts	
Lesotho	Gage et al. 2021	PBF vs. true controls<br>ROB: Medium	**Personal communication from Anna Gage had 3 HCP ANC practice outcomes (measured by household survey).** Tetanus vaccination (ES: -3.5 %-pts; i.e., [I: 69.6% - 64.3%] - [C: 72.3% - 63.5%]); Blood test (ES: -2.7 %-pts; i.e., [I: 96.6% - 97.1%] - [C: 98.2% - 96.0%]); Breastfeeding within 1 hour (ES: -4.5 %-pts; i.e., [I: 100.0% - 64.0%] - [C: 100% - 59.5%])	–3.5 %-pts	
Malawi	Kambala et al. 2017	Non-randomized controlled before-and-after study of PBF vs. true controls.<br>ROB: High	**Table 4 had 13 HCP practice outcomes on labor & delivery.** HCP introduction (ES: -4 %-pts); woman was examined (ES: 6 %-pts); explanation of examination was given (ES: -2 %-pts); medication/treatment given (ES: 29 %-pts); explanation for medication was given (ES: 12 %-pts); blood test done (ES: -2 %-pts); explanation for test was given (ES: 10 %-pts); consent sought before procedures (ES: 1 %-pts); woman encouraged to ask questions (ES: 2 %-pts); offer to have a guardian during delivery (ES: 3 %-pts); privacy was protected (ES: 8 %-pts); BP checked before delivery (ES: 19 %-pts); BP checked after delivery (ES: 19 %-pts).<br><br>**Table 5 had 13 HCP practice outcomes on ANC.** HCP introduction (ES: -9 %-pts); woman was examined (ES: 5 %-pts); explanation of examination was given (ES: 1 %-pts); medication/treatment given (ES: -4 %-pts); explanation for the medication was given (ES: 2 %-pts); blood test done (ES: -2 %-pts); explanation for test was given (ES: -1 %-pts); clinical procedures performed (ES: -3 %-pts); consent sought before procedures (ES: 3 %-pts); woman encouraged to ask questions (ES: 6 %-pts); offer to have guardian (ES: -7 %-pts); privacy was protected (ES: 3 %-pts); BP checked (ES: -28 %-pts).<br><br>**Table 6 had 14 HCP practice outcomes on PNC.** HCP introduction (ES: 2 %-pts); woman was examined (ES: 35 %-pts); explanation of examination was given (ES: -20 %-pts); medication/treatment given (ES: -15 %-pts); explanation for medication was given (ES: -29 %-pts); blood test done (ES: 4 %-pts); explanation for test was given (ES: 3 %-pts); clinical procedures performed (ES: 25 %-pts); consent sought before procedures (ES: 2 %-pts); woman encouraged to ask questions (ES: 23 %-pts); offer to have guardian (ES: -2 %-pts); privacy was protected (ES: 7 %-pts); BP checked (ES: 10 %-pts); baby weighed (ES: 23 %-pts).	2.5 %-pts	
Nigeria	Khanna et al. 2021	RCT of PBF vs. DFF vs. true controls.<br>Intervention was PBF + autonomy + enhanced supervision + enhanced community engagement + training + HCPs encouraged to lower or waive user fees.<br>ROB: Medium	**Table 5 had 3 HCP practice outcomes.**<br>Under-5 exam score (ES: 5.3 %-pts); ANC exam score (ES: 11.3 %-pts); % of HFs w/MCH outreach (ES: 25.1 %-pts). *Note that nearly identical results to those above are in Table 11 in Additional file 3.*<br><br>**Table 8 in Additional file 3 had 3 HCP practice outcomes.**<br>% of HFs with up-to-date vax register (ES: 8.3 %-pts); % of HFs with up-to-date ANC & delivery register (ES: 11.8 %-pts); % of HFs with completed HMIS monthly report (ES: 12.0 %-pts).	11.6 %-pts	

Country	References	Methods and risk of bias (ROB)	Results of PBF vs. true controls (C = control arm, ES = effect size, I = intervention arm)	Median effect size (PBF vs. controls)	Comments
Rwanda (PBF that began in 2002)	Johannes et al. 2008; Meessen et al. 2005; Meessen et al. 2006; Meessen et al. 2007; Rusa et al., 2009; Rowe 2019 (Health Care Provider Performance Review [HCPPR] database, IDNUM 102000001); Soeters et al. 2005; Soeters et al. 2006	Non-randomized controlled before-and-after study of PBF vs. true controls.<br>Continuous quality improvement + decentralized management of HF + contracting-in + community outreach + performance-based financial incentive to HF + mosquito bednets for community **(from q18_label) HCPPR ComplDnew 10200000112**<br>ROB: Very high	**HCPPR database OUT_ID 10200000103.**<br>Outcome = Number of deliveries referred by the health center per health center	375.2 %-pts	Effect size retrieved from HCPPR database "All effect sizes (from ph1_2_es2May2020).xlsx" (Obs = 3373).
		Non-randomized controlled before-and-after study of PBF vs. true controls.<br>Continuous quality improvement + decentralized management of HF + contracting + consumer ratings of HCPs + performance-based financial incentive for HF + non-performance-based financial incentive for HF + mosquito bednets for community **(from q19_label) HCPPR ComplDnew 10200000113**<br>ROB: Very high	**HCPPR database OUT_ID 10200000103.**<br>Outcome = Number of deliveries referred by the health center per health center	3.5 %-pts	Effect size retrieved from HCPPR database "All effect sizes (from ph1_2_es2May2020).xlsx" (Obs = 3374).
Senegal	Gage et al. 2021	PBF vs. true controls<br>ROB: Medium	**Personal communication from Anna Gage had 5 HCP ANC practice outcomes (measured by household survey).** Tetanus vaccination (ES: 0.6 %-pts; i.e., [I: 73.3% - 69.4%] - [C: 74.1% - 70.8%]); Iron supplementation (ES: 2.1 %-pts; i.e., [I: 90.0% - 88.2%] - [C: 90.2% - 90.5%]); Blood test (ES: 3.8 %-pts; i.e., [I: 82.9% - 70.1%] - [C: 86.5% - 77.5%]); Breastfeeding within 1 hour (ES: -0.7 %-pts; i.e., [I: 54.0% - 60.6%] - [C: 55.2% - 61.1%]); Postnatal check before discharge (ES: -1.0 %-pts; i.e., [I: 88.3% - 86.1%] - [C: 92.1% - 88.9%]).	0.6 %-pts	
Tajikistan	Ahmed et al. 2023	Non-randomized controlled before-and-after study of PBF vs. true controls. PBF includes autonomy and enhanced supervision (page 3).<br>ROB: Medium	**Table 5 had 9 HCP practice outcomes.**<br>**There were 5 outcomes for adult consultations.** Proportion of core clinical history items asked (ES: 10.2 %-pts); CVD risk score calculated (ES: -5.8 %-pts); CVD risk score properly calculated (ES: 2.1 %-pts); clinician washed their hands before starting the exam (ES: -14.8 %-pts); BP measured (ES: 7.5 %-pts).<br><br>**There were 4 HCP practice outcomes for child consultations.** Vaccination history checked (ES: 9.7 %-pts); Clinician washed their hands before starting the exam (ES: -9.1 %-pts); Height and weight measured (ES: 12.0 %-pts); Proportion of core physical exam activities completed (ES: 10.7 %-pts).	7.5 %-pts	Results for HCP practice outcomes in Appendix Table A6 were not used for 3 reasons. First, there was overlap with Table 5 outcomes. Second, it seemed that outcomes in an appendix would not be primary outcomes. Third, for the one outcome that appears in both Tables 5 and A6 (i.e., BP measured), the results were slightly different. This last point raised the possibility that analytic methods were different or perhaps there were errors.

Country	References	Methods and risk of bias (ROB)	Results of PBF vs. true controls<br>(C = control arm, ES = effect size, I = intervention arm)	Median effect size (PBF vs. controls)	Comments
Tanzania	Binyaruka et al. 2015	Non-randomized controlled before-and-after study of PBF vs. true controls.<br>ROB: Medium	**Table 2 had 2 HCP ANC practice outcomes (measured by household survey, but the analysis was restricted to women who had received ANC).** At least 2 doses of IPT during ANC (ES: 10.3 %-pts); HIV treatment during ANC (ES: -0.3 %-pts)<br><br>**Table 4 had 1 HCP ANC practice outcome (measured by patient exit interview during a health facility survey).** ANC content of care index (ES: -6.0 %-pts). See comment.	–0.3 %-pts	• Not found in HCPPR.<br>• The ANC content of care index had values from 0 to 1, and the ES was –0.06. Thus, on a 0 to 100 scale, the ES would be –6.0 points. As the index reflects the % of tasks done, the ES can be interpreted to be a %-pt change.<br>• Table 2 had some outcomes related to HCP performance, but they were excluded because they were measured by household surveys, and thus results could be influenced both by patient care-seeking behaviors and HCP practices (e.g., polio vaccine at birth measured for all births, regardless of whether the delivery was at a health facility).
Zambia	Friedman et al. 2016; Zeng, Shepard, Nguyen, et al. 2018	RCT of PBF vs. DFF vs. true controls ("control 2")<br>PBF was a "contracting in" model (page 10 of WB 2016) and included provision of commodities and equipment (page 11 of WB 2016).<br>ROB: Low	**Table 16 had 11 HCP ANC practice outcomes (ANC index outcome excluded because it was based on the other 11 outcomes).** Weighed (ES: 6.0 %-pts); BP measured (ES: 8.0 %-pts); urine sample collected (ES: -9.0 %-pts); blood sample collected (ES: -2.0 %-pts); abdomen measured (ES: 9.0 %-pts); abdomen palpated (ES: 12.0 %-pts); advice on diet (ES: 2.0 %-pts); iron folate given (ES: 2.0 %-pts); iron folate side effects explained (ES: -18.0 %-pts); given antimalarials (ES: 0 %-pts); pregnancy danger signs explained (ES: 0 %-pts).<br><br>**Table 18 had 5 HCP child health practice outcomes (quality of care index outcome excluded because it was based on the other 5 outcomes).** Asked age (ES: 2.0 %-pts); weighed child (ES: 6.0 %-pts); measured height (ES: -2.0 %-pts); plotted growth chart (ES: -2.0 %-pts); physical exam done (ES: -8.0 %-pts).<br>• Note that one HCP postnatal care practice outcome from Table 17 (vitamin A given after delivery; ES: 2.5 %-pts) was excluded because it was measured in a household survey, and there were already 16 indicators from health facility surveys. Inclusion of this one effect size would not have affected the overall median.	1.0 %-pts	Results from Gage and Bauhoff 2021 were not used because several outcomes overlapped with those in the Friedman et al. 2016 report, and the Friedman et al. 2016 study used more robust measurement methods (health facility survey).

Country	References	Methods and risk of bias (ROB)	Results of PBF vs. true controls<br>(C = control arm, ES = effect size, I = intervention arm)	Median effect size (PBF vs. controls)	Comments
Zimbabwe	World Bank 2016	Non-randomized controlled before-and-after study of PBF vs. “business-as-usual” controls (see bottom of page 26 of WB 2016c).<br>Intervention was PBF (called RBF in the WB 2016 report) + limited autonomy + process for obtaining community feedback + removal of user fees.<br>Both study arms received drugs from donors.<br>ROB: Medium	**Table 25 had 6 HCP ANC practice outcomes. From patient exit interviews at HFs.** Weighed (ES: -0.5 %-pts); BP measured (ES: 4.1 %-pts); urine sample collected (ES: 1.1 %-pts); blood sample collected (ES: -5.6 %-pts); abdomen measured (ES: 3.6 %-pts); abdomen palpated (ES: -2.1 %-pts).<br><br>**Table 26 had 9 HCP ANC practice outcomes. From observations of consultations.** Measure BP (ES: -1.5 %-pts); Weigh the client (ES: -5.8 %-pts); check for signs of anemia (ES: 15.4 %-pts); examine hands for edema (ES: 16.7 %-pts); palpate the client's abdomen for uterine height (ES: -1.5 %-pts); perform or refer for urine test for proteinuria (ES: 6.4 %-pts); prescribed iron or folic acid (IFA) or both (ES: -15.2 %-pts); gave supply of iron or folic acid (IFA) or both (ES: -16.2 %-pts); Explained side effects of iron or folic acid (ES: -0.7 %-pts).<br><br>**Table 29 had 5 HCP practice outcomes on child health. From patient exit interviews at HFs.** Ask age of the child (ES: -4.2 %-pts); weigh child (ES: 2.1 %-pts); measure height of the child (ES: 7.6 %-pts); plot weight or height against a growth chart (ES: 12.8 %-pts); did HCP physically examine the child (ES: 1.4 %-pts).<br><br>**Table 30 had 15 HCP practice outcomes on child health. From observations of consultations.** Correctly classifies general danger signs (ES: 21.2 %-pts); correctly classifies cough/difficult breathing (ES: 30.7 %-pts); correctly manages cough/difficult breathing (ES: 38.2 %-pts); correctly classifies diarrhea (ES: -9.4 %-pts); correctly manages diarrhea (ES: -6.2 %-pts); correctly classifies fever for malaria (ES: -13.6 %-pts); correctly manages fever (ES: -6.2 %-pts); correctly classifies ear problem (ES: -0.5 %-pts); correctly manages ear problem (ES: -8.3 %-pts); correctly classifies nutritional status (ES: 3.6 %-pts); correctly manages nutritional problem (ES: 14.1 %-pts); correctly classifies anemia (ES: -3.4 %-pts); correctly manages anemia (ES: -7.5 %-pts); correctly classifies and manages HIV (ES: 5.2 %-pts); correctly vaccinates (ES: 48.7 %-pts).	–0.5 %-pts	• Results from Gage and Bauhoff 2021 were not used because several outcomes overlapped with those in the World Bank 2016 report, and the World Bank 2016 study used more robust measurement methods (health facility survey).<br>• Note that the –0.5 %-pt result was the median of all 35 effect sizes measured by either exit interviews or consultation observations. As a sensitivity analysis that was the median of 24 effect sizes, only from consultation observations, the result was similar: –1.1 %-pts.

REFERENCES

Ahmed T, Arur A, de Walque D, Shapira G. Incentivizing quantity and quality of care: evidence from an impact evaluation of performance-based financing in the health sector in Tajikistan. *Economic Development and Cultural Change.* 2023;71(2):667–707.

Basinga P, Gertler PJ, Binagwaho A, Soucat ALB, Sturdy J, Vermeersch CMJ. Effect on maternal and child health services in Rwanda of payment to primary health-care providers for performance: an impact evaluation. *Lancet.* 2011;377(9775):1421–28.

Binyaruka P, Patouillard E, Powell-Jackson T, Greco G, Maestad O, Borghi J. Effect of paying for performance on utilisation, quality, and user costs of health services in Tanzania: a controlled before and after study. *PLoS One*. 2015;10(8):e0135013. Also, personal communication from Timothy Powell-Jackson and Josephine Borghi, London School of Hygiene and Tropical Medicine, on December 2, 2024.

Celhay P, Gertler P, Giovagnoli P, Vermeersch C. Long-run effects of temporary incentives on medical care productivity. *American Economic Journal: Applied Economics* 2019;11(3):92–127.

De Allegri M, Lohmann J, Schleicher M, Brenner S, Koulidiati J-L. Results-Based Financing for Health Impact Evaluation in Burkina Faso: Results Report. Institute of Public Health, Heidelberg University, Germany. Version 2 (June 22nd, 2018). Note that this report was obtained via email from Damien De Walque, The World Bank, on November 27, 2024. The report was not found on the internet.

de Walque D, Chukwuma A, Ayivi-Guedehoussou N, Koshkakaryan M. Invitations, incentives, and conditions. A randomized evaluation of demand-side interventions for health screenings. *Soc Sci Med* 2022;296:114763.

de Walque D, Kandpal E, Wagstaff A, Friedman J, Neelsen S, Piatti-Fünfkirchen M, et al. Improving effective coverage in health: do financial incentives work? Washington, DC: World Bank; 2022. Accessed November 24, 2024. https://www.worldbank.org/en/research/publication/improving-effective-coverage-in-health

de Walque D, Robyn PJ, Saidou H, Sorgho G, Steenland M. Looking into the performance-based financing black box: evidence from an impact evaluation in the health sector in Cameroon. *Health Policy Plan.* 2021;36(6):835–47.

Engineer CY, Dale E, Agarwal A, Agarwal A, Alonge O, Edward A, Gupta S, et al. Effectiveness of a Pay-for-Performance Intervention to Improve Maternal and Child Health Services in Afghanistan: A Cluster-Randomized Trial. *Int J Epidemiol.* 2016;45(2): 451–59.

Ferguson L, Hasan R, Boudreaux C, et al. Results-based financing to increase uptake of skilled delivery services in The Gambia: using the ‘three delays’ model to interpret midline evaluation findings. *BMC Pregnancy Childbirth.* 2020;20(1):712.

Friedman J, Kandpal E, Nguyen H, et al. Impacts of the Kyrgyz Republic performance-based health financing pilot program on maternal and neonatal health. Unpublished report dated February 2020. Source of this report was Damien de Walque, The World Bank, on December 3, 2024.

Friedman J, Qamruddin J, Chansa C, Das AK. Impact evaluation of Zambia's health results-based financing pilot project. Washington, DC: World Bank; 2016.

Gage A, Bauhoff S. The effects of performance-based financing on neonatal health outcomes in Burundi, Lesotho, Senegal, Zambia and Zimbabwe. *Health Policy Plan.* 2021;36(3):332–40. Also, personal communication from Anna Gage, Institute for Health Metrics and Evaluation, University of Washington, on December 4, 2024.

Gertler P, Giovagnoli P, Martinez S. Rewarding provider performance to enable a healthy start to life: evidence from Argentina's Plan Nacer. Policy Research Working Paper 6884. Washington, DC: World Bank; 2014. Accessed December 1, 2024. https://documents1.worldbank.org/curated/en/376131467998218480/pdf/903550BRI0Rewa00Box385311B00PUBLIC0.pdf

Huillery E, Seban J. Financial incentives, efforts, and performances in the health sector: experimental evidence from the Democratic Republic of Congo. *Economic Development and Cultural Change.* 2021;69(3):1115–1164.

Johannes L, Mullen P, Okwero P, Schneidman M. Performance-based contracting in health. The experience of three projects in Africa. OBApproaches. 2008;19. Accessed December 16, 2008. http://www.gpoba.org/docs/OBApproaches19_Africa_Health.pdf.

Kambala C, Lohmann J, Mazalale J, Brenner S, Sarker M, Muula AS, De Allegri M. Perceptions of quality across the maternal care continuum in the context of a health financing intervention: Evidence from a mixed methods study in rural Malawi. *BMC Health Serv Res.* 2017;17(1):392. Also, personal communications from Manuela De Allegri, Heidelberg University, on December 8, 2024; and Christabel Kambala, Malawi University of Business and Applied Sciences, on December 10, 2024.

Khanna M, Loevinsohn B, Pradhan E, et al. Improving maternal and neonatal health in Nigeria: performance-based financing versus decentralized facility financing. *BMC Med.* 2021;19:224.

Kruk ME, Gage AD, Arsenault C, et al. High-quality health systems in the Sustainable Development Goals era: time for a revolution. *Lancet Glob Health.* 2018;6(11):e1196–e1252.

Meessen B, Kashala JP, Musango L. Output-based payment to boost staff productivity in public health centres: contracting in Kabutare district, Rwanda. *Bull World Health Organ.* 2007;85(2):108–115.

Meessen B, Musango L, Kashala JP, Lemlin J. Reviewing institutions of rural health centres: the Performance Initiative in Butare, Rwanda. *Trop Med Int Health.* 2006;11(8):1303–1317.

Meessen B, Musango L, Soeters R. Comparison of two output based schemes in Butare and Cyangugu provinces. Presentation given at GPOBA Trust Fund, in support of PRSC II, Kigali, October 12, 2005. Slides 1-23. Accessed April 2, 2010. http://www.devpartners.gov.rw/docs/Coordination%20Structures/Clusters/Health%20Sector/index.php?dir=Minutes%2F2005.10.11%2Fannexes%2F&download=PresentationGPOBAstudy+V210905.ppt

Rowe AK, Rowe SY, Peters DH, Holloway KA, Chalker J, Ross-Degnan D. The effectiveness of strategies to improve health worker knowledge in low- and middle-income countries and the association between knowledge and clinical practice: a systematic review. Poster presentation by AK Rowe at the 66th Annual Meeting of the ASTMH, November 5–9, 2017, Baltimore, Maryland. Abstract 233.

Rowe SY, Peters DH, Holloway KA, Chalker J, Ross-Degnan D, Rowe AK. A systematic review of the effectiveness of strategies to improve health care provider performance in low- and middle-income countries: Methods and descriptive results. *PLoS One.* 2019;14(5):e0217617.

Rusa L, Schneidman M, Fritsche G, Musango L. Performance incentives for global health: potential and pitfalls. Eichler R, Levine R, and the Performance-Based Incentives Working Group Rwanda: Versa Press: East Peoria, Illinois; 2009. Chapter 10, Rwanda: Performance-based financing in the public sector; p 189-214. Accessed July 7, 2009. http://www.cgdev.org/doc/books/PBI/06_CGD_Eichler_Levine-Ch10.pdf.

Shapira G, Clarke-Deelder E, Booto BM. Impacts of performance-based financing on health system performance: evidence from the Democratic Republic of Congo. *BMC Med.* 2023;21:381.

Shapira G, Kalisa I, Condo J, Humuza J, Mugeni C, Nkunda D, Walldorf J. Going beyond incentivizing formal health providers: evidence from the Rwanda Community Performance-Based Financing Program. *Health Econ.* 2018;27(12):2087–2106.

Sherry TB, Bauhoff S, Mohanan M. Multitasking and heterogeneous treatment effects in pay-for-performance in health care: evidence from Rwanda. *American Journal of Health Economics.* 2017;3(2):192–226.

Soeters R, Habineza C, Peerenboom PB. Performance-based financing and changing the district health system: experience from Rwanda. *Bull World Health Organ.* 2006;84(11):884–889.

Soeters R, Musango L, Meessen B. Comparison of two output based schemes in Butare and Cyangugu provinces with two control provinces in Rwanda. Final Version. Washington D.C.: World Bank and Ministry of Health, Rwanda; 2005. 45p. Global Partnership on Output-Based Aid (GPOBA).

Van de Poel E, Flores G, Ir P, O'Donnell O, Van Doorslaer E. Can vouchers deliver? An evaluation of subsidies for maternal health care in Cambodia. *Bull World Health Organ.* 2014;92(5):331–39.

World Bank. Rewarding provider performance to improve quality and coverage of maternal and child health outcomes: Zimbabwe results-based financing pilot programme. Evidence to inform policy and management decisions. World Bank, Washington, DC, 2016. Accessed November 24, 2024. https://www.researchgate.net/publication/326718836_Rewarding_Provider_Performance_to_Improve_Quality_and_Coverage_of_Maternal_and_Child_Health_Outcomes_Evidence_and_lessons_from_Zimbabwe_results_based_financing_project

Zeng W, Cros M, Wright KD, Shepard DS. Impact of performance-based financing on primary health care services in Haiti. *Health Policy Plan.* 2013;28(6):596–605.

Zeng W, Shepard DS, Nguyen H, Chansa C, Das AK, Qamruddin J, Friedman J. Cost-effectiveness of results-based financing, Zambia: a cluster randomized trial. *Bull World Health Organ*. 2018;96(11):760–771.

Text S7. Low-dose, high-frequency training

A rapid review of low-dose, high-frequency training to improve healthcare provider practices in low- and middle-income countries

OBJECTIVE

Characterize the effect of low-dose, high-frequency training (LDHFT) on healthcare provider (HCP) practices in low- and middle-income countries (LMICs).

METHODS

- Definition of LDHFT. An educational activity with at least two short training sessions (i.e., lasting a few minutes up to 4 days) that are spaced at least one week apart, that occur at HCPs' worksite, and that include practicing clinical skills (simulations or treating real patients). This definition is based on descriptions from Sutton et al. (2011) (i.e., brief [e.g., lasting 2 minutes], intermittent [e.g., spaced 1–3 months] bedside "booster" training that includes practicing clinical skills) and Jhpiego (2016) (i.e., "short, targeted in-service simulation-based learning activities, which are spaced over time and reinforced with structured, ongoing practice sessions on the jobsite"). Note that LDHFT programs are sometimes accompanied by other intervention components, such as quality improvement collaboratives, maternal death reviews, supportive supervision visits, mobile mentoring (by phone or SMS), or peer-assistant learning [van Tetering 2023]. In such cases, the other (non-LDHFT) intervention components are recorded so that the analysis can differentiate between evaluations of LDHFT as a stand-alone intervention versus LDHFT plus other components. The provision of equipment needed for the skills taught via LDHFT and for simulations to practice the skills (e.g., mannequins and newborn resuscitation instruments) and the provision basic training materials (e.g., books and job aids) were considered to be part of LDHFT (i.e., not separate interventions), but the provision of commodities (e.g., medicines) was considered to be a separate intervention component (and thus the full intervention description would be, for this example: LDHFT plus provision of medicines). The assignment and training of an already-existing HCP in the facility to be a peer practice coordinator or local champion who facilitates and encourages HCPs to practice skills taught via LDHFT was considered to be part of LDHFT, but having staff outside the facility support LDHFT (e.g., supervisors or mentors) was considered to be a separate intervention component (and thus the full intervention description would be, for this example: LDHFT plus supervision [with "supervision" being a general term describing any external staff person or activity that supports HCPs to develop and maintain skills]).

- Definition of HCP. Health workers that provide health care to patients or that perform other health-related activities (e.g., educating patients or community members) in a hospital, outpatient clinic, or community setting.

- Inclusion criteria. Eligible studies: 1) were done in an LMIC, 2) examined the quantitative effect of LDHFT on an eligible outcome, and 3) used an eligible study design. Eligible outcomes were: 1) HCP practices (or processes of care), such as correct patient assessment,

diagnosis, treatment, counseling, referral, preventive services, and documentation (which reflect behaviors when managing real patients or simulated clients that HCPs believe are real patients); and 2) health outcomes, such as a maternal mortality rate. Eligible study designs were: 1) controlled before-and-after study (with or without randomization), 2) post-intervention-only study with randomized controls, or 3) interrupted time series. Eligible effect sizes were based on a comparison of a LDHFT intervention arm and a no-intervention control arm, or a head-to-head study design that compared two interventions and the difference between the two interventions was LDHFT (i.e., to evaluate the marginal effect of LDHFT given the presence of other intervention components) (e.g., LDHFT + a 10-day classroom-based training versus a 10-day classroom-based training only).

- Exclusion criteria. 1) Outcomes of "inputs" or "structural quality" (e.g., availability of medicines), 2) HCP knowledge outcomes, 3) HCP skill outcomes measured during simulations (i.e., not HCP practice outcomes, which are behaviors when managing patients), 4) outcomes of community-level coverage that included all community members regardless of whether they had sought care from HCPs (e.g., proportion of all newborns that received polio vaccination, as measured by a household survey), 5) effect sizes based on a comparison of two active interventions and the difference between the two interventions is not LDHFT (e.g., a LDHFT intervention arm compared to a supervision intervention arm), and 6) outcomes that are related to already-used outcomes and that have a denominator that includes subjects unlikely to need the action reflected in the numerator (e.g., in Dumont et al. [2013], which has the outcomes "hysterectomy rate" and "hysterectomy rate among women with hemorrhage", the latter outcome would be used because hysterectomy would be generally unnecessary in women without hemorrhage). Outcomes of inputs and HCP knowledge were excluded because they are only weakly correlated with HCP practices [Kruk et al. 2018; Rowe et al. 2017]. Outcomes of HCP skills measured during simulations were excluded because it is unclear how well these outcomes reflect behaviors with real patients. Outcomes of community coverage that included all community members regardless of whether they had sought care from HCPs were excluded because achieving these outcomes requires a combination of care-seeking by community members and correct HCP practices. Although patient health outcomes were initially excluded because the causal pathway between HCP practices and health outcomes can be long and complex, with many other factors potentially contributing to the health outcomes, this decision was changed (and thus health outcomes are eligible) because nearly all studies examined emergency health topics, for which HCP practices usually have a direct and almost immediate effect on health outcomes (e.g., preventing and treating postpartum hemorrhage).

- Literature search. First, the bibliography of a recent overview on LDHFT was hand-searched [Mukeshimana 2022]. Second, three experts on LMIC training interventions were contacted for studies (Julia Bluestone and Cherrie Evans [Jhpiego, USA] and Daniele Trevisanuto [University of Padua, Italy] on December 5 and 6, 2024). Third, the database of a systematic review that included LDHFT studies was searched [Rowe 2021]. Fourth, a search on PubMed was performed on December 5, 2024, using the search terms "low-dose, high-frequency training" (no time restriction). This search yielded 85 citations. Fifth, the above search steps identified two literature reviews that likely included LDHFT studies [van Tetering 2023; Fransen 2020], and the bibliographies of these reviews were hand-searched.

- Data abstraction. Effect sizes (or outcome values needed to calculate effect sizes) from eligible outcomes were abstracted from the original LDHFT study reports, which are listed in the tables below. Effect sizes were abstracted by one person (AKR). For some studies (e.g., Odusanya et al. 2004, and Kumar et al. 2015), the data had already been abstracted and effect sizes calculated by a large systematic review (the Health Care Provider Performance Review, or HCPPR] [Rowe et al. 2019]); and thus, the effect size from this systematic review was used.

- Risk of bias (ROB) assessment. The ROB was based on the study design and study limitations: low risk for randomized-controlled trials (RCTs) with zero or one minor limitation; moderate risk for interrupted time series (ITS) studies with zero or one minor limitation, and RCTs with one main limitation or two minor limitations; high risk for non-randomized controlled before-and-after studies, ITS studies with one main limitation or two minor limitations, and RCTs with two main limitations; and very high risk for studies that have more limitations than those listed for the high ROB level. Note that RCTs with only one facility or cluster per study arm were categorized as high ROB.

- Analysis. Effect sizes were measured in terms of absolute percentage-point (%-pt) improvements. For outcomes measured as a percentage that could plausibly change over the entire range from 0 to 100% (i.e., most HCP practice outcomes, such as percentage of children weighed), the effect size = $(\text{follow-up} - \text{baseline})_{\text{intervention}} - (\text{follow-up} - \text{baseline})_{\text{controls}}$. For continuous unbounded outcomes and for percentage outcomes that are only expected to change over a narrow range (e.g., neonatal mortality), the effect size = $100\% \times \{[(\text{follow-up} - \text{baseline})/\text{baseline}]_{\text{intervention}} - [(\text{follow-up} - \text{baseline})/\text{baseline}]_{\text{controls}}\}$. Often, LDHFT studies did not present effect sizes calculated this way, and the reports did not include baseline and follow-up outcome values that could be used to calculate effect sizes. Instead, studies reported similar kinds of effect sizes (e.g., difference-in-differences effects, or odds ratios for health outcomes) that were estimated with statistical regression models that adjusted for potential confounders and clustering. When available, these model-based effect size estimates were used. For each study comparison, the median of effect sizes (MES) from HCP practice outcomes was calculated (if such outcomes were available); and (separately), the MES from patient health outcomes was calculated (if such outcomes were available). The distribution of the study-comparison-specific MES values was described with a median and interquartile range (separately for HCP practice outcomes and patient health outcomes). The main analysis included studies with all ROB levels, and a sensitivity analysis only included studies with low or moderate ROB. Additionally, one study by Kc et al. (2019) was excluded because the intervention did not fit the definition of LDHFT used in this rapid review. However, as the deviation from the definition was minor, the results were used in a sensitivity analysis. One additional sensitivity analysis compared median MES values for stand-alone LDHFT versus LDHFT plus other intervention components.

- Supplemental information from study investigators. When there were questions about measurement methodology, the interpretation of results for a particular study, or data from unpublished studies, an attempt was made to contact the study investigators for information. These contacts are listed as personal communications in the tables and reference list below.

RESULTS

- In the main analysis, 14 study comparisons (and thus 14 MES values) were included from 11 LMICs (**Table S4** in the **Online Supplementary Document**, below). All articles were in English, except one in Spanish and one in Chinese. Seven study comparisons tested stand-alone LDHFT; four studies tested LDHFT plus supervision; one study tested LDHFT plus supervision plus provision of medicines; one study tested LDHFT plus quality improvement (QI) (with QI collaboratives); and one study tested LDHFT plus supervision plus QI (with mortality audit committees). Six study comparisons had a low ROB, three had a moderate ROB, four had a high ROB, and one had a very high ROB. Sixty-nine studies from 35 countries were excluded (details available on request), mostly because the intervention did not fit the LDHFT definition used in this rapid review or because there were no eligible outcomes. One study excluded from the main analysis but included in a sensitivity analysis (Kc et al. 2019) had a low ROB, and the intervention was stand-alone LDHFT.

- *Main and sensitivity analyses that exclude Kc et al. (2019)*
  - For the main analysis, among the 13 included study comparisons with an **HCP practice outcome** (including all ROB levels), the median MES was 17.5 %-pts (interquartile range: 11.9 to 42.7 %-pts). Overall, the quality of evidence was moderate. For the sensitivity analysis (with only low or moderate ROB levels), among the seven study comparisons, the median MES was 16.5 %-pts (interquartile range: 6.1 to 42.4 %-pts). Overall, the quality of evidence was high.
  - For the main analysis, among the nine included study comparisons with a **patient health outcome** (including all ROB levels), the median MES was 34.0 %-pt (interquartile range: 15.0 to 60.0 %-pts). Overall, the quality of evidence was high. For the sensitivity analysis (with only low or moderate ROB levels), among the eight study comparisons, the median MES was 38.1 %-pts (interquartile range: 28.1 to 60.3 %-pts). Overall, the quality of evidence was high.

- *Sensitivity analysis that includes all ROB levels and includes Kc et al. (2019)*
  - Among the 14 included study comparisons with an **HCP practice outcome**, the median MES was 19.9 %-pt (interquartile range: 12.7 to 49.5 %-pts). Overall, the quality of evidence was moderate.
  - Among the 10 included study comparisons with a **patient health outcome**, the median MES was 33.2 %-pt (interquartile range: 16.5 to 55.6 %-pts). Overall, the quality of evidence was high.

- *Sensitivity analysis that compared stand-alone LDHFT versus LDHFT plus other intervention components*
  - For **HCP practice outcomes**, when Kc et al. (2019) was excluded, the median MES of LDHFT plus other interventions (24.8 %-pts; N = 6 study comparisons) was 7.3 %-pts greater than the median MES of stand-alone LDHFT (17.5 %-pts; N = 7 study comparisons). When Kc et al. (2019) was included, the median MES of LDHFT plus other interventions (24.8 %-pts; N = 6 study comparisons) was 4.9 %-pts greater than the median MES of stand-alone LDHFT (19.9 %-pts; N = 8 study comparisons).

- For **patient health outcomes**, when Kc et al. (2019) was excluded, it was not possible to make a valid comparison between these two intervention groups because there were only two studies of stand-alone LDHFT with patient health outcomes. When Kc et al. (2019) was included, the median MES of LDHFT plus other interventions (42.2 %-pts; N = 7 study comparisons) was 21.2 %-pts greater than the median MES of stand-alone LDHFT (21.0 %-pts; N = 3 study comparisons), although the validity of this comparison is questionable because there were only three studies of stand-alone LDHFT with patient outcomes.

- Although not part of the main or sensitivity analysis, a head-to-head RCT by Ugwa et al. (2020) compared LDHFT plus mobile mentoring versus traditional off-site 8-day training on maternal and newborn care and found small differences after 12 months. There were only HCP knowledge and skill outcomes (i.e., not HCP practice outcomes). For knowledge, from baseline to 12-month follow-up, the LDHFT group improved by 21.0 %-pts, and the traditional training group improved by 15.4 %-pts (i.e., 5.6 %-pt difference between the two groups). For skills, from baseline to 12-month follow-up, the LDHFT group improved by 51.0 %-pts, and the traditional training group improved by 47.4 %-pts (i.e., 3.6 %-pt difference between the two groups).

COMMENTS

- It was challenging to define LDHFT for this rapid review because the label was used differently in different studies. For example, for Sutton et al. (2011), the “low dose” in LDHFT meant 2-minute practice sessions every 1–3 months; whereas, for Gomez et al. (2018), “low dose” meant 4-day trainings. Some investigators (e.g., Fritz et al. [2017] and Walker et al. [2020]) did not use the LDHFT label even though their interventions were essentially LDHFT. Additionally, the diversity of co-interventions (see above) complicated efforts to characterize the effectiveness of LDHFT.

- Some LDHFT interventions had a high frequency and intensity of external engagement by mentors and facilitators (e.g., 2-day facilitator visits to facilities every 2 weeks in the study by Bassani [unpublished] & Zombre [2021], and weekly–monthly mentoring sessions in the study by Shikuku [2019]). This intensity raises questions about the cost, scalability, and sustainability of LDHFT.

Table S4. The effect of low-dose, high-frequency training (LDHFT) on healthcare provider (HCP) practice outcomes in low- and middle-income countries (LMICs)

Country	Reference	Methods and risk of bias (ROB)	Results of LDHFT vs. controls<br>(BL = baseline, C = control arm, ES = effect size, F/U = follow-up, I = intervention arm)	Median effect size (LDHFT vs. controls)	Comments
China	Zhang et al. 2008	• RCT (intervention arm vs. true control arm)<br>• Health topic: Pain management<br>• Setting: Hospital<br>• Study does not call the intervention LDHFT<br>• Intervention: Training was delivered on-site, weekly, over 5 weeks; and included lecture, discussion, and practice with a simulated patient. Teaching materials (books, pain assessment materials, and a job aid) and pain assessment forms were provided to HCPs.<br>• ROB: High (from HCPPR database)<br>• From HCPPR database, COMP_IDnew = 28420000112	**HCP practice outcomes from HCPPR database (Excel file "**All effect sizes (from ph1_2_es2May2020)**")**<br>• % HCPs who used pain rating scale correctly. ES: 55.1 %-pts	**HCP practice outcome MES:** 55.1 %-pts	
Ghana	Gomez et al. 2018; Willcox et al. 2017	• RCT (intervention arm vs. true control arm)<br>• Health topic: perinatal mortality<br>• Setting: hospitals with at least 30 births/month<br>• Study calls intervention "LDHFT" (title)<br>• Intervention: two 4-day low-dose sessions (1 month apart) for facility HCP birth attendants; 1-day peer practice coordinator training after first low-dose session; high-frequency practice sessions using anatomic models (weekly, for 1 year); mMentoring with SMS reminder messages and quizzes; mentoring calls between master mentors and peer practice coordinators, and between project staff and master mentors; health information officer training; data collection and use training; supply of simulators, newborn resuscitation equipment, and delivery sets provided at first low-dose training session (Table 1). The low-dose sessions included simulation practice.<br>• ROB: Low	**Health outcomes (Table 5, adjusted risk ratios [RR])**<br>• 24-hour newborn mortality rate per 1000 facility live births: RR = 0.30 (ES: 70% reduction)<br>• Intrapartum stillbirth rate (% of facility births that resulted in intrapartum stillbirth): RR = 0.48 (ES: 52% reduction)<br><br>HCP skill outcomes **(not HCP practices)** measured at baseline and 1-year follow-up (ES based on uncontrolled pre-post design) from Table 6:<br>• 1st low-dose training: scores improved by 31 %-pts (BL: 44%; F/U: 76%). ES: 31 %-pts based on model that adjusted for clustering of HCPs in facilities. Given the rough estimate of secular trend of 1 %-pt (see Comment), the adjusted ES is 30 %-pts.<br>• 2nd low-dose training: scores improved by 28 %-pts (BL: 52%; F/U: 81%). ES: 28 %-pts based on model that adjusted for clustering of HCPs in facilities. Given the rough estimate of secular trend of 6 %-pt (see Comment), the adjusted ES is 22 %-pts.	**Health outcome MES:** 61%	Study had no HCP practice outcomes; however, it did have HCP skill (OSCE) outcomes, which were measured at baseline (before each of the two low-dose training sessions) and 1 year later. There were four implementation waves (beginning in Sept 2014, Mar 2015, Sept 2015, and Mar 2016 [see Figure 1]) and thus four baselines measured (in Mar–Aug 2014, Sept 2014–Feb 2015, Mar–Aug 2015, and Sept 2015–Feb 2016). The baseline skill measures for training 1 (for the four implementation waves) were 46%, 46%, 47%, and 47%; and baselines for training 2 were 51%, 49%, 55%, and 57% (results provided by authors). These results suggest that over time (from Mar 2014 to Feb 2016), baseline (i.e., control) skills improved by 1 %-pt for training 1 content and by 6 %-pts for training 2 content.
Kenya	Shikuku et al. 2019	• Controlled pre-post study with non-randomized true control group<br>• Health topic: Intrapartum fetal death<br>• Setting: High-volume hospitals<br>• Study calls intervention low-dose, high-frequency mentorship (not exactly "LDHFT")<br>• Intervention (implemented in 1 hospital):<br>1) LDHF mentorship was a 6-month period with two types of mentorship to HCPs: on-site primary health	**HCP practice outcomes, from baseline to 10 months post-intervention (see methods comment)**<br>**From Table 3:**<br>Breech deliveries (BL I: 1.48%; F/U I: 0.73%; BL C: 1.80%; F/U C: 2.11%; ES: -67.3 %-pts); assisted vaginal deliveries, vacuum extraction (BL I: 0.18%; F/U I: 2.46%; BL C: 0.08%; F/U C: 0.09%; ES: 1223.4 %-pts).<br>**From page 5:**<br>Obstetric cases referred for caesarean delivery (BL I:	**HCP practice outcome MES:** 109.1 %-pts<br><br>**Health outcome MES:** 80.4 %-pts	• **Methods comment.** ES = % change over time in intervention arm minus % change over time in control arm = 100% x {[(intervention follow-up – intervention baseline)/intervention baseline] – [(control follow-up – control baseline)/control baseline]}. For the one outcome for which a decrease is an improvement (i.e.,

Country	Reference	Methods and risk of bias (ROB)	Results of LDHFT vs. controls (BL = baseline, C = control arm, ES = effect size, F/U = follow-up, I = intervention arm)	Median effect size (LDHFT vs. controls)	Comments
		care-based mentorship (low-dose small content packages of simulation-based learning, for individuals and HCP groups, plus the mentor observed HCP-patient interactions and provided feedback) and telephone mentorship (phone calls and texts on clinical questions, case reviews, and referrals). The frequency of mentor-mentee contacts ranged from weekly (during intervention month 1) to monthly (intervention months 3–6). Table 2 states: two 2-day low-dose simulation sessions, and high-frequency practice sessions using partographs and anatomic models.<br>2) <u>Provision of materials:</u> fetal doppler ultrasound scans, vacuum extractors, neonatal resuscitation equipment (bag and valve masks, penguin suction device), infection prevention and control equipment, national case-management guidelines, and simulators<br>• ROB: Low	3.0% [34/1118]; F/U I: 6.5% [76/1172]; BL C: 3.1% [40/1291]; F/U C: 3.1% [36/1151]; ES: 112.3 %-pts).<br>**From Table 4:**<br>Babies born with low APGAR scores requiring newborn resuscitation (BL I: 1.7% [18/1074]; F/U I: 3.7% [40/1094]; BL C: 0.3% [4/1247]; F/U C: 0.4% [4/1110]; ES: 105.8 %-pts).<br><br>**Health outcomes, from baseline to 10 months post-intervention (decreasing outcomes means improvement; see methods comment)**<br>Proportion of macerated stillbirths (BL I: 0.6% [7/1086]; F/U I: 0.6% [7/1102]; BL C: 0.4% [5/1272]; F/U C: 1.4% [16/1144]; ES: 257.3 %-pts); proportion of fresh stillbirths (BL I: 0.5% [5/1086]; F/U I: 0.1% [1/1102]; BL C: 1.6% [20/1272]; F/U C: 1.6% [18/1144]; ES: 80.4 %-pts); neonatal deaths (BL I: 0.5% [5/1074]; F/U I: 0.3% [3/1094]; BL C: 0.3% [4/1247]; F/U C: 0.1% [1/1110]; ES: -30.8 %-pts).		emergency intrapartum caesarean delivery), the ES is multiplied by –1.<br>• Among HCP practice outcomes, an increase means improvement. For all health outcomes, a decrease means improvement.<br>• Based on the article and personal communications with an author, an increase in the % of babies born with a low APGAR score means increased HCP performance because it shows that HCPs are getting better at identifying sick babies that need resuscitation.<br>• Some effect sizes are large because outcome events are rare, and the effect size formula uses relative changes. So, small absolute changes can lead to large relative changes (e.g., vacuum extraction deliveries increased from 0.18% to 2.46%, which is a relative increase of 1223%).
Kenya, Nigeria, South Africa, and Tanzania	Gallos et al. 2023; Forbes et al. 2024	• RCT with true control<br>• Health topic: Post-partum hemorrhage (PPH)<br>• Setting: Hospitals with 1000–5000 vaginal births/year and comprehensive obstetrical care with ability to perform surgery for postpartum hemorrhage<br>• Study does not call the intervention "LDHFT"<br>• Intervention (from Forbes 2024, pages 6-8 and Table 6):<br>1) Simulation, team-based on-site training with follow-up practice drills on the calibrated drape and MOTIVE bundle use to encourage staff to work as a team when detecting and managing a PPH (see comment). Staff participated in 2–7 simulation sessions.<br>2) Local champions to promote E-MOTIVE uptake and provide reminders and encouragement to staff implementing E-MOTIVE (see comment).<br>3) PPH trolley or carry case with all necessary drugs and equipment (see comment).<br>4) Audit and feedback (see comment).<br>• ROB: Low	**HCP practice outcomes ("key secondary implementation outcomes") from Table 2**<br>• Detection of PPH: C: 51.1%; I: 93.1%; adjusted difference ES: 33.3 %-pts<br>• Adherence to treatment bundle: C: 19.4%; I: 91.2%; adjusted difference ES: 70.2 %-pts<br><br>**Health outcome (primary) from Table 2**<br>Composite of severe postpartum hemorrhage (blood loss, ≥1000 ml), laparotomy for bleeding, or maternal death from bleeding: C: 4.3%; I: 1.6%; adjusted odds ratio: 0.40 (i.e., a 60% decrease, with a decrease being an improvement); ES: 60%.	**HCP practice outcome MES:** 51.8 %-pts<br><br>**Health outcome MES:** 60%	**Intervention details (Gallos 2023, Figure 1)**<br>• <u>Audit and feedback</u> meant newsletters to all staff with monthly rates of detection and bundle use, rates of PPH, severe PPH, blood transfusion, laparotomy, and death from PPH and giving feedback at monthly meetings<br>• <u>Champions</u> were 1 midwife and 1 doctor to oversee change, troubleshoot, give feedback on audit newsletters, connect with other champions via chats, meetings, and websites for sharing knowledge and lessons learned<br>• <u>Trolley or carry case.</u> Restocking of all medicines and devices used for treatment of PPH after every use and completion of a stocking checklist at the start of every shift<br>• <u>Training</u> was onsite, simulation-based, and peer-assisted training, lasting from 90 min to an entire workday, facilitated by HCP job aids displayed in labor wards

Country	Reference	Methods and risk of bias (ROB)	Results of LDHFT vs. controls (BL = baseline, C = control arm, ES = effect size, F/U = follow-up, I = intervention arm)	Median effect size (LDHFT vs. controls)	Comments
Kenya and Uganda	Walker et al. 2020; Namazzi et al. 2021	• RCT<br>• Health topic: Intrapartum and immediate newborn care for preterm and low-birthweight babies<br>• Setting: Facilities providing 24-h maternity care with at least 200 births/year (only 7 of 20 facilities could perform C-sections)<br>• Study does not call the intervention "LDHFT"<br>• Study arms (see comments):<br>1) Intervention: Facilities received maternity register data strengthening + a modified WHO Safe Childbirth Checklist + equipment for newborn care (if needed) + a series of PRONTO simulation and team training + quality improvement (QI) collaboratives.<br>2) Control: Facilities received maternity register data strengthening + a modified WHO Safe Childbirth Checklist + equipment for newborn care (if needed)<br>• ROB: Low	**HCP practice outcome from Table 2**<br>• Caesarean section rates (among 7 facilities that offered C-sections): C: 35.2%; I: 26.8%; aOR = 0.67 (i.e., a 33% decrease, with a decrease being an improvement); ES: 33%.<br>Note that baseline C-section values were not used because the reported results were median percentages among the 6 facilities that offered caesarean section. It is better to use final results from Table 2.<br><br>**Health outcome (primary) from Table 2**<br>• Combined % of fresh stillbirth and 28-day neonatal mortality among preterm and low-birthweight babies: C: 23.3%; I: 15.3%; adjusted odds ratio (aOR): 0.66 (i.e., a 34% decrease, with a decrease being an improvement); ES: 34%.	**HCP practice outcome MES:** 33%<br><br>**Health outcome MES:** 34%	**Intervention details**<br>• <u>Data strengthening</u> involved annual workshops + creation of a data dashboard with monthly reports<br>• <u>QI collaboratives</u> involved creation of QI teams that met every 2 weeks, tracked indicators, did plan-do-study-act cycles, and participated in learning sessions<br>• <u>PRONTO simulation and team training **in Kenya**</u> involved 4 consecutive days of training every 5-6 weeks (12 weeks of on-site training and mentorship per facility)<br>• <u>PRONTO simulation and team training **in Uganda**</u> involved 2 consecutive days of training every 6-8 weeks (total of 7 PRONTO trainings + 4 additional bedside mentorship visits)
Mali and Senegal	Dumont et al. 2013; Dumont et al. 2009; Zongo et al. 2015; Lalonde et al. 2006	• RCT with true-control group<br>• Health topic: maternal and perinatal health<br>• Setting: Hospitals with more than 800 deliveries/year and a functional operating room<br>• Study does not call the intervention "LDHFT"<br>• Intervention: 1) train opinion leaders in each intervention hospital with 6-day training course that included practicing clinical skills, 2) create and train a multidisciplinary audit committee in each intervention hospital, 3) launch the maternal mortality audit cycle with audit committee meetings every 2–3 months (which elicited quality improvement suggestions), 4) the local opinion leaders (supported by external facilitators) train clinicians in each intervention hospital with 4–8 training sessions (each lasted 0.5–1 day, spaced 1–3 months apart, and included practicing clinical skills), 5) educational outreach visits by external facilitators every 3 months, and 6) recertification training of the opinion leaders after 1 year that lasted 3 days and included practicing clinical skills.<br>• ROB: Moderate (contamination bias)	**HCP practice outcomes from Table 6 (see methods comment):** Blood transfusion in patients with haemorrhage (BL C: 43.36%; BL I: 44.48; F/U C: 45.66%; F/U I: 51.94%; ES: 11.47 %-pts); hysterectomy in patients with haemorrhage (BL C: 4.60%; BL I: 4.63; F/U C: 4.01%; F/U I: 4.80%; ES: 16.50 %-pts); transportation in patients with complication (BL C: 0.20%; BL I: 0.68; F/U C: 0.07%; F/U I: 0.39%; ES: 22.35 %-pts); elective caesarean delivery (BL C: 2.29%; BL I: 2.13; F/U C: 3.33%; F/U I: 3.25%; ES: 7.17 %-pts); emergency antepartum caesarean delivery (BL C: 2.32%; BL I: 3.76; F/U C: 2.07%; F/U I: 4.28%; ES: 24.61 %-pts); emergency intrapartum caesarean delivery (BL C: 13.26%; BL I: 15.92; F/U C: 17.62%; F/U I: 18.41%; ES: 17.24 %-pts); forceps/vacuum extraction (BL C: 2.15%; BL I: 2.26; F/U C: 2.11%; F/U I: 2.09%; ES: -5.66 %-pts).<br>Note that the following Table 6 HCP practice outcomes were not used because they had more broadly defined denominators that included women that would generally not need the action indicated by the outcome's numerator (see exclusion criteria in the methods section, above): 1) blood transfusion among all women, 2) hysterectomy among all women, and 3) transportation to another hospital among all women.<br><br>**Health outcome from Table 3**<br>• Hospital-based maternal mortality (the study's one primary outcome) ES: 15%	**HCP practice outcome MES:** 16.5 %-pts<br><br>**Health outcome MES:** 15%	• **Methods comment.** ES = % change over time in intervention arm minus % change over time in control arm = 100% x {[(intervention follow-up – intervention baseline)/intervention baseline] – [(control follow-up – control baseline)/control baseline]}. For the one outcome for which a decrease is an improvement (i.e., emergency intrapartum caesarean delivery), the ES is multiplied by –1.
Mexico	Fritz et al. 2017; Walker et al. 2014; Walker et al. 2016	• RCT with true-control group<br>• Health topic: obstetric and neonatal emergencies<br>• Setting: Hospitals with 500–3000 deliveries/year<br>• Study does not call the intervention "LDHFT"<br>• Intervention ("PRONTO"): two training modules (with simulations done in clinical settings) 2–3 months apart; and in addition to what occurred during the modules,	**HCP practice outcomes from Tables 2 and 3 in Fritz 2017 (baseline vs. 12-month follow-up):**<br><br>*Outcomes of recommended practices (a positive difference-in-differences means an improvement, and thus, a positive effect size):*<br>Complete active management of the 3rd stage of labor (AMTSL) (BL C: 17.0%; BL I: 18.2%; ES: 14.1 %-pts); first step of AMTSL (BL C: 27.3%; BL I:	**HCP practice outcome MES:** 11.9 %-pts<br><br>**Health outcome MES:** 32.4 %-pts	• **Methods comment 1.** For example, for episiotomy, the 12-month "impact" measure in Table 3 was –9.7 %-pts. As episiotomy is harmful, a negative value means an improvement in HCP performance (i.e., intervention caused fewer episiotomies). Therefore, the effect size in this rapid

Country	Reference	Methods and risk of bias (ROB)	Results of LDHFT vs. controls<br>(BL = baseline, C = control arm, ES = effect size, F/U = follow-up, I = intervention arm)	Median effect size (LDHFT vs. controls)	Comments
		there was some practice simulation (typically, every 1–3 months).<br>• ROB: Moderate (because randomization was not completely successful and because of some missing data)	32.1%; ES: 24.9 %-pts); skin-to-skin contact (BL C: 6.80%; BL I: 11.3%; ES: -2.2 %-pts); delayed umbilical cord clamping (BL C: 14.92%; BL I: 11.9%; ES: 41.9 %-pts).<br><br>*Outcomes of harmful practices (a negative difference-in-differences means an improvement, and thus, a positive effect size; the effect sizes below have already been multiplied by –1 to account for this interpretation, see methods comment 1):*<br>Episiotomy (BL C: 64.3%; BL I: 65.4%; ES: 9.7 %-pts); fundal pressure/Kristeller maneuver (BL C: 20.0%; BL I: 13.4%; ES: -3.6 %-pts); uterine sweeping (BL C: 80.6%; BL I: 85.2%; ES: 3.9 %-pts).<br><br>Another HCP practice outcome that does not neatly fit into either of the above categories, but that has a trend interpretation such that *a negative difference-in-differences means an improvement, and thus, a positive effect size; the effect size below has already been multiplied by –1 to account for this interpretation.*<br>**From Table 2 in Walker 2016 (baseline vs. 12-month follow-up, see methods comment 2):**<br>Cesarean delivery (BL C: 290.54; BL I: 333.80; F/U C: 361.45; F/U I: 324.98; ES: 27.05 %-pts).<br><br>**Health outcomes from Table 2 in Walker 2016 (baseline vs. 12-month follow-up, see methods comment 2):** Neonatal mortality (BL C: 10.00, BL I: 13.98; F/U C: 7.76; F/U I: 9.49; ES: 9.72 %-pts); obstetric hemorrhage (BL C: 8.90; BL I: 23.93; F/U C: 12.73; F/U I: 21.04; ES: 55.11 %-pts); hysterectomy (BL C: 5.22; BL I: 2.97; F/U C: 3.07; F/U I: 2.22; ES: -15.94 %-pts); eclampsia (BL C: 3.21; BL I: 3.81; F/U C: 3.49; F/U I: 1.57; ES: 67.52 %-pts).		review was multiplied by –1, so that the –9.7 %-pt value (now, +9.7 %-pts) can be interpreted the same as all other effect sizes.<br>• **Methods comment 2.** ES = % change over time in intervention arm minus % change over time in control arm = 100% x {[(intervention follow-up – intervention baseline)/intervention baseline] – [(control follow-up – control baseline)/control baseline]}. For outcomes for which a decrease is an improvement (e.g., mortality), the ES is multiplied by –1.<br>• A potential cause of relatively small effect sizes was that the PRONTO training focused on emergency care (relatively little time spent on normal births), yet the HCP performance outcomes all related to normal births.
Mexico	Castro-Ríos et al. 2008; Pérez-Cuevas et al. 2000	• Before-and-after study with non-randomized true controls<br>• Health topic: diabetes<br>• Setting: Family medicine clinics<br>• Study does not call the intervention LDHFT<br>• Intervention: 1) "first stage" was five 1-hour on-site interactive sessions to discuss diabetes guidelines, 2) "second stage" was three to five 2-hour individual on-site sessions (once a week) with one HCP and a clinical tutor while the HCP was treating patients to promote use of the guidelines, and 3) "stage 3" was three 1-hour weekly round-table peer-review sessions (each with a clinical tutor and 3–4 HCPs) to discuss clinical cases. The entire intervention was delivered over 2–3 months.<br>• ROB: High (because of non-randomized design)<br>• From HCPPR database, COMP_IDnew = 29610000112	**HCP practice outcome from HCPPR database (Excel file "**All effect sizes (from ph1_2_es2May2020)**")**<br>• Probability of an appropriate prescription of hypoglycemic drugs (range 0-1, scaled to 0-100%). ES: 0.6 %-pts	**HCP practice outcome MES:** 0.6 %-pts	

Country	Reference	Methods and risk of bias (ROB)	Results of LDHFT vs. controls (BL = baseline, C = control arm, ES = effect size, F/U = follow-up, I = intervention arm)	Median effect size (LDHFT vs. controls)	Comments
Mexico	Reyes-Morales et al. 2009; Pérez-Cuevas et al. 2000	• Before-and-after study with non-randomized true controls<br>• Health topic: acute respiratory infections (ARI)<br>• Setting: Family medicine clinics<br>• Study does not call the intervention LDHFT<br>• Intervention: 1) "first stage" was five 1-hour on-site interactive sessions to discuss ARI guidelines, 2) "second stage" was three 2-hour individual on-site sessions (about 3 weeks apart) with one HCP and a clinical tutor while the HCP was treating patients to promote use of the ARI guidelines, and 3) "stage 3" was three 1-hour weekly round-table peer-review sessions (each with a clinical tutor and 3–4 HCPs) to discuss clinical cases. The entire intervention was delivered over 3 months.<br>• ROB: High (because of non-randomized design)<br>• From HCPPR database, COMP_IDnew = 27879010312	**HCP practice outcome from HCPPR database (Excel file "**All effect sizes (from ph1_2_es2May2020)**")**<br>• Mean % of patients with appropriate case management per HCP. BL C: 9.6%; F/U C: 6.9%; BL I: 4.7%; F/U I: 24.3%; ES: 22.3 %-pts	**HCP practice outcome MES:** 22.3 %-pts	
Nigeria	Odusanya et al. 2004	• RCT with true controls<br>• Health topic: rational medicine use<br>• Setting: Outpatient health facilities<br>• Study does not call the intervention LDHFT<br>• Intervention: Weekly on-site training (for 4 weeks) for all HCPs (duration not stated, but with training in multiple facilities, it was almost certainly a day or less) that included clinical practice.<br>• ROB: HIgh (because only two units of randomization)<br>• From HCPPR database, COMP_IDnew = 14420000112	**HCP practice outcomes from HCPPR database (Excel file "**All effect sizes (from ph1_2_es2May2020)**")**<br>• % of patients managed according to standing orders (complimentary indicator or rational management). ES: 7.2 %-pts<br>• Average number of drugs per encounter (Decrease = improvement). ES: 23.1 %-pts	**HCP practice outcome MES:** 15.2 %-pts	
Pakistan	Kumar et al. 2015	• Before-and-after study with non-randomized true controls<br>• Health topic: infectious waste management<br>• Setting: Hospitals<br>• Study does not call the intervention LDHFT<br>• Intervention: 1) three on-site trainings (6 hours each) for all HCPs with hands-on practice on use of personal protective equipment (PPE) and steps in infectious waste management, 2) provision of PPE, and 3) reminders on infectious waste management given by administrators during weekly meetings.<br>• ROB: Very high (because of non-randomized design and self-reported behaviors)<br>• From HCPPR database, COMP_IDnew = 27370000112	**HCP practice outcomes from HCPPR database (Excel file "**All effect sizes (from ph1_2_es2May2020)**")**<br>• Mean % practices score on infectious waste management per HCP (range 0-20, scaled 0-100%). ES: 17.5 %-pts	**HCP practice outcome MES:** 17.5 %-pts	
South Africa	van Zyl et al. 2004	• Pre-post study with non-randomized control<br>• Health topic: outpatient diabetes management<br>• Setting: Two outpatient clinics<br>• Study does not call the intervention LDHFT	**HCP practice outcomes from Table II (see methods comment 1):** Mean process measure score (ranging from 0 to 6): BL I: 1.74; F/U I: 4.60; BL C: 1.74; F/U C: 2.04. These values were converted into percentages by dividing	**HCP practice outcome MES:** 42.7 %-pts	• **Methods comment 1.** ES = (Intervention F/U – Intervention BL) – (Control F/U – Control BL).

Country	Reference	Methods and risk of bias (ROB)	Results of LDHFT vs. controls (BL = baseline, C = control arm, ES = effect size, F/U = follow-up, I = intervention arm)	Median effect size (LDHFT vs. controls)	Comments
		• Intervention: Quarterly on-site training for all doctors working in the intervention clinic on diabetes management (duration seemed to be about one-half day) that was linked to a structured consultation with a patient that allowed HCPs to practice what they learned during the training.<br>• ROB: High (because of non-randomized design)<br>• From HCPPR database, COMP_IDnew = 6190000112	by 6 and multiplying by 100%: BL I: 29.0%; F/U I: 76.7%; BL C: 29.0%; F/U C: 34.0%; ES: 42.7 %-pts.<br>Note that this score includes all six individual process indicators in Table II, and so these individual indicators are not used (to avoid double-counting).<br><br>**Health outcomes:**<br>**From Table III (see methods comment 2):** HbA1c (note that a decrease is an improvement) (BL I: 9.77%; F/U I: 8.481%; BL C: 10.27%; F/U C: 9.153%; ES: 2.3 %-pts).<br><br>Note that the indicator "percentage of patients with good glycemic control" was not used because it is simply a re-analysis of the continuous HbA1c described above.	**Health outcome MES:** 2.3 %-pts	• **Methods comment 2.** ES = % change over time in intervention arm minus % change over time in control arm = 100% x {[(intervention follow-up – intervention baseline)/intervention baseline] – [(control follow-up – control baseline)/control baseline]}. For outcomes for which a decrease is an improvement (e.g., mortality), the ES is multiplied by –1.<br>• Note that the outcome on consultation time was not used because baseline values were not reported, and this was a non-randomized study.
Tanzania	Alwy Al-beity et al. 2019; Hanson et al. 2017	• RCT with a true control group<br>• Health topic: post-partum hemorrhage<br>• Setting: District clusters with a district hospital and 2–3 large health centers with at least 3000 deliveries/year<br>• Study does call the intervention LDHFT<br>• Intervention: 1-day simulation-based in-facility training (Helping Mothers Survive Bleeding after Birth [HMS BAB]) is followed by weekly practice sessions for 6–8 weeks (each lasting 30–40 minutes), supported by peer practice coordinators from each facility (who got an extra half-day training and weekly phone calls to remind them to initiate the practice sessions). Note that HMS BAB includes on-site training, the provision of materials for practicing simulations in labor rooms, and monitoring QI through ongoing data collection and feedback analysis reports.<br>• ROB: Low	**HCP practice outcomes from Figure 5 (see methods comment 1):** AMTSL among women with PPH (BL C: 83.4%; BL I: 87.3%; F/U C: 95.8%; F/U I: 92.5%; ES: -7.2 %-pts); iv oxytocin among women with PPH (BL C: 100%; BL I: 96.0%; F/U C: 89.5%; F/U I: 93.0%; ES: 7.5 %-pts).<br><br>**Health outcomes from Figure 4 (see methods comment 2):** All-cause near misses among all deliveries in a facility (BL C: 6.6%; BL I: 7.2%; F/U C: 5.5%; F/U I: 5.2%; ES: 11.1 %-pts); PPH near misses among all deliveries in a facility (BL C: 1.5%; BL I: 2.9%; F/U C: 1.6%; F/U I: 1.3%; ES: 61.8 %-pts); PPH near misses among women with PPH during health facility deliveries (BL C: 61.3%; BL I: 86.1%; F/U C: 72.7%; F/U I: 65.8%; ES: 42.2 %-pts).	**HCP practice outcome MES:** 0.2 %-pts<br><br>**Health outcome MES:** 42.2 %-pts	• **Methods comment 1.** ES = (Intervention F/U – Intervention BL) – (Control F/U – Control BL).<br>• **Methods comment 2.** ES = % change over time in intervention arm minus % change over time in control arm = 100% x {[(intervention follow-up – intervention baseline)/intervention baseline] – [(control follow-up – control baseline)/control baseline]}. For outcomes for which a decrease is an improvement (e.g., mortality), the ES is multiplied by –1.

Country	Reference	Methods and risk of bias (ROB)	Results of LDHFT vs. controls<br>(BL = baseline, C = control arm, ES = effect size, F/U = follow-up, I = intervention arm)	Median effect size (LDHFT vs. controls)	Comments
Uganda	Hanson et al., 2021;<br>Hanson et al. 2017	• RCT with a true control group<br>• Health topic: post-partum hemorrhage<br>• Setting: District clusters with a district hospital and 2–3 large health centers with at least 4000 deliveries/year<br>• Study does call the intervention LDHFT<br>• Intervention: 1-day simulation-based in-facility training (HMS BAB) is followed by weekly practice sessions for 6–8 weeks (each lasting 15–20 minutes), supported by peer practice coordinators from each facility (who got an extra half-day training and weekly phone calls for 3 weeks to remind them to initiate the practice sessions). Note that HMS BAB includes on-site training, the provision of materials for practicing simulations in labor rooms, and monitoring QI through ongoing data collection and feedback analysis reports.<br>• ROB: Low	**HCP practice outcomes from graphs provided by author (see methods comment 1):** AMTSL in women experiencing PPH (BL C: 92.3%; BL I: 90.7%; F/U C: 91.2%; F/U I: 90.7%; ES: 1.1 %-pts); Women treated with IV oxytocin in women experiencing PPH (BL C: 76.1%; BL I: 93.4%; F/U C: 89.8%; F/U I: 90.0%; ES: -17.1 %-pts).<br><br>**Health outcomes from graphs provided by author (see methods comment 2):** Near misses among all women who delivered in a facility (BL C: 10.2%; BL I: 4.6%; F/U C: 5.8%; F/U I: 4.4%; ES: -38.8 %-pts); PPH near misses among all women who delivered in a facility (BL C: 3.8%; BL I: 1.8%; F/U C: 2.4%; F/U I: 1.7%; ES: -31.3 %-pts); PPH near misses among women who suffered PPH during health facility delivery (BL C: 62.5%; BL I: 68.8%; F/U C: 95.7%; F/U I: 90.3%; ES: 21.9 %-pts).	**HCP practice outcome MES:** –8.0 %-pts<br><br>**Health outcomes MES:** –31.1 %-pts	• **Methods comment 1.** ES = (Intervention F/U – Intervention BL) – (Control F/U – Control BL).<br>• **Methods comment 2.** ES = % change over time in intervention arm minus % change over time in control arm = 100% x {[(intervention follow-up – intervention baseline)/intervention baseline] – [(control follow-up – control baseline)/control baseline]}. For outcomes for which a decrease is an improvement (e.g., mortality), the ES is multiplied by –1.

REFERENCES

Alwy Al-beity F, Pembe A, Hirose A, et al. Effect of the competency-based Helping Mothers Survive Bleeding after Birth (HMS BAB) training on maternal morbidity: A cluster-randomised trial in 20 districts in Tanzania. *BMJ Glob Health*. 2019;4:e001214.

Asiedu A, Nelson AR, Gomez PP, Tappis H, Effah F, Allen C. "It builds your confidence… you've done well": Healthcare workers' experiences of participating in a low-dose, high-frequency training to improve newborn survival on the day of birth in Ghana. *Gates Open Research* 2019;3:1470.

Castro-Ríos A, Reyes-Morales H, Pérez-Cuevas R. [An evaluation of a continuing medical education program for primary care services in the prescription of hypoglycemic agents in diabetes mellitus type 2]. *Salud Pública de México*. 2008;50 Suppl 4:S445–52. Spanish.

Chaulagain DR, K C A, Wrammert J, Brunell O, Basnet O, Malqvist M. Effect of a scaled-up quality improvement intervention on health workers' competence on neonatal resuscitation in simulated settings in public hospitals: A pre-post study in Nepal. *PLoS One.* 2021;16:e0250762.

Chaulagain DR, Malqvist M, Brunell O, Wrammert J, Basnet O, Kc A. Performance of health workers on neonatal resuscitation care following scaled-up quality improvement interventions in public hospitals of Nepal - a prospective observational study. *BMC Health Serv Res.* 2021;21:362.

Dumont A, Fournier P, Abrahamowicz M, Traore M, Haddad S, Fraser WD. Quality of care, risk management, and technology in obstetrics to reduce hospital-based maternal mortality in Senegal and Mali (QUARITE): A cluster-randomised trial. *Lancet.* 2013;382:146–157. Personal communication from Alexandre Dumont, Institut National de Recherche pour le Développement (IRD), Paris, on December 16, 2024.

Dumont A, Fournier P, Fraser W, et al. QUARITE (quality of care, risk management and technology in obstetrics): a cluster randomized trial of a multifaceted intervention to improve emergency obstetric care in Senegal and Mali. *Trials.* 2009;10:85.

Forbes G, Akter S, Miller S, Galadanci H, Qureshi Z, Al-Beity FA, et al. Development and piloting of implementation strategies to support delivery of a clinical intervention for postpartum hemorrhage in four sub-Saharan Africa countries. *Glob Health Sci Pract*. 2024;12(5):e2300387.

Fransen AF, van de Ven J, Banga FR, Mol BWJ, Oei SG. Multi-professional simulation-based team training in obstetric emergencies for improving patient outcomes and trainees' performance. *Cochrane Database Syst Rev.* 2020; 2020(12).

Fritz J, Walker DM, Cohen S, Angeles G, Lamadrid-Figueroa H. Can a simulation-based training program impact the use of evidence based routine practices at birth? Results of a hospital-based cluster randomized trial in Mexico. *PLOS One.* 2017;12(3):e0172623. Personal communications from Jimena Fritz, National Institute of Public Health (INSP), Mexico, on December 10 and 12, 2024.

Gallos I, Devall A, Martin J, Middleton L, Beeson L, Galadanci H, et al. Randomized Trial of Early Detection and Treatment of Postpartum Hemorrhage. *N Engl J Med*. 2023;389(1):11–21.

Gomez PP, Nelson AR, Asiedu A, et al. Accelerating newborn survival in Ghana through a low-dose, high-frequency health worker training approach: a cluster randomized trial. *BMC Pregnancy Childbirth* 2018;18,72. Personal communications from Patricia Gomez, Jhpiego, on December 16, 2024, and from Amos Asidue, Jhpiego, on January 21, 2025.

Hanson C, Atuhairwe S, Lucy Atim J, Marrone G, Morris JL, Kaharuza F. Effects of the Helping Mothers Survive Bleeding after Birth training on near miss morbidity and mortality in Uganda: A cluster-randomized trial. *Int J Gynecol Obstet* 2021;152(3):386–394. Personal communication from Claudia Hanson, Karolinska Institutet, Stockholm, on January 19, 2025.

Hanson C, Pembe AB, Alwy F, et al. Evaluating the effect of the Helping Mothers Survive Bleeding after Birth (HMS BAB) training in Tanzania and Uganda: Study protocol for a randomised controlled trial. *Trials.* 2017;18:307.

Jhpiego. Low Dose, High Frequency: A Learning Approach to Improve Health Workforce Competence, Confidence, and Performance. 2016. Accessed December 5, 2024. https://hms.jhpiego.org/wp-content/uploads/2016/08/LDHF_briefer.pdf

Kc A, Ewald U, Basnet O, et al. Effect of a scaled-up neonatal resuscitation quality improvement package on intrapartum-related mortality in Nepal: a stepped-wedge cluster randomized controlled trial. *PLoS Med*. 2019;16:e1002900.

Kirkpatrick DL, Kirkpatrick JD. Evaluating Training Programs: The Four Levels. Berrett-Koehlar; 1994.

Kruk ME, Gage AD, Arsenault C, et al. High-quality health systems in the Sustainable Development Goals era: time for a revolution. *Lancet Glob Health* 2018 Nov;6(11):e1196–e1252.

Kumar R, Somrongthong R, Shaikh BT. Effectiveness of intensive healthcare waste management training model among health professionals at teaching hospitals of Pakistan: a quasi-experimental study. *BMC Health Serv Res*. 2015;15:81.

Lalonde AB, Beaudoin F, Smith J, Plourde S, Perron L. The ALARM International Program: a mobilizing and capacity-building tool to reduce maternal and newborn mortality and morbidity worldwide. *J Obstet Gynaecol Can* 2006;28(11):1004–8.

Mukeshimana M, Bigirimana R, Kalisa R, Mwali AK, Radeny S. Enhancing provider competencies through the low-dose, high-frequency training approach. IntraHealth International. May 2022. Accessed December 5, 2024. https://www.intrahealth.org/sites/default/files/attachment-files/tb4enhancingprovidercompetenciesrev-5-22hr-d.pdf

Namazzi G, Achola KA, Jenny A, Santos N, Butrick E, Otieno P, Waiswa P, Walker D; Preterm Birth Initiative Kenya & Uganda Implementation Research Collaborative. Implementing an intrapartum package of interventions to improve quality of care to reduce the burden of preterm birth in Kenya and Uganda. *Implement Sci Commun.* 2021;2(1):10.

Odusanya OO, Oyediran MA. The effect of an educational intervention on improving rational drug use. *Niger Postgrad Med J*. 2004;11(2):126–131.

Pérez-Cuevas R, Reyes H, Guiscafré H, Juárez-Díaz N, Oviedo M, Flores S, et al. The primary care clinic as a setting for continuing medical education: program description. *CMAJ.* 2000;14;163(10):1295–9.

Reyes-Morales H, Flores-Hernández S, Tomé-Sandoval P, Pérez-Cuevas R. A multifaceted education intervention for improving family physicians' case management. *Fam Med*. 2009;41(4):277–84. Personal communications from Hortensia Reyes-Morales, Instituto Nacional de Salud Pública, Cuernavaca, Mexico, on January 17 and 18, 2025.

Rowe AK, Rowe SY, Peters DH, Holloway KA, Chalker J, Ross-Degnan D. The effectiveness of strategies to improve health worker knowledge in low- and middle-income countries and the association between knowledge and clinical practice: a systematic review. Poster presentation by AK Rowe at the 66th Annual Meeting of the ASTMH, November 5–9, 2017, Baltimore, Maryland. Abstract 233.

Rowe SY, Peters DH, Holloway KA, Chalker J, Ross-Degnan D, Rowe AK. A systematic review of the effectiveness of strategies to improve health care provider performance in low- and middle-income countries: Methods and descriptive results. *PLoS One.* 2019;14(5):e0217617.

Shikuku DN, Mukosa R, Peru T, Yaite A, Ambuchi J, Sisimwo K. Reducing intrapartum fetal deaths through low-dose high frequency clinical mentorship in a rural hospital in Western Kenya: a quasi-experimental study. *BMC Pregnancy Childbirth.* 2019;19(1):518. Personal communication from Duncan Shikuku, Liverpool School of Tropical Medicine, Nairobi, on December 20 and 21, 2024.

Sutton RM, Niles D, Meaney PA, et al. Low-dose high-frequency CPR training improves skill retention of in-hospital pediatric providers. *Pediatrics*. 2011;128:e145–e151.

Ugwa E, Kabue M, Otolorin E, et al. Simulation-based low-dose, high-frequency plus mobile mentoring versus traditional group-based trainings among health workers on day of birth care in Nigeria; a cluster randomized controlled trial. *BMC Health Serv Res*. 2020;20:586.

van Tetering AAC, Ntuyo P, Martens RPJ, Winter N, Byamugisha J, Oei SG, et al. Simulation-based training in emergency obstetric care in Sub-Saharan and Central Africa: A Scoping Review. *Ann Glob Health*. 2023;89(1):62.

van Zyl DG, Rheeder P. Physician education programme improves quality of diabetes care. *S Afr Med J*. 2004;94(6):455–459.

Walker D, Cohen S, Fritz J, Olvera M, Lamadrid-Figueroa H, Cowan JG, Hernandez DG, Dettinger JC, Fahey JO. Team training in obstetric and neonatal emergencies using highly realistic simulation in Mexico: impact on process indicators. *BMC Pregnancy Childbirth.* 2014;14:367.

Walker DM, Cohen SR, Fritz J, Olvera-García M, Zelek ST, Fahey JO, Romero-Martínez M, Montoya-Rodríguez A, Lamadrid-Figueroa H. Impact Evaluation of PRONTO Mexico: A Simulation-Based Program in Obstetric and Neonatal Emergencies and Team Training. *Simul Healthc.* 2016;11(1):1-9. Erratum in: *Simul Healthc*. 2017;12(1):65.

Walker D, Otieno P, Butrick E, et al. Effect of a quality improvement package for intrapartum and immediate newborn care on fresh stillbirth and neonatal mortality among preterm and low-birthweight babies in Kenya and Uganda: A cluster-randomised facility-based trial. *Lancet Glob Heal*. 2020; 8(8): e1061–e1070. Personal communication from Dilys Walker, Institute for Global Health Sciences, University of California San Francisco, on December 17, 2024.

Willcox M, Harrison H, Asiedu A, Nelson A, Gomez P, LeFevre A. Incremental cost and cost-effectiveness of low-dose, high-frequency training in basic emergency obstetric and newborn care as compared to status quo: part of a cluster-randomized training intervention evaluation in Ghana. *Globalization and Health.* 2017;13:88.

Zhang CH, Hsu L, Zou BR, Li JF, Wang HY, Huang J. Effects of a pain education program on nurses' pain knowledge, attitudes and pain assessment practices in China. *J Pain Symptom Manage*. 2008;36(6):616–27.

Zongo A, Dumont A, Fournier P, Traore M, Kouanda S, Sondo B. Effect of maternal death reviews and training on maternal mortality among cesarean delivery: Post-hoc analysis of a cluster-randomized controlled trial. *Eur J Obstet Gynecol Reprod Bio.* 2015;185:174–180.

Text S8. Information and communication technology

A rapid review of information and communication technology as a stand-alone intervention to improve healthcare provider performance in low- and middle-income countries

OBJECTIVE

Characterize the effect of information and communication technology (ICT) (i.e., digital interventions) for healthcare providers (HCPs) and their supervisors on HCP practices in low- and middle-income countries (LMICs).

METHODS

- Definition of ICT. This rapid review only examined ICT for HCPs and their supervisors. Studies of ICT that was only for clients (e.g., appointment reminders sent to a client's phone) were excluded. ICT categories included (but were not limited to) applications on mobile phones or computers for clinical decision-making support, text-message reminders on case management, tracking HCP performance, counseling support, supply management, disease surveillance reporting, and e-learning. Note that ICT interventions are sometimes accompanied by other intervention components, such as training. In such cases, the other (non-ICT) intervention components are recorded so that the analysis can differentiate between evaluations of ICT as a stand-alone intervention versus ICT plus other components. The provision of equipment needed for the ICT intervention and training on how to use the ICT (including basic training materials, such as manuals and job aids) were considered to be part of ICT (i.e., not separate interventions).

- Definition of HCP. Health workers that provide health care to patients or that perform other health-related activities (e.g., educating patients or community members) in a hospital, outpatient clinic, or community setting. For this rapid review, supervisors of front-line HCPs were also included, as supervision is a health-related activity.

- Inclusion criteria. Eligible studies: 1) were done in an LMIC, 2) examined the quantitative effect of ICT for HCPs and their supervisors on an eligible outcome, and 3) used an eligible study design. Eligible outcomes were: 1) HCP practices or processes of care (which reflect behaviors when managing real patients or simulated clients that HCPs believe are real patients), such as correct patient assessment, diagnosis, treatment, counseling, referral, preventive services, and documentation; and 2) outcomes related to supervision (e.g., percentage of HCPs who received supervision, or percentage of supervisory visits in which a checklist was used). Eligible study designs were randomized-controlled trials (RCTs). Eligible effect sizes were based on: 1) a comparison of an ICT intervention arm and a no-intervention control arm, 2) a head-to-head study design that compared an ICT intervention versus a similar non-ICT intervention (e.g., an ICT guideline versus paper guidelines only, or e-learning versus in-person learning), and 3) a head-to-head study design that compared two interventions and the difference between the two interventions was ICT (i.e., to evaluate the marginal effect of ICT given the presence of other intervention components) (e.g., ICT + training versus training only).

- Exclusion criteria. 1) Outcomes of "inputs" or "structural quality" (e.g., availability of medicines), 2) HCP knowledge outcomes, 3) HCP skill outcomes measured during simulations (i.e., not HCP practice outcomes, which are behaviors when managing patients), 4) outcomes of community-level coverage that included all community members regardless of whether they had sought care from HCPs (e.g., proportion of all newborns that received polio vaccination, as measured by a household survey), 5) studies that only examined the effect of ICT for patients (e.g., messages sent to a patient's phone as reminders for a medical appointment or to take medicines), 6) outcomes that are related to already-used outcomes (e.g., in Zakus et al. [2019], in which the primary outcome was a quality of care score composed of diagnostic and treatment variables, the individual diagnostic and treatment variables were not be eligible because they are already part of the quality of care score), and 7) HCP practice outcomes with a baseline or control value that is $\geq$95% (i.e., no performance problem existed, very little room to improve, and so not a good context to evaluate any intervention). Outcomes of inputs and HCP knowledge were excluded because they are only weakly correlated with HCP practices [Kruk et al. 2018; Rowe et al. 2017]. Outcomes of HCP skills measured during simulations were excluded because it is unclear how well these outcomes reflect behaviors with real patients. Outcomes of community coverage that included all community members regardless of whether they had sought care from HCPs were excluded because achieving these outcomes requires a combination of care-seeking by community members and correct HCP practices.

- Literature search. This rapid review was designed to supplement a formal systematic review (the Health Care Provider Performance Review, or HCPPR [Rowe 2019]) that performed an in-depth literature search up through 2016. The HCPPR identified eight study comparisons of ICT (five for professional HCPs and 3 for lay HCPs). Regarding the literature search for this supplementary rapid review, there were three components. First, bibliographies of eight reviews and overviews were hand-searched [Agarwal 2016; Ahuja 2023; Aryee 2024; Bernasconi 2024; Deussom 2022; Keitel 2018; USAID 2023; WHO 2019]. Second, a reference list on digital learning platforms was provided by an ICT expert (Naina Ahuja, UNICEF) on February 10, 2025. The reference list contained grey literature and articles published in academic journals, and was updated through late 2021. Third, searches on PubMed were performed on February 8, 2025, using the terms: e-learning, information and communication technology, and electronic clinical decision support system; and randomized controlled trials; and low- and middle-income countries. The searches were restricted to the past 5 years (2020–2025). The searches yielded 51 citations and no new studies. However, the searches did identify two new systematic reviews (Cockburn 2024; Zarekar 2025); and when their bibliographies were hand-searched, one new eligible trial was found.

- Data abstraction. Effect sizes (or outcome values needed to calculate effect sizes) from eligible outcomes were abstracted from the original study reports, which are listed in the tables below. Effect sizes were abstracted by one person (AKR).

- Risk of bias (ROB) assessment. The ROB was based on the study design and study limitations: low risk for RCTs with zero or one minor limitation; moderate risk for RCTs with one main limitation or two minor limitations; high risk for RCTs with two main limitations;

and very high risk for studies with more limitations than those listed for the high ROB level. Note that RCTs with only one facility or cluster per study arm were categorized as high ROB.

- Analysis. Effect sizes were measured in terms of absolute percentage-point (%-pt) improvements. For outcomes measured as a percentage that could plausibly change over the entire range from 0 to 100% (i.e., most HCP practice outcomes, such as percentage of children weighed), the effect size = (follow-up – baseline)$_{intervention}$ – (follow-up – baseline)$_{controls}$. For continuous unbounded outcomes (e.g., number of medicines prescribed) and for percentage outcomes that are only expected to change over a narrow range (e.g., percentage of children urgently referred), the effect size = 100% x {[(follow-up – baseline)/baseline]$_{intervention}$ – [(follow-up – baseline)/baseline]$_{controls}$}. Often, studies did not present effect sizes calculated this way, and the reports did not include baseline and follow-up outcome values that could be used to calculate effect sizes. Instead, studies reported similar kinds of effect sizes (e.g., difference-in-differences effects, or odds ratios for health outcomes) that were estimated with statistical regression models that adjusted for potential confounders and clustering. When available, these model-based effect size estimates were used. For each study comparison, the median of effect sizes (MES) from HCP practice outcomes was calculated. The distribution of the study-comparison-specific MES values was described with a median and interquartile range. MES distributions are presented separately for professional HCPs (e.g., physicians, nurses, and midwives) versus lay HCPs (e.g., community health workers [CHWs]). Additionally, MES distributions are presented separately for superiority trials (i.e., trials designed to prove that a new intervention was more effective than a control group or other comparison intervention) and equivalence or non-inferiority trials (i.e., trials designed to prove that a new intervention was as effective as a comparison intervention). All analyses included studies identified by the HCPPR and newer studies from this supplementary rapid review. The main analysis included studies with all ROB levels, and a sensitivity analysis only included studies with low or moderate ROB. A special analysis was performed for studies of ICT on supervision outcomes.

- Supplemental information from study investigators. When there were questions about measurement methodology, the interpretation of results for a particular study, or data from unpublished studies, an attempt was made to contact the study investigators for information. These contacts are listed as personal communications in the tables and reference list below.

RESULTS

- The supplementary rapid review included 17 study comparisons (and thus 17 MES values) from 14 LMICs (**Table S5** in the **Online Supplementary Document**, below). All articles were in English. Forty-one studies were excluded (details available on request), mostly because of ineligible study designs (the large majority of which would have been ineligible for the HCPPR—e.g., uncontrolled pre-post studies).

- When studies included in the rapid review were combined with the older HCPPR results (8 study comparisons, see **Table S6** in the **Online Supplementary Document**, below), the total was 25 study comparisons from 15 LMICs. Thirteen study comparisons had a low ROB, 12

had a moderate ROB, zero had a high ROB, and zero had a very high ROB. Eleven study comparisons tested ICT for professional HCPs, and 14 study comparisons tested ICT for lay HCPs (and sometimes their supervisors). Twenty-four study comparisons were superiority trials, and one was an equivalency trial.

- *Main and sensitivity analyses of stand-alone ICT on HCP practice outcomes*
  - For the main analysis of superiority trials (including all ROB levels), for professional HCPs, the median MES was 6.5 %-pts (interquartile range: –2.4 to 15.8 %-pts, N = 11 study comparisons). Overall, the quality of evidence was high. For lay HCPs (i.e., CHWs), the median MES was 6.7 %-pts (interquartile range: 2.9 to 12.4 %-pts, N = 13 study comparisons). Overall, the quality of evidence was high. For the sensitivity analysis (with only low or moderate ROB levels), the results were identical to those of the main analysis.
  - The one equivalency trial (Rahman et al. 2019) studied an ICT intervention arm (e-learning for CHWs plus distance supervision of CHW supervisors) versus a non-ICT comparison arm (in-person training plus in-person supervision of CHW supervisors). CHWs in both arms delivered mental health care to women in the community using a paper-based manual. The effect size was close to zero (–1.6 %-pts), which meant that the ICT intervention was approximately as effective as the in-person intervention.

- *Special analysis that only examined ICT for HCP supervisor outcomes*
  - Among the four included study comparisons (including all ROB levels), the median MES was 3.7 %-pts (interquartile range: 0.9 to 6.6 %-pts). Overall, the quality of evidence was high. All studies involved supervisors of lay HCPs (i.e., CHWs), and all were superiority trials. For the sensitivity analysis (with only low or moderate ROB levels), the results were identical to those of the main analysis.

Table S5. The effect of information and communication technology (ICT) (i.e., digital interventions) for healthcare providers (HCPs) and their supervisors on HCP practice outcomes in low- and middle-income countries (LMICs). Supplementary studies from the rapid review.

Country	Reference	Methods and risk of bias (ROB)	Results of ICT vs. comparison group (BL = baseline, C = control arm, ES = effect size, F/U = follow-up, I = intervention arm)	Median effect size (ICT vs. comparison)	Comments
Burkina Faso	Sarrassat et al. 2021	• RCT of intervention (ICT clinical support system) vs. comparison (standard Integrated Management of Childhood Illness [IMCI] with paper forms)<br>• Setting: Primary health facilities caring for sick children with IMCI<br>• Intervention: Electronic Clinical Decision Support System (eCDSS) (on tablets) to help HCPs adhere to IMCI guidelines<br>• ROB: Moderate (because of contamination—see comment)	**HCP practice outcomes.**<br>**The primary outcomes (from Table 4) were:**<br>• Overall adherence to clinical assessment tasks, ES: 29.9 %-pts.<br>• Overall correct classification ignoring the severity of the classifications, ES: 10.1 %-pts.<br>• Overall correct prescription according to HCWs' classifications, ES: -1.1 %-pts.<br><br>Note that effect sizes were from models and labeled as "cluster-level mean difference between arms".	**HCP practice outcome MES:** 10.1 %-pts	**Interpretation of results.** Study wording does not suggest that this was an equivalency trial (wording indicates that it was a superiority trial).<br><br>**Contamination concern (from page 3).** "Full implementation in a district was…when the eCDSS was provided to all primary facilities and when all HCWs had been trained in its use and IMCI guidelines. In some control districts, data were collected after implementation started but before the full implementation was completed, resulting in some "contamination" of these control districts."
China	Chen et al. 2014	• RCT of intervention (text messages on guidelines) vs. comparison (traditional 1-day training)<br>• Setting: Physicians, nurses, midwives, and other health workers who had a mobile phone in remotely located health centers<br>• Intervention: 16 text messages<br>• ROB: Low	**HCP practice outcomes from Table 3.** These were secondary outcomes.<br>• % of patients with an upper respiratory tract infection (URI) who were prescribed an antibiotic. BL I: 68%; F/U I 68%; BL C: 50%; F/U C: 67%; ES: 17 %-pts.<br>• % of patients with a URI who were prescribed a steroid. BL I: 24%; F/U I 19%; BL C: 6%; F/U C: 17%; ES: 16 %-pts.<br><br>Note that a decrease in prescriptions was considered an improvement, as the study was about improving quality of care for URIs, which typically do not need these drugs. Thus, ES = difference in differences x negative 1.	**HCP practice outcome MES:** 16.5 %-pts	**Interpretation of results.** Study wording does not suggest that this was an equivalency trial (wording indicates that it was a superiority trial).<br><br>**Primary outcome was HCP knowledge from Table 2.** For all HCPs (cluster level analysis), BL I: 0.32; F/U I: 0.47; BL C: 0.33; F/U C: 0.32. However, it was unclear whether these "scores" were percentages on a scale of 0 to 100 or proportions on a scale of 0 to 1.<br><br>**Cost outcome.** Total cost on text messages for the intervention group was US$ 0.32 per HCP. In comparison, the one-day training for the control group cost US$ 89.96 per HCP (including printed materials, accommodation, and transportation)—i.e., a 280-fold difference per HCP.
India	Carmichael et al. 2019 [ICT supervision]	• RCT (intervention arm vs. true control arm)<br>• Setting: CHWs providing PHC services<br>• Intervention: A feature phone with a keyboard provided to each CHW, with functions to assist with registering beneficiaries, scheduling home visits, provision of health information through videos, guided protocols for conducting home visits, tracking child immunizations, and supervisory tools.<br>• ROB: Low	**Primary outcomes were reproductive, maternal, newborn, child health and nutrition services (RMNCHN) behaviors from Table 4.**<br>*Antenatal care.* At least 3 antenatal home visits (%) (DiD ES: 6.6 %-pts); 2 or more home visits in last trimester (%) (DiD ES: 10.7 %-pts). *Home visits after delivery.* At least one home visit within 24 h of delivery, among women with home delivery (%) (F/U C: 35%; F/U I: 34%; ES: -1.0 %-pts); Any visit in first week (%) (F/U C: 60%; F/U I: 72%; ES: 12.0 %-pts); Any visit after first week but before first month (ie, weeks 2-4) (%) (F/U C: 45%; F/U I: 48%; ES: 3.0 %-pts). *Home visits on feeding.* Any home visit on complementary feeding (%) (DiD ES: 8.3 %-pts). *Home visits on FP or postpartum health.* Any home visit about family planning or postpartum health (%) (DiD ES: 6.6 %-pts).	**HCP practice outcome MES:** 6.6 %-pts (primary outcome)<br><br>**Supervision outcome MES:** 2.8 %-pts (secondary outcomes)	**Interpretation of results.** Study wording does not suggest that this was an equivalency trial (wording indicates that it was a superiority trial).<br><br>The intervention was for CHWs, but the study intentionally examined the effect on supervision outcomes, which were not primary outcomes.<br><br>**From Table 3.** The intervention-vs-control results are shown separately for the two CHW types (ASHAs & AWWs). I.e., two effect sizes [ES] per indicator. I = intervention, C = control. Among these 7 eligible outcomes (i.e., with control values < 95%) and thus 14 effect sizes, the **MES = 2.8 %-pts**.

Country	Reference	Methods and risk of bias (ROB)	Results of ICT vs. comparison group (BL = baseline, C = control arm, ES = effect size, F/U = follow-up, I = intervention arm)	Median effect size (ICT vs. comparison)	Comments
			• Note 1. RMNCHN outcomes from Table 4 were excluded when they depended on mothers' (not HCPs') behaviors, such as % of women who delivered at a facility.<br>• Note 2. Most effect sizes are model-based "difference-in-differences" estimates, which are labeled as "DiD ES".<br>• Note 3. Outcomes on home visits after delivery were not measured at baseline. Thus, ES = F/U I – F/U C.<br><br>There were also 14 effect sizes from secondary outcomes related to supervision (see comments for details).		• Met with supervisor in past 3 months outside sub-center meeting (%). AWW C: 99%; AWW I: 99%; AWW ES: 0%-pts; ASHA C: 97%; ASHA I: 97%; ASHA ES: 0%-pts. *[These effect sizes are ignored because the baseline or control value is ≥95% (i.e., no performance problem existed, very little room to improve, and so not a good context to evaluate any intervention)]*<br>• Number of times met with supervisor in past 3 months outside sub-center meeting (mean). AWW C: 3.5; AWW I: 3.6; **AWW ES: 2.9%** (i.e., 100 x {[3.6 – 3.5]/3.5}); ASHA C: 3.9; ASHA I: 4.0; **ASHA ES: 2.6%** (i.e., 100 x {[4.0 – 3.9]/3.9}).<br>• Supervisor always available by phone or in person when FLW needs to reach her (vs sometimes or never) (%). AWW C: 77%; AWW I: 79%; **AWW ES: 2 %-pts**; ASHA C: 80%; ASHA I: 83%; **ASHA ES: 3 %-pts**.<br>• During recent visits, supervisor, most of the time:<br>▪ Brought outstanding (due) visits to the FLW's attention (%). AWW C: 78%; AWW I: 73%; **AWW ES: -5 %-pts**; ASHA C: 75%; ASHA I: 73%; **ASHA ES: -2 %-pts**.<br>▪ Gave the FLW guidance on what information to give to households (%). AWW C: 52%; AWW I: 55%; **AWW ES: 3 %-pts**; ASHA C: 56%; ASHA I: 58%; **ASHA ES: 2 %-pts**.<br>▪ Gave the FLW guidance on how to communicate effectively with households (%). AWW C: 41%; AWW I: 47%; **AWW ES: 6 %-pts**; ASHA C: 38%; ASHA I: 48%; **ASHA ES: 10 %-pts**.<br>▪ Talked to the households the FLW was finding difficult to convince (%). AWW C: 35%; AWW I: 43%; **AWW ES: 8 %-pts**; ASHA C: 43%; ASHA I: 43%; **ASHA ES: 0 %-pts**.<br>▪ Helped FLW coordinate with her counterpart (%). AWW C: 50%; AWW I: 55%; **AWW ES: 5 %-pts**; ASHA C: 53%; ASHA I: 54%; **ASHA ES: 1 %-pts**.

Country	Reference	Methods and risk of bias (ROB)	Results of ICT vs. comparison group (BL = baseline, C = control arm, ES = effect size, F/U = follow-up, I = intervention arm)	Median effect size (ICT vs. comparison)	Comments
India	Gautham et al. 2015	• RCT of intervention (mobile phone with an mHealth application installed) vs. comparison (paper guidelines + mobile phone without the mHealth application installed). Both groups received a 2-day training on IMCI and Integrated Management of Adult Illnesses (IMAI) guidelines and use of the mHealth system.<br>• Setting: CHWs providing IMCI and IMAI care<br>• Intervention: mobile phone with an mHealth application (guideVue® system) with educational materials to present information with audio (speech), images, and videos.<br>• ROB: Moderate (because of contamination—see comment)	**HCP practice outcomes from Figures 7 and 8.** The primary outcome was adherence to the case-management guideline, in terms of a percentage. See comment for details on the figures. Mean outcome value for male CHWs in the intervention group = 71.12%, i.e., (65.49% for patient 0 + 76.75% for patient 7)/2. Mean outcome value for male CHWs in the control group = 53.60%, i.e., (38.69% for patient 0 + 68.50% for patient 7)/2. Mean outcome value for female CHWs in the intervention group = 69.00%, i.e., (63.37% for patient 0 + 74.63% for patient 7)/2. Mean outcome value for female CHWs in the control group = 66.34%, i.e., (51.42% for patient 0 + 81.25% for patient 7)/2. Mean outcome value for all CHWs in the intervention group = 70.06%, i.e., (71.12% for male CHWs + 69.00% for female CHWs)/2. Mean outcome value for all CHWs in the control group = 59.97%, i.e., (53.60% for male CHWs + 66.34% for female CHWs)/2.<br>ES: 10.1 %-pts, i.e., 70.06% - 59.97%, rounded to the tenths place.	**HCP practice outcome MES:** 10.1 %-pts	**Interpretation of results.** Study wording does not suggest that this was an equivalency trial.<br><br>**Contamination concern.** Page 263 states that in one of the two evaluation sites, CHWs in the control group had "opportunities to intermingle with and observe the ways of the experimental group…. As it was a real-time field setting, both groups were at work in the hospital's outreach clinic, and it was difficult to isolate one group from the other."<br><br>**Results on intervention effectiveness.** Figure 7 shows model-based estimates of the outcome for male CHWs (separately for the intervention and control groups) as a function of the order of the eight patients that each CHW treated during the endline evaluation. For example, among control CHWs, the outcome had an estimated value of 38.69% for the first patient seen, 42.95% for the second patient seen, and so on. Figure 8 had the same information for female CHWs. In all cases, the outcome values by patient order were estimated by a straight line. Thus, to estimate the outcome for a study group (e.g., male control CHWS), the average value of the predicted line was calculated—that is: (outcome value for patient 0 + value for patient 7)/2. Then, as there were equal numbers of male and female CHWs, the male and female averages for each study group were averaged—that is: (mean outcome value for male CHWs + mean value for female CHWs)/2.
India	Modi et al. 2019; Modi et al. 2017	• RCT (intervention arm vs. true control arm)<br>• Setting: CHWs (i.e., Accredited Social Health Activists, or ASHAs) that provide MNCH services<br>• Intervention: Mobile phone and web-based application (see comment)<br>• ROB: Low	**HCP practice outcomes.**<br>**The primary outcomes (from Table 4) were:**<br>• Composite coverage index (see comment). F/U I: 43.0%; F/U C: 38.5%; unadjusted ES: 4.6 %-pts; **adjusted ES: 4.9 %-pts**<br>• At least two home visits by ASHA in first week after delivery. F/U I: 32.4%; F/U C: 22.9%; unadjusted ES: 9.5 %-pts; **adjusted ES: 10.2 %-pts**<br><br>Note that effect sizes were from models and labeled as "adjusted effect size".<br><br>No quantitative information on delivery of supervision	**HCP practice outcome MES:** 7.6 %-pts	**Interpretation of results.** Study wording does not suggest that this was an equivalency trial.<br><br>**Intervention details.** ImTeCHO was a mobile-phone–and web-based application that included "various technology-based job aids to facilitate scheduling of home visits, screening for complications, counseling during home visits, and supportive supervision by PHC staff."<br><br>**Composite coverage index** (a primary outcome) included indicators of: ASHA home visits, antenatal care, facility delivery, postnatal home visits, and vaccinations and exclusive breastfeeding for young infants.

Country	Reference	Methods and risk of bias (ROB)	Results of ICT vs. comparison group (BL = baseline, C = control arm, ES = effect size, F/U = follow-up, I = intervention arm)	Median effect size (ICT vs. comparison)	Comments
Kenya	Toda et al. 2016; Toda et al. 2017	• RCT of intervention (mSOS system plus a 1-day training on using mSOS plus paper-based disease reporting) vs. comparison (paper-based disease reporting). Both groups received a 1-day refresher training on Integrated Disease Surveillance and Response (IDSR) before randomization.<br>• Setting: medical officers and other HCPs at health facilities<br>• Intervention: mSOS (a mobile short-message-service [SMS] disease outbreak alert system for reporting immediately notifiable diseases, such as measles)<br>• ROB: Low	**HCP practice outcome**<br>**The primary outcome was the % of disease cases that required immediate notification that were reported within the time specified.**<br>From Table 2. F/U I: 19.23% (i.e., 25/130); F/U C: 2.56% (i.e., 1/39).<br>From page 712. BL I: 0/19 (0%); BL C: 1/17 (5.88%).<br>ES = (19.23% - 0%) - (2.56% - 5.88%) = 22.55 %-pts, or 22.6 %-pts (rounded to tenths place).	**HCP practice outcome MES:**<br>22.6 %-pts	**Interpretation of results.** Study wording does not suggest that this was an equivalency trial.<br><br>**Secondary outcome.**<br>Determining, from among the cases for which notifications were sent, the proportion for which response actions were taken. There was one measles outbreak detected (in the intervention group), and subcounty disease surveillance coordinators responded. No effect size calculated.
Malawi	Chirambo et al. 2021; CHW Central 2020	• RCT of intervention (CHWs using mobile smartphones with a clinical decision support application, or e-CCM plus use of standard CCM paper forms) vs. comparison (CHWs using standard CCM paper forms).<br>• Setting: CHWs doing CCM<br>• Intervention: Mobile smartphone with a clinical decision support tool application (called "SL e-CCM app"). CHWs received a total of 3 days of training on study procedures and how to use the smartphone and SL e-CCM app.<br>• ROB: Low	**HCP practice outcomes from Table 2**<br><br>Primary outcomes that was an HCP practice outcome was urgent referrals to higher-level health facilities. F/U C: 5.23% (i.e., 179/3421); F/U I: 5.58% (i.e., 198/3544); ES: 6.7% (i.e., 100% x [5.58% – 5.23%]/5.23%). An increase in urgent referrals was interpreted as improved HCP performance.	**HCP practice outcome MES:**<br>6.7 %-pts	**Interpretation of results.** Study wording does not suggest that this was an equivalency trial.<br><br>Malawi's Health Surveillance Assistants (HSAs) were considered as CHWs (CHW Central 2020).<br><br>Other primary outcomes. children in the intervention arm had lower odds of attending a repeat HSA consultation (OR 0.45) or being admitted to a hospital (OR 0.75).
Mali	Whidden et al. 2018 [ICT supervision]	• RCT of intervention (supervisors had a CHW Supervisor Dashboard) vs. comparison (supervisors did not have the Dashboard)<br>• Setting: CHWs doing proactive community case management (CCM), which involves making household visits<br>• Intervention: CHW Supervisor Dashboard (an ICT intervention that allowed supervisors to monitor CHW performance on household visits and quality of care)<br>• ROB: Moderate (because of contamination—see comment)	**HCP practice outcomes from Table 2.**<br>Primary outcome. Number of households that CHWs visited per month (BL C: 291.8; F/U C: 501.4; % improvement over time C: 71.8% [i.e., 100 x {(501.4 – 291.8)/291.8}]; BL I: 294.6; F/U I: 544.1; % improvement over time I: 84.7% [i.e., 100 x {(544.1 – 294.6)/ 294.6}]; **ES: 12.9 %-pts** [i.e., 84.7 – 71.8]).	**HCP practice outcome MES:**<br>12.9 %-pts<br><br>**Supervision outcome MES:**<br>12.9 %-pts (same as HCP practice outcome because intervention was for supervisors and objective was to improve HCP performance via strengthened supervision)	**Interpretation of results.** Study wording does not suggest that this was an equivalency trial.<br><br>**Contamination concern.** In addition to monthly individual supervision visits to CHWs, there were weekly group sessions with CHWs in both intervention and control arms, during which the supervisor led discussion of the common challenges and potential solutions faced by CHWs, which represented contamination [see page 10].<br><br>**Secondary outcomes.**<br>• % of children under five treated within 24 hours of symptom onset (BL I: 71.0%; BL C: 71.1%; F/U I: 86.6%; F/U C: 82.7%; **ES: 4.0 %-pts** [i.e., (86.6 – 71.0) – (82.7 – 71.1)]).<br>• % of children under five treated without protocol error (BL I: 52.3%; BL C: 49.1%; F/U I: 71.0%; F/U C: 63.4%; **ES: 4.4 %-pts** [i.e., (71.0 – 52.3) – (63.4 – 49.1)]). Note that a national stock-out of zinc prevented some children from being treated "without protocol error" (i.e., not CHWs' fault).

Country	Reference	Methods and risk of bias (ROB)	Results of ICT vs. comparison group (BL = baseline, C = control arm, ES = effect size, F/U = follow-up, I = intervention arm)	Median effect size (ICT vs. comparison)	Comments
Mali	Yang et al. 2021	• RCT of intervention (CHWs using mobile phones with a new application) vs. comparison (CHWs using mobile phones with an older application). See comment.<br>• Setting: CHWs doing proactive CCM, which involves making household visits<br>• Intervention: New mobile phone application (see comment).<br>• ROB: Moderate (because of contamination concern)	**HCP practice outcomes from Table 3.**<br>Primary outcome. % of households visited at least twice monthly (BL I: 43.1%; BL C: 39.5%; F/U I: 70.9%; F/U C: 51.8%; unadjusted ES: 15.5 %-pts [i.e., (70.9 – 43.1) – (51.8 – 39.5)]; **adjusted ES [from page 8]: 13.6 %-pts**).<br><br>Note that effect sizes were from models and labeled as adjusted "DID % point change".	**HCP practice outcome MES:**<br>13.6 %-pts	**Interpretation of results.** Study wording does not suggest that this was an equivalency trial.<br><br>**Comment on study design.** The intervention was a new "UHC Mode" application that helped CHWs plan home visits. Although both study arms had an ICT intervention, the performance of CHWs in the control arm was clearly considered inadequate, so it is reasonable to consider that this trial was testing the effect of ICT (i.e., the new UHC Mode application).<br><br>**Contamination concern.** Improvement in both arms might be partly due to the CHW Supervisor Dashboard (see Whidden 2018), which was introduced in both arms in the middle of the baseline period.
Mozambique	Soremekun et al. 2023	• RCT of intervention (ICT) vs. comparison (no new intervention). Both arms received basic training, commodities, and monthly supervision.<br>• Setting: CHWs doing iCCM<br>• Intervention: mobile smartphone with the "inSCALE Mobile VHT system" (see comment for details)<br>• ROB: Low	**HCP practice outcomes from Table 7.**<br>Among children seen by CHWs, % of suspected malaria, diarrhea, or suspected pneumonia that were treated appropriately. F/U C: 49.5% (i.e., 146/295); F/U I: 61.9% (i.e., 148/239); ES: 12.4 %-pts	**HCP practice outcome MES:**<br>12.4 %-pts	**Interpretation of results.** Study wording does not suggest that this was an equivalency trial (wording indicates that it was a superiority trial).<br><br>**Intervention details.** The "inSCALE Mobile VHT system" can send weekly reports on patients seen (sex, symptoms, classifications, treatments) and current drug stock levels, and can receive motivational feedback messages. Also, it has electronic clinical decision algorithms and allows for free calls between CHWs and supervisors.<br><br>inSCALE = interventions at scale for community access and lasting effects.
Niger	Zakus et al. 2019 [ICT supervision]	• RCT of intervention (iCCM with smart phone) vs. comparison (iCCM with paper tools). Both arms received standard 10-day iCCM training.<br>• Setting: CHWs doing iCCM<br>• Intervention: A smartphone with an application that helped CHWs follow diagnosis and treatment protocols during iCCM encounters. It also contained a module for the control of drugs and supplies. All intervention CHWs were trained for five days on the use of the smartphone and application.<br>• ROB: Low	**HCP practice outcomes from Table 3.**<br>Primary outcome was a quality-of-care score composed of diagnostic and treatment variables (maximum quality-of-care score was 31 points; see Table 2 for details). F/U C: 25.3/31 (i.e., 81.6%); F/U I: 26.2 (i.e., 84.5%); ES: 2.9 %-pts (i.e., 84.5% - 81.5%).<br><br>There were also nine secondary outcomes related to supervision (see comments for details).	**HCP practice outcome MES:**<br>2.9 %-pts (primary outcome)<br><br>**Supervision outcome MES:**<br>–4.8 %-pts (secondary outcomes)	**Interpretation of results.** Study wording does not suggest that this was an equivalency trial (wording indicates that it was a superiority trial).<br><br>The intervention was for CHWs, but the study intentionally examined the effect on supervision outcomes, which were not primary outcomes.<br>• **From Figure 5 (supervisory actions performed).** Check records (I: 93.3%; C: 98.1%; **ES: -4.8 %-pts**); delivered supplies (I: 88.9%; C: 90.7%; **ES: -1.8 %-pts**); observed CHW managing a child (I: 13.3%; C: 13.0%; **ES: 0.3 %-pts**); positive feedback (I: 71.1%; C: 61.1%; **ES: 10.0 %-pts**); corrected or reminded (I: 66.7%; C: 74.1%; **ES: -7.4 %-pts**); discussed problems (I: 53.3%; C: 74.1%; **ES: -20.8 %-pts**); answered work questions (I: 57.8%; C: 68.5%; **ES: -10.7 %-pts**); used supervision checklist (I: 51.1%; C: 50.0%; **ES: 1.1 %-pts**).

Country	Reference	Methods and risk of bias (ROB)	Results of ICT vs. comparison group (BL = baseline, C = control arm, ES = effect size, F/U = follow-up, I = intervention arm)	Median effect size (ICT vs. comparison)	Comments
					• **From Table 1.** % of CHWs that received zero supervisory visits in the past 3 months (a harmful outcome, and thus the effect size [intervention % minus control %] is multiplied by negative 1). I: 31.8%; C: 15.6%; **ES: -16.2 %-pts**.<br>• **MES of 9 supervision effect sizes = –4.8 %-pts.**
Pakistan	Rahman et al. 2019; Zafar et al. 2016 [ICT supervision, although intervention involved ICT for training and supervision]	• Equivalency (non-inferiority) RCT of an ICT intervention arm (e-learning and distance supervision of supervisors) vs. a comparison arm (in-person training and in-person supervision of supervisors). CHWs in both arms delivered mental health care to women in the community using the original paper-based manual.<br>• Setting: CHWs providing mental health care<br>• Intervention: A tablet-based application (see comments)<br>• ROB: moderate (because of 25% loss to follow-up in both arms)	**HCP practice outcomes from Table 3.**<br>Primary outcome was a quality-of-care score (maximum score = 54) related to mental health therapy, which was measured 3 months post-training. F/U C: 82.4% (i.e., 44.48/54); F/U I: 80.8% (i.e., 43.63/54); ES: -1.6 %-pts.	**HCP practice outcome MES:**<br>–1.6 %-pts (Equivalency study, so effect size close to zero is a desirable result)	**Interpretation of results.** Study was clearly an equivalency or non-inferiority trial.<br><br>**Intervention details.** The training intervention involved: a) a tablet-based application allowing standard training (with videos and interactive features) to be delivered by non-specialist trainers; and b) a cascade training/supervision model where a master trainer trained non-specialist trainers, who in turn trained CHW supervisors, who in turn cascaded the training to CHWs. The supervision intervention involved distance supervision of non-specialist trainers by a master trainer via monthly Skype calls, distance group supervision of CHW supervisors by non-specialist trainers via monthly Skype calls, and in-person supervision of CHWs by CHW supervisors.<br><br>**Secondary outcome**. Training and supervision cost per CHW was USD 170 in the comparison arm and USD 117 in the intervention arm. Thus, the intervention was 31.2% (i.e., [117 – 170]/170) less expensive than the comparison arm.
Palestine	Venkateswaran et al. 2022	• RCT (intervention arm vs. true control arm)<br>• RCT of intervention (electronic patient registry plus eCDSS) vs. comparison (paper records).<br>• Setting: primary health-care clinics offering routine antenatal care<br>• Intervention: electronic patient registry plus eCDSS<br>• ROB: Low	**HCP practice outcomes from Table 3.** Screening and management during eligible contacts of:<br>• Anemia. F/U C: 28.9%; F/U I: 44.3%; ES: 15.4 %-pts.<br>• Gestational diabetes. F/U C: 39.7%; F/U I: 50.7%; ES: 11.0 %-pts.<br>• Hypertension. F/U C: 94.7%; F/U I: 96.6%; ES: 1.9 %-pts.<br>• Abnormal fetal growth. F/U C: 78.8%; F/U I: 68.9%; ES: –9.9 %-pts.	**HCP practice outcome MES:**<br>6.5 %-pts	**Interpretation of results.** Study wording does not suggest that this was an equivalency trial (wording indicates that it was a superiority trial).<br><br>Primary health outcome was a composite of moderate or severe anemia; severe hypertension; large-for-gestational-age baby; malpresentation and small-for-gestational-age baby undetected before delivery. F/U C: 21.9%; F/U I: 21.7%; ES (based on adjusted odds ratio of 0.99): 1%.
South Africa	Horwood et al. 2024	• RCT of intervention (ICT clinical support system) vs. comparison (standard IMCI with paper forms). Both study arms received a 3-day IMCI refresher training and two mentoring visits by an IMCI trainer.<br>• Setting: Primary health facilities caring for sick children with IMCI<br>• Intervention: Electronic Clinical Decision Support System (eCDSS) (on computers) to help HCPs adhere to IMCI guidelines plus a 1-day training on basic computer skills plus technical support for the computers.	**HCP practice outcome from Table 5.**<br>The primary outcome was the % of sick children who received all medications indicated. I F/U: 81.6%; C F/U: 90.6%; ES: -9.0 %-pts.	**HCP practice outcome MES:**<br>–9.0 %-pts	**Interpretation of results.** Study wording does not suggest that this was an equivalency trial (wording indicates that it was a superiority trial).<br><br>**Secondary outcomes (HCP practice outcomes)**<br>• From Table 2. Duration of the consultations (median time. C F/U: 25 minutes; I F/U: 28 minutes; the relative difference = 100% x ([28 – 25]/25) = 12.0%, but it is unclear whether the authors considered longer consultations or shorter consultations as preferable.

Country	Reference	Methods and risk of bias (ROB)	Results of ICT vs. comparison group (BL = baseline, C = control arm, ES = effect size, F/U = follow-up, I = intervention arm)	Median effect size (ICT vs. comparison)	Comments
		• ROB: Low			• **From Table 3.** Children with all presenting symptoms correctly classified. I F/U: 54.1%; C F/U: 58.1%; **ES: -4.0 %-pts**<br>**Additional HCP practice outcome**<br>• **From Table 5.** Any over-prescription (includes any medication not indicated by IMCI). I F/U: 31.6%; C F/U: 14.4%; difference = 17.2 %-pts, however, as a decrease is an improvement, the effect size is multiplied by -1, i.e., **ES: -17.2 %-pts.**
Tanzania	Perri-Moore et al. 2015	• RCT of intervention (ICT on IMCI counseling) vs. comparison (IMCI counseling with paper forms). Both groups had an initial 2-day large-group workshop training (1.5-days on IMCI protocol plus a 0.5-day on counseling, with separate training for intervention arm, which focused on use of ICT, and control arm, which focused on counseling with paper forms). Both groups also received small group training on counseling (separately for each arm to practice using the ICT and paper platforms).<br>• Setting: Health facilities caring for sick children with IMCI<br>• Intervention: "eIMCI", which was a mobile phone with video and other functions (see comment)<br>• ROB: Low	**HCP practice outcomes from page 44.**<br>Primary outcomes were on communication between HCPs and children's caretakers, in terms of what HCPs said to caretakers during the visit—i.e., HCP verbalized: the problem (F/U I: 98.8%; F/U C: 77.8%; ES: 21.0 %-pts); number of days to return to the facility (F/U I: 66.3%; F/U C: 45.0%; ES: 21.3 %-pts); at least one sign of worsening condition (i.e., return signs) (F/U I: 96.5%; F/U C: 92.2%; ES: 4.3 %-pts); medication type or name (F/U I: 97.0%; F/U C: 80.8%; ES: 16.2 %-pts); medication frequency (F/U I: 93.2%; F/U C: 79.2%; ES: 14.0 %-pts); medication duration (F/U I: 92.6%; F/U C: 86.9%; ES: 5.7 %-pts).	**HCP practice outcome MES:**<br>15.1 %-pts	**Interpretation of results.** Study wording does not suggest that this was an equivalency trial (wording indicates that it was a superiority trial).<br><br>**Intervention details.** Mobile phones had a short video to educate caretakers on health information, with prompts containing key messages for caretakers and a customized summary screen of the results of the consultation. Control arm (paper arm) provided equivalent information as in the intervention arm, but as written text.<br><br>**Patient knowledge outcomes (page 44).** There were also primary outcomes on communication between HCPs and children's caretakers in terms of what caretakers recalled from what HCPs told them—i.e., the caretaker recalled: the problem (F/U I: 84.1%; F/U C: 68.6%; **ES: 15.5 %-pts**); number of days to return to the facility (F/U I: 32.5%; F/U C: 16.0%; **ES: 16.5 %-pts**); at least one sign of worsening condition (i.e., return signs) (F/U I: 77.1%; F/U C: 50.0%; **ES: 27.1 %-pts**); medication type or name (F/U I: 78.5%; F/U C: 57.8%; **ES: 20.7 %-pts**); medication frequency (F/U I: 63.8%; F/U C: 41.2%; **ES: 22.6 %-pts**); medication duration (F/U I: 28.0%; F/U C: 20.8%; **ES: 7.2 %-pts**). **MES: 18.6 %-pts.**
Uganda	Källander et al. 2023	• RCT of intervention (ICT) vs. comparison (no new intervention). Both arms received basic training, commodities, and monthly supervision.<br>• Setting: CHWs doing iCCM<br>• Intervention: mobile smartphone with the "inSCALE Mobile VHT system" (see comment for details)<br>• ROB: Low	**HCP practice outcomes from Table 7.**<br>Among children seen by CHWs, % of suspected malaria, diarrhea, or suspected pneumonia that were treated appropriately. F/U C: 73.6% (i.e., 352/478); F/U I: 78.5% (i.e., 357/455); ES: 4.9 %-pts	**HCP practice outcome MES:**<br>4.9 %-pts	**Interpretation of results.** Study wording does not suggest that this was an equivalency trial (wording indicates that it was a superiority trial).<br><br>**Intervention details.** The "inSCALE Mobile VHT system" can send weekly reports on patients seen (sex, symptoms, classifications, treatments) and current drug stock levels, and can receive motivational feedback messages. Also, it has electronic clinical decision algorithms and allows for free calls between CHWs and supervisors.<br><br>inSCALE = interventions at scale for community access and lasting effects.

Country	Reference	Methods and risk of bias (ROB)	Results of ICT vs. comparison group (BL = baseline, C = control arm, ES = effect size, F/U = follow-up, I = intervention arm)	Median effect size (ICT vs. comparison)	Comments
Zambia	Biemba et al. 2017; Biemba et al. 2020 [ICT supervision]	• RCT of intervention (ICT) vs. comparison (usual paper reporting forms)<br>• Setting: CHWs doing iCCM<br>• Intervention: mHealth-enhanced iCCM supportive supervision and supply chain management (see comment for details)<br>• ROB: Low<br>• Limitation: Implementation period was 5.5 months (due to funding constraints of the study), which the authors said might have been insufficient to show the full effect of the intervention.	**HCP practice outcomes from Table 2.**<br>Primary outcome was the % of children aged 2-59 months receiving appropriate treatment for malaria, pneumonia, or diarrhea from an iCCM trained CHW. F/U C: 63.3%; F/U I: 65.9%; ES: 2.6 %-pts (i.e., 65.9% - 63.3%).<br><br>There were also two secondary outcomes related to supervision (see comments for details).	**HCP practice outcome MES:** 2.6 %-pts (primary outcome)<br><br>**Supervision outcome MES:** 4.5 %-pts (secondary outcomes)	**Interpretation of results.** Study wording does not suggest that this was an equivalency trial (wording indicates that it was a superiority trial).<br><br>**Intervention details.** CHWs used community DHIS2 platform on mobile phones to report weekly iCCM caseloads and request commodities. Their supervisors received electronic reports on caseloads and monthly automated supervision reminders. When supervisors received requests for commodities, they organized the supplies and notified CHWs for collection. Supervisors used the applications to provide feedback to CHWs on referral case outcomes and notify CHWs to collect drugs and diagnostic supplies. Monthly automated messages were sent to supervisors reminding them to conduct supervisory visits with CHWs.<br><br>The intervention was for CHWs, but the study intentionally examined the effect on supervision outcomes, which were not primary outcomes.<br>• **From Table 3.** In the intervention clusters, 61.3% of monthly supervision visits took place vs 52.0% in the controls (**ES: 9.3 %-pts**).<br>• **From Table 4.** iCCM commodities available on day of visit (all of these: AL, mRDTs, amoxicillin, ORS, zinc) was 12.9% in intervention arm and 13.3% in controls (**ES: -0.4 %-pts**).<br>• **MES of 2 supervision effect sizes = 4.5 %-pts.**

Table S6. The effect of information and communication technology (ICT) (i.e., digital interventions) for healthcare providers (HCPs) and their supervisors on HCP practice outcomes in low- and middle-income countries. Older studies from the Health Care Provider Performance Review (HCPPR).

Country	Reference	HCPPR identification number*	Median effect size (ICT vs. comparison)	Risk of bias
Studies of professional HCPs (e.g., physicians, nurses, and midwives)				
Cameroon	Ateudjieu J, Stoll B, Nguefack-Tsague G, Tchangou C, Genton B. Vaccines safety; effect of supervision or SMS on reporting rates of adverse events following immunization (AEFI) with meningitis vaccine (MenAfriVac): a randomized controlled trial. *Vaccine*. 2014;32(43): 5662–5668.	29070000112	–38.9 %-pts	Moderate
Kenya	Zurovac D, Sudoi RK, Akhwale WS, Ndiritu M, Hamer DH, Rowe AK, et al. The effect of mobile phone text-message reminders on Kenyan health workers' adherence to malaria treatment guidelines: a cluster randomised trial. *Lancet*. 2011;378(9793):795–803.	27210000112	24.5 %-pts	Low
Malawi	Steinhardt LC, Mathanga D, Mwandama D, Nsona H, Moyo D, Gumbo A, et al. The effect of text message reminders to health workers on quality of care for malaria, pneumonia, and diarrhea in Malawi: a randomized controlled trial. Paper presented at 65th Annual meeting of American Society of Tropical Medicine and Hygiene; November 13–17, 2016; Atlanta, GA.<br><br>Steinhardt LC, Mathanga D, Mwandama D, Nsona H, Moyo D, Gumbo A, et al. The effect of text message reminders to health workers on quality of care for malaria, pneumonia, and diarrhea in Malawi: a randomized controlled trial. *Am J Trop Med Hyg*. 2019;100(2):460–469.	30020000112	–4.7 %-pts	Moderate
Malawi	Steinhardt LC, Mathanga D, Mwandama D, Nsona H, Moyo D, Gumbo A, et al. The effect of text message reminders to health workers on quality of care for malaria, pneumonia, and diarrhea in Malawi: a randomized controlled trial. Paper presented at 65th Annual meeting of American Society of Tropical Medicine and Hygiene; November 13–17, 2016; Atlanta, GA.<br><br>Steinhardt LC, Mathanga D, Mwandama D, Nsona H, Moyo D, Gumbo A, et al. The effect of text message reminders to health workers on quality of care for malaria, pneumonia, and diarrhea in Malawi: a randomized controlled trial. *Am J Trop Med Hyg*. 2019;100(2):460–469.	30020000113	3.9 %-pts	Moderate
Tanzania	Bruxvoort K, Festo C, Kalolella A, Cairns M, Lyaruu P, Kenani M, et al. Cluster randomized trial of text message reminders to retail staff in Tanzanian drug shops dispensing artemether-lumefantrine: effect on dispenser knowledge and patient adherence. *Am J Trop Med Hyg*. 2014;91(4):844–853.	26150000112	–0.1 %-pts	Moderate
Studies of lay HCPs (e.g., community health workers)				
Malawi	Joos O, Silva R, Amouzou A, Moulton LH, Perin J, Bryce J, et al. Evaluation of a mHealth data quality intervention to improve documentation of pregnancy outcomes by health surveillance assistants in Malawi: a cluster randomized trial. *PLoS One*. 2016;11(1):e0145238.	31119010222	–7.8 %-pts	Moderate
Malawi	Joos O, Silva R, Amouzou A, Moulton LH, Perin J, Bryce J, et al. Evaluation of a mHealth data quality intervention to improve documentation of pregnancy outcomes by health surveillance assistants in Malawi: a cluster randomized trial. *PLoS One*. 2016;11(1):e0145238.	31119010211	–4.4 %-pts	Moderate
Tanzania	DeRenzi B, Findlater L, Payne J, Birnbaum B, Mangilima J, Parikh T, et al. Improving community health worker performance through automated SMS. Paper presented at: ACM 2012. Proceedings of the Fifth International Conference on Information and Communication Technologies and Development: March 12–15, 2012; Atlanta, GA.	29130000112	68.4 %-pts	Moderate

* This is the “COMP_IDnew” variable in the HCPPR dataset.

REFERENCES

Agarwal S, Rosenblum L, Goldschmidt T, Carras M, Goel N, Labrique AB. Mobile Technology in Support of Frontline Health Workers. Johns Hopkins University Global mHealth Initiative, 2016. Accessed October 30, 2020.
http://media.wix.com/ugd/f85b85_cc8c132e31014d91b108f8dba524fb86.pdf

Ahuja N, Källander K. Leveraging Digital Channels to Build Health Worker Capacity at the Last Mile. *Journal of Health Informatics in Africa*. 2023;10(1):19–30.

Aryee GFB, Amoadu M, Obeng P, Sarkwah HN, Malcalm E, Abraham SA, et al. Effectiveness of eLearning programme for capacity building of healthcare professionals: a systematic review. *Hum Resour Health*. 2024;22(1):60.

Bernasconi A, Landi M, Yah CS, van der Sande MAB. Information and communication technology to enhance the implementation of the Integrated Management of Childhood Illness: a systematic review and meta-analysis. *Mayo Clinic Proceedings: Digital Health*. 2024;2(3):438–452.

Biemba G, Chiluba B, Yeboah-Antwi K, Silavwe V, Lunze K, Mwale RK, et al. A mobile-based community health management information system for community health workers and their supervisors in 2 districts of Zambia. *Glob Health Sci Pract*. 2017;5(3):486–494.

Biemba G, Chiluba B, Yeboah-Antwi K, Silavwe V, Lunze K, Mwale RK, Hamer DH, MacLeod WB. Impact of mobile health-enhanced supportive supervision and supply chain management on appropriate integrated community case management of malaria, diarrhoea, and pneumonia in children 2-59 months: A cluster randomised trial in Eastern Province, Zambia. *J Glob Health*. 2020;10(1):010425.

Carmichael SL, Mehta K, Srikantiah S, et al. Use of mobile technology by frontline health workers to promote reproductive, maternal, newborn and child health and nutrition: a cluster randomized controlled trial in Bihar, India. *J Glob Health*. 2019;9(2):0204249.

Chen Y, Yang K, Jing T, Tian J, Shen X, Xie C, Ma B, Liu Y, Yao L, Cao X. Use of text messages to communicate clinical recommendations to health workers in rural China: a cluster-randomized trial. *Bull World Health Organ*. 2014;92(7):474–81.

Chirambo GB, Thompson M, Hardy V, Ide N, Hwang PH, Dharmayat K, et al. Effectiveness of smartphone-based community case management on the urgent referral, reconsultation, and hospitalization of children aged under 5 years in Malawi: cluster-randomized, stepped-wedge trial. *J Med Internet Res*. 2021;23(10):e25777.

CHW Central. Malawi's Community Health Worker Program. September 24, 2020. Accessed February 1, 2025. https://chwcentral.org/malawis-community-health-worker-program/

Cockburn N, Osborne C, Withana S, Elsmore A, Nanjappa R, South M, et al. Clinical decision support systems for maternity care: a systematic review and meta-analysis. *EClinicalMedicine*. 2024;76:102822.

Deussom R, Mwarey D, Bayu M, Abdullah SS, Marcus R. Systematic review of performance-enhancing health worker supervision approaches in low- and middle-income countries. *Hum Resour Health*. 2022;20:2.

Gautham M, Iyengar MS, Johnson CW. Mobile phone-based clinical guidance for rural health providers in India. *Health Informatics J*. 2015;21(4):253–66. Personal communication from Sriram Iyengar, University of Arizona College of Medicine, on March 3, 2025.

Horwood C, Haskins L, Mapumulo S, Connolly C, Luthuli S, Jensen C, et al. Electronic Integrated Management of Childhood Illness (eIMCI): a randomized controlled trial to evaluate an electronic clinical decision-making support system for management of sick children in primary health care facilities in South Africa. *BMC Health Serv Res*. 2024;24:177.

Källander K, Soremekun S, Strachan DL, et al. Improving community health worker treatment for malaria, diarrhoea, and pneumonia in Uganda through inSCALE community and mHealth innovations: A cluster randomised controlled trial. *PLoS Digit Health*. 2023;2(6):e0000217. Personal communication from Seyi Soremekun, London School of Hygiene and Tropical Medicine, on February 4, 2025.

Keitel K, D'Acremont V. Electronic clinical decision algorithms for the integrated primary care management of febrile children in low-resource settings: review of existing tools. *Clin Microbiol Infect*. 2018;24(8):845–55.

Kruk ME, Gage AD, Arsenault C, et al. High-quality health systems in the Sustainable Development Goals era: time for a revolution. *Lancet Global Health* 2018 Nov;6(11):e1196–e1252.

Modi D, Desai S, Dave K, Shah S, Desai G, Dholakia N, et al. Cluster randomized trial of a mHealth intervention "ImTeCHO" to improve delivery of proven maternal, neonatal, and child care interventions through community-based Accredited Social Health Activists (ASHAs) by enhancing their motivation and strengthening. *Trials*. 2017;18:270.

Modi D, Dholakia N, Gopalan R, Venkatraman S, Dave K, Shah S, et al. mHealth intervention "ImTeCHO" to improve delivery of maternal, neonatal, and child care services—A cluster-randomized trial in tribal areas of Gujarat, India. *PLoS Med*. 2019;16(10):e1002939.

Perri-Moore S, Routen T, Shao AF, et al. Using an eIMCI-derived decision support protocol to improve provider-caretaker communication for treatment of children under 5 in Tanzania. *Glob Health Commun*. 2015;1(1):41–47.

Rahman A, Akhtar P, Hamdani SU, Atif N, Nazir H, Uddin I, et al. Using technology to scale-up training and supervision of community health workers in the psychosocial management of perinatal depression: a non-inferiority, randomized controlled trial. *Glob Ment Health*. 2019;6:e8.

Rowe AK, Rowe SY, Peters DH, Holloway KA, Chalker J, Ross-Degnan D. The effectiveness of strategies to improve health worker knowledge in low- and middle-income countries and the association between knowledge and clinical practice: a systematic review. Poster presentation by AK Rowe at the 66th Annual Meeting of the ASTMH, November 5–9, 2017, Baltimore, Maryland. Abstract 233.

Rowe SY, Peters DH, Holloway KA, Chalker J, Ross-Degnan D, Rowe AK. A systematic review of the effectiveness of strategies to improve health care provider performance in low- and middle-income countries: Methods and descriptive results. *PLoS One.* 2019;14(5):e0217617.

Sarrassat S, Lewis JJ, Some AS, Somda S, Cousens S, Blanchet K. An Integrated eDiagnosis Approach (IeDA) versus standard IMCI for assessing and managing childhood illness in Burkina Faso: a stepped-wedge cluster randomised trial. *BMC Health Serv Res*. 2021;21:354.

Soremekun S, Källander K, Lingam R, et al. Improving outcomes for children with malaria, diarrhoea and pneumonia in Mozambique: A cluster randomised controlled trial of the inSCALE technology innovation. *PLoS Digit Health*. 2023;2(6):e0000235. Personal communication from Seyi Soremekun, London School of Hygiene and Tropical Medicine, on February 4, 2025.

Toda M, Njeru I, Zurovac D, O-Tipo S, Kareko D, Mwau M, Morita K. Effectiveness of a Mobile Short-Message-Service-Based Disease Outbreak Alert System in Kenya. *Emerg Infect Dis*. 2016;22(4):711-5. Erratum in: *Emerg Infect Dis*. 2016;22(8):1523.

Toda M, Njeru I, Zurovac D, Kareko D, O-Tipo S, Mwau M, Morita K. Understanding mSOS: A qualitative study examining the implementation of a text-messaging outbreak alert system in rural Kenya. *PLoS One*. 2017;12(6):e0179408.

United States Agency for International Development. Digital Approaches to Supportive Supervision: Guidance Framework. 2023. Accessed December 29, 2024. https://chisuprogram.org/resources/digital-approaches-supportive-supervision-guidance-framework

Venkateswaran M, Ghanem B, Abbas E, et al. A digital health registry with clinical decision support for improving quality of antenatal care in Palestine (eRegQual): a pragmatic, cluster-randomised, controlled, superiority trial. *Lancet Digit Health*. 2022;4:e126–e136.

Whidden C, Kayentao K, Liu JX, et al. Improving community health worker performance by using a personalised feedback dashboard for supervision: a randomised controlled trial. *J Glob Health*. 2018;8:020418.

World Health Organization. WHO guideline: recommendations on digital interventions for health system strengthening. Geneva: World Health Organization: 2019. Accessed April 15, 2023. https://www.who.int/publications/i/item/9789241550505.

Yang JE, Lassala D, Liu JX, Whidden C, Holeman I, Keita Y, et al. Effect of mobile application user interface improvements on minimum expected home visit coverage by community health workers in Mali: a randomised controlled trial. *BMJ Glob Health*. 2021;6(11):e007205.

Zafar S, Sikander S, Hamdani SU, Atif N, Akhtar P, Nazir H, et al. The effectiveness of technology-assisted cascade training and supervision of community health workers in delivering the Thinking Healthy Program for perinatal depression in a post-conflict area of Pakistan - study protocol for a randomized controlled trial. *Trials*. 2016;17:188.

Zakus D, Moussa M, Ezechiel M, Yimbesalu JP, Orkar P, Damecour C, Ghee AE, MacFarlane M, Nganga G. Clinical evaluation of the use of an mhealth intervention on quality of care provided by Community Health Workers in southwest Niger. *J Glob Health*. 2019;9(1):010812.

Zarekar M, Al-Shehabi H, Dörner R, Weishaar H, Lennemann T, El Bcheraoui C, et al. The impact of information and communication technology on immunisation and immunisation programmes in low-income and middle-income countries: a systematic review and meta-analysis. *EBioMedicine*. 2025;111:105520.

Section 5. The correlation between health worker practice outcomes and patient health outcomes

Text S9. Background, objective, methods, and results

BACKGROUND

The ultimate goal of health programs is to improve health outcomes. However, in low- and middle-income countries (LMICs), measuring health outcomes with reasonably good validity is usually expensive and technically complex. Compared to health outcomes, measuring the quality of healthcare provider (HCP) practices (e.g., correct diagnosis and treatment) is usually less expensive and complex. Moreover, studies of the effectiveness of interventions to improve HCP performance are much more likely to measure HCP practices than health outcomes. Therefore, an important methodological question is: how well do improvements in HCP practice outcomes serve as a proxy for improvements in health outcomes? This analysis represents an expansion and update of that published by Rowe et al. in 2018.

OBJECTIVE

Estimate the correlation between changes in HCP practice outcomes and changes in patient health outcomes in LMICs.

METHODS

- Overview. This analysis has two basic steps. In step 1, studies of the effectiveness of interventions to improve HCP performance in LMICs that measured both HCP practice outcomes and patient health outcomes were identified from literature reviews. Each of these studies has intervention effect sizes for at least one HCP practice outcome and at least one patient health outcome. An effect size represents how much the intervention changed a study outcome—e.g., an HCP training intervention might improve HCP practices by 7 percentage points (%-pts). In step 2, the correlation between changes in HCP practice outcomes and changes in patient health outcomes was estimated.

- Definition of HCP. Health workers that provide health care to patients or that perform other health-related activities (e.g., educating patients or community members) in a hospital, outpatient clinic, or community setting.

- Inclusion criteria. Eligible studies: 1) were done in an LMIC, 2) examined the quantitative effect of an intervention to improve HCP performance on an eligible HCP practice outcome and an eligible patient health outcome, and 3) used an eligible study design. Eligible HCP practice outcomes (or processes of care) were correct patient assessment, diagnosis, treatment, counseling, referral, preventive services, and documentation (which reflect behaviors when managing real patients). Eligible patient health outcomes were measures of morbidity and mortality of patients or community members. Eligible study designs were: 1) controlled before-and-after study (with or without randomization), 2) post-intervention-only study with randomized controls, or 3) interrupted time series.

- Literature search. This analysis identified studies from two literature reviews. The first review was the Health Care Provider Performance Review (HCPPR), which used an in-depth literature search up through 2016. Detailed methods are described in Rowe et al. 2019. The second was the rapid review of low-dose, high-frequency training (LDHFT). Detailed methods are described in **Text S7** in the **Online Supplementary Document**.

- Data abstraction. Effect sizes from eligible outcomes were abstracted from the original study reports, which are listed in the table below. Effect sizes from the HCPPR were independently abstracted by two researchers [Rowe 2019], and effect sizes from the rapid review of LDHFT were abstracted by one person (AKR).

- Risk of bias (ROB) assessment. The ROB was based on the study design and study limitations. For details see methods for the HCPPR [Rowe 2019] and the rapid review of LDHFT (**Text S7** in the **Online Supplementary Document**).

- Analysis. Effect sizes were measured in terms of absolute %-pt changes. For outcomes measured as a percentage that could plausibly change over the entire range from 0 to 100% (i.e., most HCP practice outcomes, such as percentage of children weighed), the effect size = $(\text{follow-up} - \text{baseline})_{\text{intervention}} - (\text{follow-up} - \text{baseline})_{\text{controls}}$. For continuous unbounded outcomes and for percentage outcomes that are only expected to change over a narrow range (e.g., percentage of women with postpartum hemorrhage), the effect size = 100% x $\{[(\text{follow-up} - \text{baseline})/\text{baseline}]_{\text{intervention}} - [(\text{follow-up} - \text{baseline})/\text{baseline}]_{\text{controls}}\}$. Often, studies did not present effect sizes calculated this way, and the reports did not include baseline and follow-up outcome values that could be used to calculate effect sizes. Instead, studies reported similar kinds of effect sizes (e.g., difference-in-differences effects, or odds ratios for health outcomes) that were estimated with statistical regression models that adjusted for potential confounders and clustering. When available, these model-based effect size estimates were used. For each study comparison, the median of effect sizes (MES) from HCP practice outcomes was calculated, and (separately) the MES from patient health outcomes was calculated. HCP practice-health outcome MES pairs were plotted graphically, and Pearson's correlation coefficient was estimated. Pearson correlation coefficients were interpreted as: very high positive correlation (coefficient values of 0.9–1.0), high (0.7–0.9), moderate (0.5–0.7), low (0.3–0.5), and very low (0–0.3). Simple linear regression was performed to predict patient health values (dependent variable) based on an HCP practice outcome value (independent variable). The main analysis included studies with all ROB levels, and a sensitivity analysis only included studies with low or moderate ROB.

RESULTS

- In the main analysis, 25 study comparisons (and thus 25 MES values of HCP practice outcomes and 25 MES values of patient health outcomes) were included from 22 LMICs (**Table S7** in the **Online Supplementary Document**, below). Nine study comparisons had a low ROB, four had a moderate ROB, seven had a high ROB, and five had a very high ROB.

- The correlation between improved HCP practices and improved patient health outcomes was moderate. In the main analysis with all 25 study comparisons, Pearson's correlation coefficient was 0.664 (P-value = 0.0003) (**Figure S1** in the **Online Supplementary Document**, below). The regression line equation was: $\hat{Y}$ (health outcome MES) = 1.564 + 0.774 x (HCP practice outcome MES). The sensitivity analysis that only included studies with a low or moderate risk of bias had 13 study comparisons. Pearson's correlation coefficient was 0.698 (P-value = 0.008) (**Figure S2** in the **Online Supplementary Document**, below). The regression line equation was: $\hat{Y}$ (health outcome MES) = 4.413 + 0.637 x (HCP practice outcome MES).

Table S7. Studies included in an analysis of the correlation between changes in healthcare provider (HCP) practice outcomes and changes in patient health outcomes in low- and middle-income countries

Country	Reference	Median effect size for HCP practice outcomes	Median effect size for patient health outcomes	Risk of bias
Argentina, Brazil, Cuba, and Mexico	Belizán JM, Barros F, Langer A, Farnot U, Victora C, Villar J. Impact of health education during pregnancy on behavior and utilization of health resources. Latin American Network for Perinatal and Reproductive Research. *Am J Obstet Gynecol*. 1995;173(3 Pt 1):894–899.	–1.3 %-pts	0.2 %-pts	Low
Bangladesh	Personal communication from Shams El Arifeen, International Centre for Diarrhoeal Diseases Research, Bangladesh (sarifeen@siriusbb.com; shams@icddrb.org) to Samantha Rowe, Centers for Disease Control and Prevention (say9@cdc.gov) on June 13, 2007, which contained a draft manuscript and supplementary tables of Bangladesh MCE Health Facility Survey Indicators.	42.0 %-pts	13.5 %-pts	Moderate
China	Huang J, Jiang D, Wang X, Liu Y, Fennie K, Burgess J, Williams AB. Changing knowledge, behavior, and practice related to universal precautions among hospital nurses in China. *The Journal of Continuing Education in Nursing*. 2002; 33(5):217–224.	42.8 %-pts	11.8 %-pts	Moderate
Ghana	Ofei F, Forson A, Tetteh R, Ofori-Adjei D. An intervention to improve antibiotic prescribing habits of doctors in a teaching hospital. Presented at: Second International Conference on Improving Use of Medicines (ICIUM); March 30 – April 2, 2004; Chiang Mai, Thailand [cited April 10, 2010]. 13 p. Available at: http://www.icium.org/icium2004/resources/ppt/AD029.ppt	–20.3 %-pts	43.7 %-pts	Very high
Guatemala	Bailey PE, Szaszdi JA, Schieber B. Analysis of the vital events reporting system of the Maternal and Neonatal Health Project, Quetzaltenango, Guatemala. Technical Working Paper 3. December 1994. Working Paper prepared for USAID, Contract No. DPE 5966-Z-00-8083-00. Available at: http://pdf.usaid.gov/pdf_docs/PNACE151.pdf, accessed January 14, 2009	3.0 %-pts	7.5 %-pts	High
India	Bhandari N, Mazumder S, Bahl R, Martines J, Black RE, Bhan MK, and the Infant feeding study group. An educational intervention to promote appropriate complementary feeding practices and physical growth in infants and young children in rural Haryana, India. *Journal of Nutrition*. 2004; 134: 2342–2348.	39.1 %-pts	0 %-pts	High
Kenya	Shikuku DN, Mukosa R, Peru T, Yaite A, Ambuchi J, Sisimwo K. Reducing intrapartum fetal deaths through low-dose high frequency clinical mentorship in a rural hospital in Western Kenya: a quasi-experimental study. *BMC Pregnancy Childbirth*. 2019;19(1):518.	109.1 %-pts	80.4 %-pts	Low

Country	Reference	Median effect size for HCP practice outcomes	Median effect size for patient health outcomes	Risk of bias
Kenya, Nigeria, South Africa, and Tanzania	Gallos I, Devall A, Martin J, Middleton L, Beeson L, Galadanci H, et al. Randomized trial of early detection and treatment of postpartum hemorrhage. *N Engl J Med*. 2023;389(1):11–21.	51.8 %-pts	60.0 %-pts	Low
Kenya and Uganda	Walker D, Otieno P, Butrick E, et al. Effect of a quality improvement package for intrapartum and immediate newborn care on fresh stillbirth and neonatal mortality among preterm and low-birthweight babies in Kenya and Uganda: A cluster-randomised facility-based trial. *Lancet Glob Health*. 2020; 8(8): e1061–e1070.	33.0 %-pts	34.0 %-pts	Low
Mali and Senegal	Dumont A, Fournier P, Abrahamowicz M, Traore M, Haddad S, Fraser WD. Quality of care, risk management, and technology in obstetrics to reduce hospital-based maternal mortality in Senegal and Mali (QUARITE): A cluster-randomised trial. *Lancet*. 2013;382:146–157.	16.5 %-pts	15.0 %-pts	Moderate
Mexico	Arreola-Risa C, Mock C, Herrera-Escamilla AJ, Contreras I, Vargas J. Cost-effectiveness and benefit of alternatives to improve training for prehospital trauma care in Mexico. *Prehospital and Disaster Medicine*. 2004;19(4):318–325.	5.0 %-pts	1.3 %-pts	Very high
Mexico	Fritz J, Walker DM, Cohen S, Angeles G, Lamadrid-Figueroa H. Can a simulation-based training program impact the use of evidence based routine practices at birth? Results of a hospital-based cluster randomized trial in Mexico. *PLOS One*. 2017;12(3):e0172623.	11.9 %-pts	32.4 %-pts	Moderate
Nepal	Kc A, Ewald U, Basnet O, et al. Effect of a scaled-up neonatal resuscitation quality improvement package on intrapartum-related mortality in Nepal: a stepped-wedge cluster randomized controlled trial. *PLoS Med*. 2019;16:e1002900.	52.0 %-pts	21.0 %-pts	Low
Nicaragua	Rawlings LB, Rubio GM. Evaluating the impact of conditional cash transfer programs: lessons from Latin America. 2003. World Bank, Policy Research Working Paper Series, number 3119. available at: http://www-wds.worldbank.org/external/default/WDSContentServer/IW3P/IB/2003/09/30/000094946_0309160409264/Rendered/PDF/multi0page.pdf, accessed January 19, 2010.	25.6 %-pts	1.5 %-pts	Low
Niger	Catsambas TT, Franco LM, Gutmann M, et al. Evaluating health care collaboratives: the experience of the Quality Assurance Project. Bethesda (MD): University Research Co., LLC (URC); 2008 June. 89 p. Collaborative Evaluation Series. Published by the USAID Health Care Improvement Project.	66.0 %-pts	96.2 %-pts	High
Niger	Catsambas TT, Franco LM, Gutmann M, et al. Evaluating health care collaboratives: the experience of the Quality Assurance Project. Bethesda (MD): University Research Co., LLC (URC); 2008 June. 89 p. Collaborative Evaluation Series. Published by the USAID Health Care Improvement Project.	87.7 %-pts	127.1 %-pts	Very high

Country	Reference	Median effect size for HCP practice outcomes	Median effect size for patient health outcomes	Risk of bias
Niger	Catsambas TT, Franco LM, Gutmann M, et al. Evaluating health care collaboratives: the experience of the Quality Assurance Project. Bethesda (MD): University Research Co., LLC (URC); 2008 June. 89 p. Collaborative Evaluation Series. Published by the USAID Health Care Improvement Project.	35.3 %-pts	–9.8 %-pts	Very high
Pakistan	Zaman S, Ashraf RN, Martines J. Training in complementary feeding counselling of healthcare workers and its influence on maternal behaviours and child growth: a cluster randomized controlled trial in Lahore, Pakistan. *Journal of Health Population and Nutrition*. 2008;26(2):210–222.	25.1 %-pts	31.7 %-pts	Low
Russia	Catsambas TT, Franco LM, Gutmann M, et al. Evaluating health care collaboratives: the experience of the Quality Assurance Project. Bethesda (MD): University Research Co., LLC (URC); 2008 June. 89 p. Collaborative Evaluation Series. Published by the USAID Health Care Improvement Project.	4.3 %-pts	–29.3 %-pts	High
Russia	Catsambas TT, Franco LM, Gutmann M, et al. Evaluating health care collaboratives: the experience of the Quality Assurance Project. Bethesda (MD): University Research Co., LLC (URC); 2008 June. 89 p. Collaborative Evaluation Series. Published by the USAID Health Care Improvement Project.	–22.9 %-pts	–8.2 %-pts	High
South Africa	van Zyl DG, Rheeder P. Physician education programme improves quality of diabetes care. *S Afr Med J*. 2004;94(6):455–459.	42.7 %-pts	2.3 %-pts	High
Sri Lanka	Senarath U, Fernando D, Rodrigo I. Effect of training for care providers on practice of essential newborn care in hospitals in Sri Lanka. *Journal of Obstetric Gynecologic and Neonatal Nursing*. 2007;36(6):531–541.	20.5 %-pts	3.5 %-pts	High
Tanzania	Alwy Al-beity F, Pembe A, Hirose A, et al. Effect of the competency-based Helping Mothers Survive Bleeding after Birth (HMS BAB) training on maternal morbidity: A cluster-randomised trial in 20 districts in Tanzania. *BMJ Global Health*. 2019;4:e001214.	0.2 %-pts	42.2 %-pts	Low
Uganda	Catsambas TT, Franco LM, Gutmann M, et al. Evaluating health care collaboratives: the experience of the Quality Assurance Project. Bethesda (MD): University Research Co., LLC (URC); 2008 June. 89 p. Collaborative Evaluation Series. Published by the USAID Health Care Improvement Project.	–5.0 %-pts	0.3 %-pts	Very high
Uganda	Hanson C, Atuhairwe S, Lucy Atim J, Marrone G, Morris JL, Kaharuza F. Effects of the Helping Mothers Survive Bleeding after Birth training on near miss morbidity and mortality in Uganda: A cluster-randomized trial. *Int J Gynecol Obstet*. 2021;152(3):386–394.	–8.0 %-pts	–31.1 %-pts	Low

Figure S1. The correlation between changes in healthcare provider practice outcomes and changes in patient health outcomes in low- and middle-income countries (main analysis that includes all risk of bias levels)

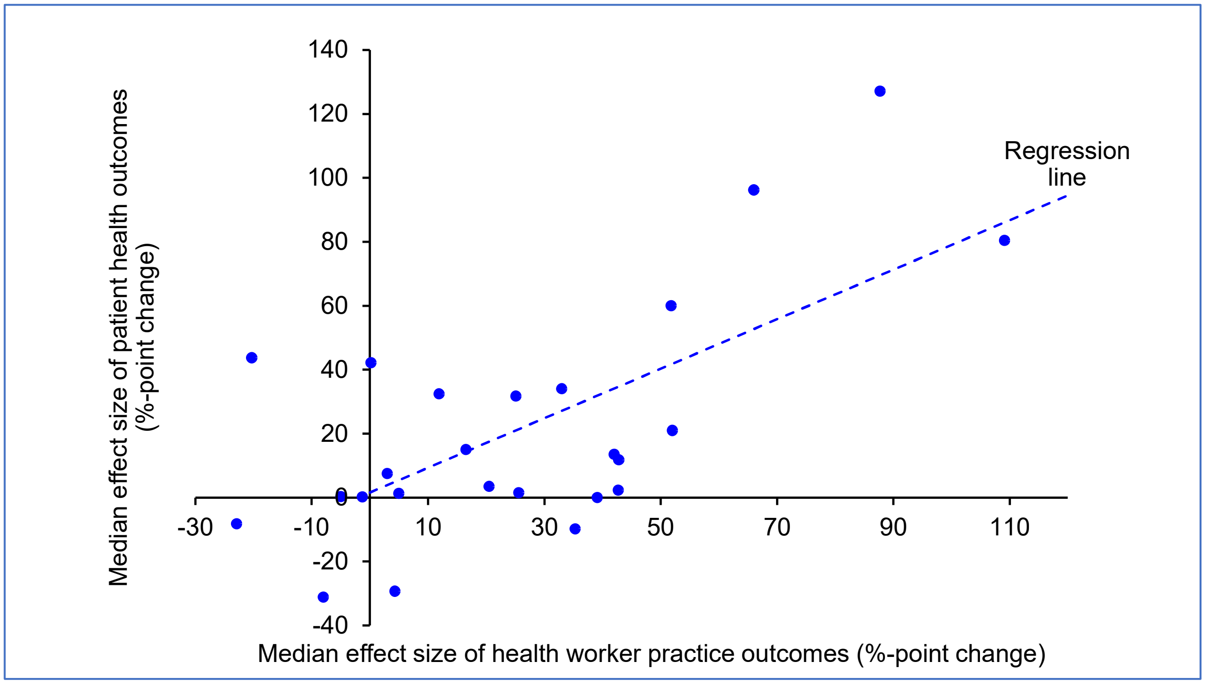

Figure S2. The correlation between changes in healthcare provider practice outcomes and changes in patient health outcomes in low- and middle-income countries (sensitivity analysis that includes only studies with a low or moderate risk of bias)

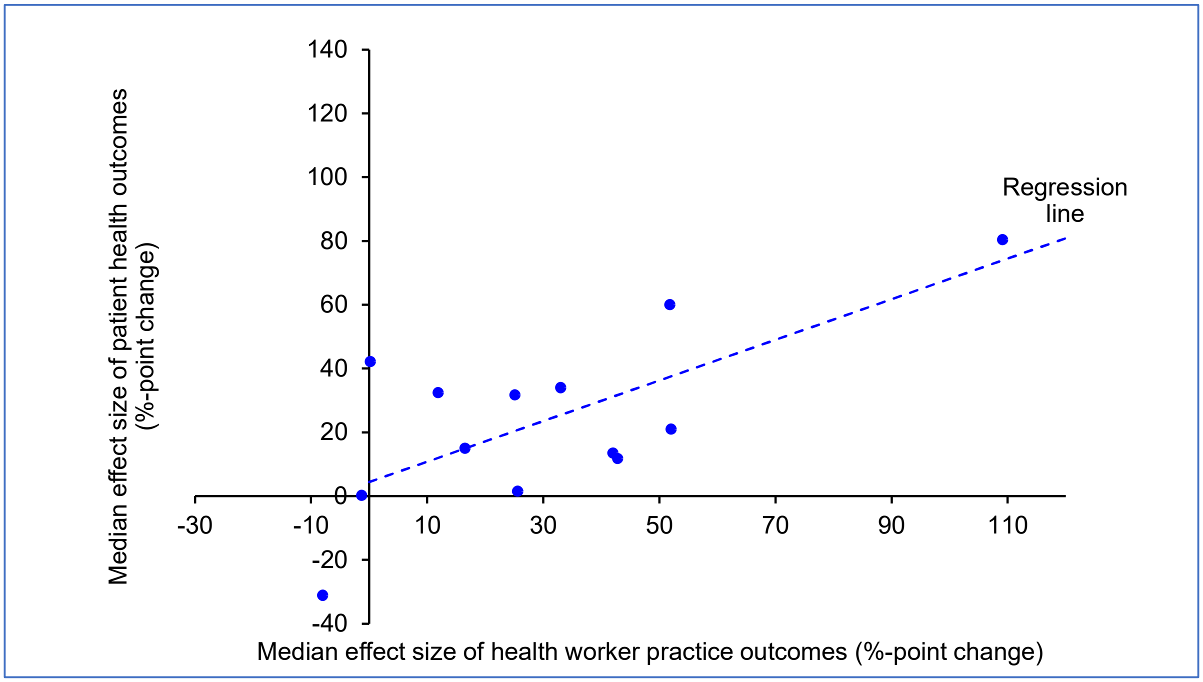

REFERENCES

Rowe AK, Labadie G, Jackson D, Vivas-Torrealba C, Simon J. Improving health worker performance: an ongoing challenge for meeting the sustainable development goals. *BMJ*. 2018; 362:k2813.

Rowe SY, Peters DH, Holloway KA, Chalker J, Ross-Degnan D, Rowe AK. A systematic review of the effectiveness of strategies to improve health care provider performance in low- and middle-income countries: Methods and descriptive results. *PLoS One.* 2019;14(5):e0217617.
